# Chemoenzymatic Synthesis of Optically Active Ethereal Analog of *iso*-Moramide—A Novel Potentially Powerful Analgesic †

**DOI:** 10.3390/ijms231911803

**Published:** 2022-10-05

**Authors:** Paweł Borowiecki

**Affiliations:** Laboratory of Biocatalysis and Biotransformation, Department of Drugs Technology and Biotechnology, Faculty of Chemistry, Warsaw University of Technology, Koszykowa St. 75, 00-662 Warsaw, Poland; pawel.borowiecki@pw.edu.pl; Tel.: +48-(22)-234-7677

**Keywords:** biocatalysis, lipases, kinetic resolution, transesterification, Mosher-type CDAs, phase transfer catalysis (PTC), chiral analgesics, docking studies, opioid receptors (ORs)

## Abstract

To develop potent and safer analgesics, we designed and synthesized a novel enantiomerically enriched ethereal analog of (*R*)-*iso*-moramide, namely 2-[(2*R*)-2-(morpholin-4-yl)propoxy]-2,2-diphenyl-1-(pyrrolidin-1-yl)ethan-1-one. The titled active agent can potentially serve as a powerful synthetic opiate with an improved affinity and selectivity toward opioid receptors (ORs). This hypothesis was postulated based on docking studies regarding the respective complexes between the designed ligand and *µ*-OR, *δ*-OR, and *κ*-OR. The key step of the elaborated asymmetric synthesis of novel analog involves lipase-catalyzed kinetic resolution of racemic 1-(morpholin-4-yl)propan-2-ol, which was accomplished on a 10 g scale via an enantioselective transesterification employing vinyl acetate as an irreversible acyl donor in *tert*-butyl methyl ether (MTBE) as the co-solvent. Next, the obtained homochiral (*S*)-(+)-morpholino-alcohol (>99% ee) was functionalized into corresponding chloro-derivative using thionyl chloride (SOCl_2_) or the Appel reaction conditions. Further transformation with *N*-diphenylacetyl-1-pyrrolidine under phase-transfer catalysis (PTC) conditions using O_2_-saturated DMSO/NaOH mixture as an oxidant furnished the desired levorotatory isomer of the title product isolated in 26% total yield after three steps, and with 89% ee. The absolute configuration of the key-intermediate of (*R*)-(–)-*iso*-moramide was determined using a modified form of Mosher’s methodology. The preparation of the optically active dextrorotatory isomer of the titled product (87% ee) was carried out essentially by the same route, utilizing (*R*)-(–)-1-(morpholin-4-yl)propan-2-ol (98% ee) as a key intermediate. The spectroscopic characterization of the ethereal analog of *iso*-moramide and the enantioselective retention relationship of its enantiomers using HPLC on the cellulose-based chiral stationary phase were performed. Moreover, as a proof-of-principle, single-crystal X-ray diffraction (XRD) analysis of the synthesized 2-[(2*R*)-2-(morpholin-4-yl)propoxy]-2,2-diphenyl-1-(pyrrolidin-1-yl)ethan-1-one is reported.

## 1. Introduction

**Figure 1:**
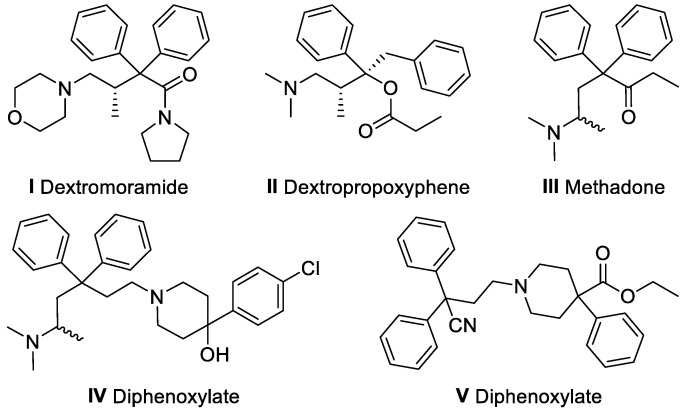
The structural resemblance between dextromoramide (**I**) and other morphine-like analgesics (**II**,**III**) and antidiarrheals (**IV**,**V**).

The treatment of chronic pain, including acute, post-traumatic, post-operative, post-heart attack, cancer and/or terminal pain is the critical domain of potent analgesics from the group of opioids (the so-called “drugs of the third rung of the WHO analgesic ladder”) [1]. Among the most notable examples of opiate analgesics, which belong to full agonists of *µ*-type opioid receptors, are morphine, fentanyl, hydromorphone, oxymorphone, pethidine (meperidine), methadone, buprenorphine, tapentadol, levorphanol, and oxycodone [2,3]. Apart from the drugs mentioned above, dextromoramide (marketed under trade names: *Palfium*^®^, *Jetrium*^®^, *Pyrrolamidol*^®^, *Dimorlin*^®^, etc.) constitutes a potent synthetic opiate, which is structurally related to dextropropoxyphene, methadone, loperamide, and diphenoxylate (Figure 1).

Dextromoramide was introduced in 1956 by Dr. Paul Janssen [4,5,6,7], and shortly after discovering that it is a *μ*-agonist analgesic two to four times more powerful than morphine [8], it became a popular drug in pharmacologic pain therapy. Unfortunately, although dextromoramide is an ultra-efficient pharmaceutical against violent (intense) pain that standard doses of other opiates can hardly alleviate, it suffers from two major limitations: it is highly addictive and induces respiratory depression [9]. Therefore, a serious risk of dependence and apnoea caused by dextromoramide led us to develop a novel analog of this drug, which is envisioned to be a potentially more effective and safer analgesic with a much lower tolerability factor than the parent molecule. The desired analog, namely 2-[(2*R*)-2-(morpholin-4-yl)propoxy]-2,2-diphenyl-1-(pyrrolidin-1-yl)ethan-1-one, was designed based on the in silico docking studies and further synthesized using (*S*)-1-(morpholin-4-yl)propan-2-ol as the key intermediate. In turn, preparing this alcohol on a multi-gram scale and in enantiomerically pure form is a very useful task because it constitutes the critical precursor in the formal synthesis of dextromoramide and also represents a valuable template for other promising compounds that can interact with a large variety of biological targets. In this regard, the structural motif of 1-(morpholin-4-yl)propan-2-ol plays a crucial role in the synthesis of inhibitors of several enzymes, including acetylcholinesterase (ACE) [10,11], human neutrophil elastase [12], kynurenine-3-monooxygenase (KMO) [13,14], PI3 kinase [15], tyrosine Met kinase [16], etc. Moreover, the agonistic activity toward various receptors, including complement component 5a (C5a) [17], melanin-concentrating hormone (MCHr1) [18], subtype of 5-hydroxytryptamine (5-HT_2C_) [19], metabotropic glutamate (mGluR1) [20], G protein-coupled (GPR119) [21], oxytocin (OXTR) [22], 5-hydroxytryptamine (5-HT_1B/1D_) [23], metabotropic L-glutamate (mGluR2) [24], were discovered employing this scaffold. Recently, biologically active compounds possessing that particular morpholino-alcohol moiety were considered emerging drug candidates for diabetes mellitus [25]. Finally, the successful evaluations of 1-(morpholin-4-yl)propan-2-ol-based derivatives for their anti-hypertensive [26], anti-inflammatory [27], and lipid bi-layers membrane [28] activities show that there is a convincing potency for the application of this compound not only in the synthesis of non-morphinan painkillers but in a wider scope of medicinal chemistry as well. 

In the present work, inspired by the unique analgetic properties of dextromoramide, a novel structural analog, potentially selective for *κ*-opioid receptors, and thus exhibiting reduced adverse pharmacological effects, was designed based on molecular docking studies and synthesized utilizing chemoenzymatic methodology. A critical synthetic step, including lipase-catalyzed kinetic resolution of racemic 1-(morpholin-4-yl)propan-2-ol, was optimized to achieve desired (*R*)-intermediate on a multigram scale. After further functionalization of the key precursor, the chemical structure and the absolute configuration of the novel (*R*)-*iso*-moramide analog was confirmed by X-ray diffraction analysis.

## 2. Results and Discussion

### 2.1. Molecular Docking Studies

One of medicinal chemistry’s prime goals is to discover new opioid drug candidates with highly desirable features such as improved efficacy and specificity, increased duration of the activity, lower dosage, decreased adverse effects, and reduced risk of developing tolerance and/or addiction [29]. Unfortunately, the difficulty in obtaining selective modulators of opioid receptors (ORs) mainly stems from the phylogenetic proximity of the target proteins, leading to a high structural similarity—over 60% amino acid sequence identity was confirmed [30]. In this context, among the 14 essential amino acid residues found in highly specific orthosteric binding sites located around the top of the central channel formed by a typical 7-pass transmembrane α-helices, 9 of them are evolutionarily highly conserved among all three classical members of the opioid receptor family (*µ*-OR, *δ*-OR, and *κ*-OR) [31]. Moreover, in the case of *µ*- and *δ*-type receptors, the sequence similarity within the binding site is even more remarkable—the 11 amino acids out of 14 being identical.

From the point of view of the efficacy of pain inhibition, the mechanisms concerning interactions between endogenous and exogenous opioids, particularly *µ*-OR and *δ*-OR, play a significant role [32,33]. An effective stimulation of *µ*-OR and *δ*-OR has a complex effect on the release of neurotransmitters involved in pain relief, such as tachykinins, excitatory amino acids (EAA), neuropeptides (substance P (SP)), and calcitonin gene-related peptide (CGRP) [34,35]. However, over-stimulation of *µ*-OR leads to life-threatening respiratory depression, which is not observed in the case of selective agonists of *δ*-OR and *κ*-OR. Moreover, selective *κ*-receptor antagonists, in addition to their proven analgesic and anti-inflammatory properties used in treating peritoneal symptoms or chronic neuropathic pain, also exhibit psychomimetic effects, allowing the treatment of drug-resistant depression, anxiety, social phobia or cocaine and nicotine dependency [36,37]. It is also important to mention that agonists of *κ*-OR with the most prominent examples, such as nalfurafine and difelikefalin (formerly CR845), are promising antipruritic (anti-itch) agents [38,39]. Therefore, ligands that display excellent selectivity and efficacy at kappa-opioid receptors (*κ*-OR) are highly desirable since they may overcome typical drawbacks regarding the side effects of opioid therapy in humans.

**Figure 2:**
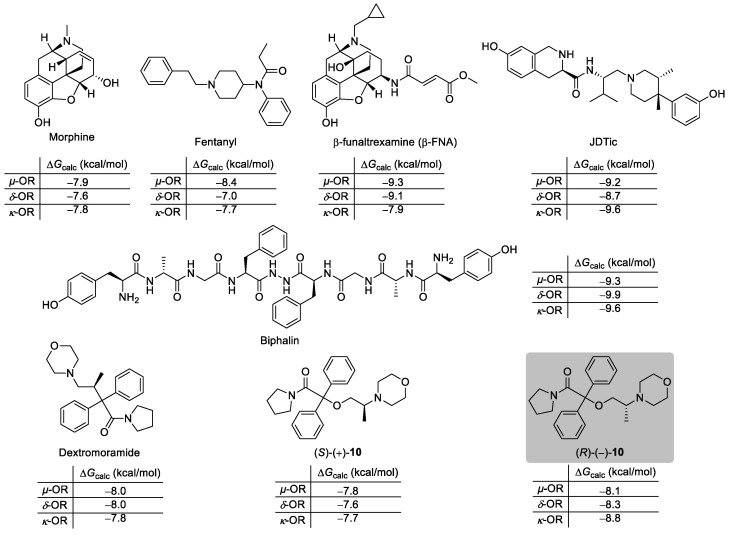
The values of the absolute free binding energies (Δ*G*_calc_ (kcal/mol)) obtained using molecular docking performed for both enantiomers of ethereal analog of *iso*-moramide (*S*)-(+)-**10** and (*R*)-(–)-**10** and benchmark opioids (i.e., morphine, fentanyl, dextromoramide, etc.) complexed with *µ*-OR, *δ*-OR, and *κ*-OR, respectively. The results represent only the calculations for each ligand’s most energetically favorable poses, whereas the rest of these data are included in the Supplementary Materials to enhance readability.

Intending to develop a novel selective non-morphinan kappa agonist, we have virtually screened a set of different ligands toward *µ*-OR (PDB ID: 4DKL [40]), *δ*-OR (PDB ID: 4EJ4 [41]), and *κ*-OR (PDB ID: 4DJH [42]), respectively. All the in silico studied ligands were designed based on dextromoramide as a lead structure. The preliminary results of molecular docking, obtained using non-commercial AutoDock Vina software (v1.1.2) [43], employing standard protocol for enzyme−ligand docking (for details, see Experimental Section 3), revealed that 2-(2-(morpholin-4-yl)propoxy)-2,2-diphenyl-1-(pyrrolidin-1-yl)ethan-1-one (*rac*-**10**) exhibited a very high affinity toward all three types of opioid receptors with the highest being observed for *κ*-OR. Therefore, in the next step of computational investigations, a direct comparison of the absolute free binding energies [Δ*G*_calc_ (kcal/mol)] of the complexes’ formation between the respective ORs and both enantiomers of the selected candidate (*S*)-(+)-**10** and (*R*)-(–)-**10** as well as dextromoramide parent molecule and the series of the model opioids, including morphine, fentanyl, β-funaltrexamine (β-FNA), JDTic, and biphalin, was performed (Figure 2).

After a detailed examination of the molecular docking results, it was clear that the newly designed analog (*R*)-(–)-**10** outperformed most of the well-known clinical drugs, except JDTic and biphalin, in terms of the mutual ligand-protein affinity to *κ*-OR (Δ*G*_calc_ = −8.8 kcal/mol). When considering the binding affinity toward *δ*-OR, docking studies showed that (*R*)-(–)-**10** was superior to dextromoramide, morphine, and fentanyl. In turn, (*R*)-(–)-**10** exhibited almost an identical affinity for *µ*-type opioid receptors as dextromoramide; however, it seemed to be significantly less potent toward *µ*-OR when compared with the remaining opioids except for morphine. It is noteworthy that the absolute configuration of the asymmetric carbon atom presented in chiral ligand **10** is likely to display a critical role in the pharmacological activity since docking calculations confirmed that the opposite (*S*)-enantiomer was less efficient in binding with all the studied ORs (Δ*G*_calc_ = from −7.6 to −7.8 kcal/mol). This phenomenon could be attributed to steric clashes of the bulky diphenyl substituent and fewer hydrogen bonds formed between (*S*)-(+)-**10** within the receptor molecules (not shown herein). 

**Figure 3:**
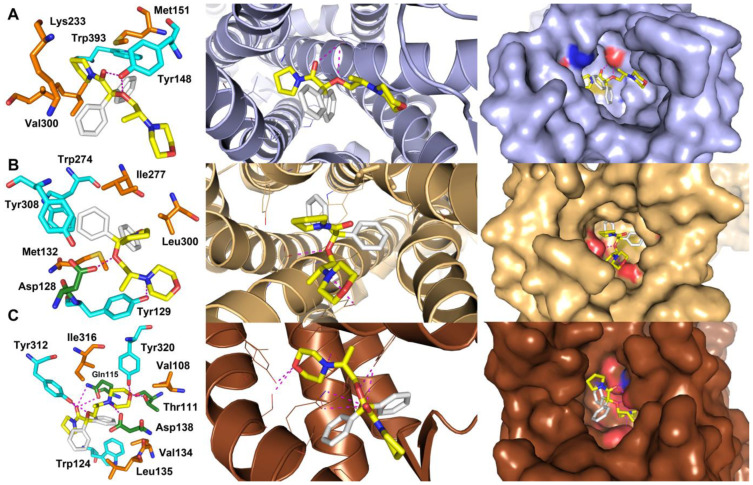
Representative binding modes for the lowest binding-energy complexes of ethereal analog of *iso*-moramide (*R*)-(–)-**10** (yellow-white sticks) with (**A**) *µ*-type opioid receptor (PDB ID: 4DKL) (**B**) *δ*-type opioid receptor (PDB ID: 4EJ4) and (**C**) *κ*-type opioid receptor (PDB ID: 4DJH). The most significant amino acid residues contributing to the stabilization of the ligand molecules in the complexes with all three types of ORs are shown in sticks representations (left column); the key amino acids responsible for polar interactions, including hydrogen bonding, are shown in cyan or green (which in addition indicates the closest H-bonding); for CH–CH van der Waals (vdW) interactions are shown in dark orange. The overall receptor structures are shown as a cartoon diagram (middle column) or surface representation (right column), where *µ*-OR is light blue, *δ*-OR is gold, and *κ*-OR is brown, respectively. Nitrogen atoms are highlighted in blue, oxygen atoms in red, and sulfur in dark yellow. The formation of intermolecular hydrogen bonds is represented by magenta dashed lines. For clarity, H atoms are omitted.

In the next step of in silico studies, the visualization of the representative energetically favorable ligand (*R*)-(–)-**10** binding modes to all three examined ORs, with close contact with amino acid residues located in the ligand-binding pocket, was analyzed (Figure 3). 

The inspection of the productive poses of (*R*)-(–)-**10** in *µ*-OR showed that the ligand possesses close polar contacts between the carbonyl group and/or ethereal oxygen atom and the phenolic hydroxyl of Tyr148 (Figure 3A). In turn, diphenyl substituent forms hydrophobic sets of interactions with Trp393, Val300, and Lys233, respectively. In the case of the complex of (*R*)-(–)-**10** with *δ*-OR the key H-bonding was found between ethereal oxygen and a carboxylic moiety of Asp128 and nitrogen atom of morpholine moiety and Tyr129 (Figure 3B). In addition, the computational analysis revealed that diphenyl functionality of the ligand (*R*)-(–)-**10** displayed π-stacking interaction with Trp274. Noteworthy is an extensive hydrogen bond network formed between (*R*)-(–)-**10** and *κ*-OR (Figure 3C), which mainly stabilizes the potential complex more efficiently than in the cases of *µ*-OR and *δ*-OR. In this context, the critical polar contact occurred inter the oxygen atom of the carbonyl group present in (*R*)-(–)-**10** and the amide group of Gln115, whereas ethereal oxygen interacted with the phenolic hydroxyl group of the Tyr312 side chain. Furthermore, a hydrogen bond between the nitrogen atom of the morpholine ring and the carboxylic acid group of Asp138 was found. Finally, the oxygen atom of the morpholine ring formed hydrogen bonds with Tyr320 and/or Thr111. All these hydrogen-bonding interactions are in the range of 1.9–2.2 Å. The most reliable binding mode identified for (*R*)-(–)-**10** with *κ*-OR shows that the diphenyl scaffold was involved in a π-stacking interaction with Trp124 and stabilized by hydrophobic Leu135 and Val134 residues.

It is worth noting that in the case of the complex formed between (*R*)-(–)-**10** and *κ*-type opioid receptor, the number of hydrogen bonds was 3-fold higher than for the rest of the analyzed complexes. Moreover, incorporating the ethereal oxygen into the designed analog molecule resulted in the formation of additional hydrogen-bonding interactions with each of the studied receptors, which consequently increased the affinity of (*R*)-(–)-**10** toward ORs when compared to a dextromoramide parent molecule. These original binding modes could be useful in rationalizing the analgesic potency of the designed derivative in its high affinity and selective interactions with all three types of opioid receptors. Nevertheless, further detailed biological studies, including biochemical (protein) assays, are required to verify this hypothesis. 

### 2.2. Synthesis of the Racemic Compounds rac-***3*** and rac-***4a***–***c***

**Scheme 1:**
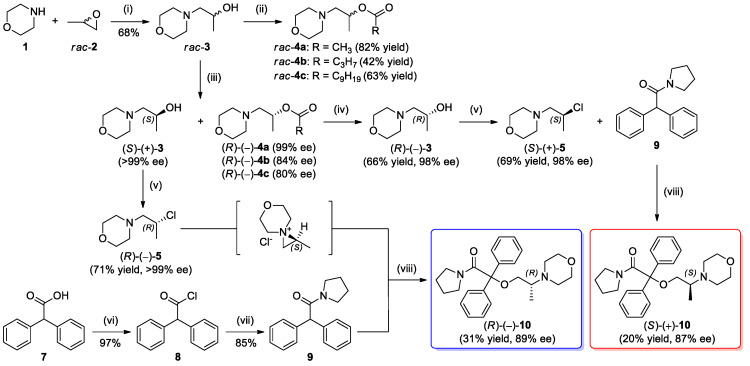
Chemoenzymatic synthesis of enantiomerically enriched ethereal analog of *iso*-moramide (*S*)-(+)-**10** and (*R*)-(–)-**10**. Reagents and conditions: (i) **1** (1.2 equiv), H_2_O, 14 h at 25 °C; (ii) Ac_2_O (1.2 equiv) or C_3_H_7_COCl (1.1 equiv) or C_9_H_19_COCl (1.1 equiv), Et_3_N (1.1–1.2 equiv), DMAP (cat.), dry CH_2_Cl_2_, 12 h at 25 °C; (iii) *rac*-**3** (100 mg/mL conc.), Chirazyme L-2, C-2 (20 mg/mL conc.), vinyl acetate (2 equiv), MTBE (1 mL/0.69 mmol of *rac*-**3**), 16 h (for 1 g-scale) or 27 h (for 10 g-scale), 25 °C, 500 rpm (magnetic stirrer); (iv) NaOH (1.1. equiv), MeOH, 0.5 h at rt; (v) SOCl_2_ (2 equiv), CHCl_3_, 1 h at 0–5 °C, 2 h at rt, 2 h at reflux or CCl_4_ (1.5 equiv), PPh_3_ (1.5 equiv), dry CH_2_Cl_2_, 20 min at 0–5 °C, 2.5 h at rt; (vi) SOCl_2_ (15 equiv), benzene, 6 h at reflux, 24 h at rt; (vii) Pyrrolidine (3.1 equiv), 1,4-dioxane, 50 °C, 1 h; (viii) **5** (1.3 equiv), NaOH (8 equiv), TEBA(Cl) (cat., 10 mol%), dry DMSO, 4 h at 40 °C.

Herein, the development of a novel multi-gram scale enzyme-catalyzed procedure for the kinetic resolution (KR) of racemic 1-(morpholin-4-yl)propan-2-ol (*rac*-**3**) using commercially available immobilized lipases type B from *Candida antarctica* (CAL-B) or *Burkholderia cepacia* (BCL) as the biocatalysts is presented. The critical enzymatic process provided sufficient amounts of each highly enantiomerically enriched alcohols (*S*)-(+)-**3** (>99% ee) and (*R*)-(–)-**3** (98% ee, after further hydrolysis), which were subsequently used as chiral precursors for the synthesis of both enantiomers of titled optically active ethereal analog of *iso*-moramide (*R*)-(–)-**10** and (*S*)-(+)-**10** (Scheme 1).

In the first step, racemic 1-(morpholin-4-yl)propan-2-ol (*rac*-**3**) was synthesized by adopting the procedure reported by Azizi et al. [44] In this regard, treatment of 1,2-propylene oxide **2** with 1.2 equiv of morpholine **1** in water at room temperature resulted in the formation of respective *β*-amino alcohol *rac*-**3**. Under mild reaction conditions, the ring-opening aminolysis of initial epoxide **2** proceeded with excellent regioselectivity and >99% conv., according to gas chromatography (GC) and NMR spectroscopy, respectively. However, after conventional extractive work-up and subsequent column chromatography purification procedure, the morpholine-alcohol *rac*-**3** was isolated in 68% yield. Mass loss of the desired *rac*-**3**, and thus reduction of the reaction yield, might be due to the partial solubility of the product in water. 

In turn, to obtain racemic acyl derivatives *rac*-**4a**–**c** requested for analytical purposes, the β-morpholino-alcohol *rac*-**3** was esterified with acetic anhydride (in the case of *rac*-**4a**) or the corresponding acyl chloride (in the case of *rac*-**4b**–**c**) dissolved in dry dichloromethane in the presence of triethylamine (Et_3_N) as a base and a catalytic amount of 4-dimethylaminopyridine (DMAP). All esters were obtained in moderate-to-high yields (42–82%) after preparative column chromatography. 

### 2.3. Lipase-Catalyzed Kinetic Resolution of rac-***3*** Using Transesterification Methodology

The outcome of the enzymatic kinetic resolution (EKR) of racemic alcohols realized via the transesterification scenario strongly depends on the applied reactions’ conditions. In this case, optimizing the appropriate EKR parameters is a tedious task since it requires a selection of the enzyme biocatalyst and the reaction medium (so-called “medium engineering”), evaluation of the type of acyl group donor, determination of the reaction stoichiometry, setting of time of the process, adjusting operating temperature, etc. The biocatalytic studies presented herein aimed to establish optimal conditions for enantioselective transesterification of racemic 1-(morpholin-4-yl)propan-2-ol (*rac*-**3**) carried out using activated vinyl esters as irreversible acyl donors under kinetically controlled conditions in organic solvents. This task was achieved in a three-step optimization procedure, employing (i) enzyme selection, (ii) solvent screening, and (iii) investigation of the effect of various acyl donors. Next, under the optimal reaction conditions, the process was scaled up to 10 g of the racemic substrate *rac*-**3**, providing sufficient amounts of desired enantiomers (*S*)-(+)-**3** and (*R*)-(–)-**3** (obtained after hydrolysis of the acetate (*R*)-(–)-**4a**) for the synthesis of titled active agent in both stereogenic forms (*S*)-(+)-**10** and (*R*)-(–)-**10**. 

Initial analytical-scale studies were performed to find the most suitable enzyme catalyst for enantioselective transesterification of racemic alcohol *rac*-**3**. In the preliminary screening, eight commonly used commercially available lipase preparations were tested in 20% *w*/*w* with respect to substrate *rac*-**3** and in the presence of an excess of vinyl acetate as an irreversible acyl donor in methyl *tert*-butyl ether (MTBE) at 25 °C (Table 1). 

**Table 1:**
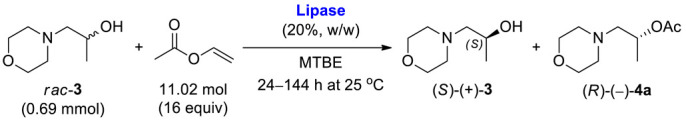
Lipase screening for the kinetically controlled enantioselective acetylation of *rac*-**3** using vinyl acetate.

Entry	Lipase Preparation ^a^	*t* (h)	Conv. (%) ^b^	ee_s_ (%) ^c^	ee_p_ (%) ^d^	*E* ^e^
1	Amano PS	144	41	62	89	32
2	Amano PS-IM	120	55	>99	81	49
3	Amano PS-Immobead 150	120	32	46	>99	314
4	Novozym 435	120	67	>99	49	14
5	Chirazyme L-2, C-2	24	58	>99	72	31
6	Chirazyme L-2, C-3	24	53	95	84	42
7	Amano AK	72	48	85	92	65
8	Chirazyme L-5	72	28	17	44	3

^a^ Conditions: *rac*-**3** 100 mg, lipase 20 mg, MTBE 1 mL, vinyl acetate 948 mg (16 equiv), 25 °C, 500 rpm; ^b^ Determined by GC analysis using a calibration curve; ^c^ Determined by HPLC analysis using a chiral stationary phase; ^d^ Determined by chiral HPLC analysis of the corresponding alcohol (*R*)-(–)-**3** obtained after NaOH-catalyzed methanolysis of (*R*)-(–)-**4a**; ^e^ Calculated according to Chen et al. [45], using the equation: *E* = {ln[(1 − conv.)(1 − ee_s_)]}/{ln[(1 − conv.)(1 + ee_s_)]}.

All the reactions’ progress was monitored using GC. In turn, the evaluation of enantiomeric excesses (% ee) of EKR products was examined using HPLC equipped with columns packed with chiral stationary phases. It is important to note that for the optically active acetate (*R*)-(–)-**4a**, direct HPLC analysis was impossible since enantiomers of this compound were inseparable using various tested cellulose- and/or amylose-based chiral columns. Therefore, to determine the optical purity of (*R*)-(–)-**4a**, enantiomerically enriched acetate had to be hydrolyzed under base methanolysis conditions, and only then the resulting alcohol (*R*)-(–)-**3** was analyzed in terms of enantiomeric excess. As shown in Table 1, Amano PS-Immobead 150 was found to be the most enantioselective lipase toward *rac*-**3** (*E* > 200); however, it catalyzed the resolution of the racemate with a relatively low rate (32% conv. after 120 h). Taking the stereoselectivity and catalytic activity into account, the most efficient biocatalyst for enantioselective acetylation of *rac*-**3** appeared to be Chirazyme L-2, C-2. This lipase displayed an *E*-value of 31, which resulted in the isolation of enantiomerically pure alcohol (*S*)-(+)-**3** (>99% ee) with 58% conv. after 24 h (Table 1, entry 5). The same results in terms of the enantiomeric excess for the slower-reacting enantiomer (*S*)-(+)-**3** were obtained with Novozym 435 and Amano PS-IM (Table 1, entries 2 and 4). However, both the afore-mentioned enzymes catalyzed KR of *rac*-**3** at a significantly lower rate reaching 55–67% conv. after 120 h. Accordingly, our attention was paid to Chirazyme L-2, C-2, and this biocatalyst was further used in the optimization procedure toward KR of *rac*-**3**. 

Since the enzymatic reaction rate, stereoselectivity, and thermal stability largely depend on the reaction medium [46,47,48], optimization studies were continued to evaluate the effect of the organic solvents on Chirazyme L-2, C-2-catalyzed enantioselective transesterification of *rac*-**3** using vinyl acetate (Table 2). 

In this regard, the EKR processes were carried out in organic solvents of varying polarity values within which the racemic substrate *rac*-**3** could form homogenous solutions at the same ratio of the reactants as in the previous step. However, to increase the economic viability of this reaction, a reduced amount (10% *w*/*w*) of the biocatalyst was employed. It is a well-known fact that decreasing the amount of biocatalyst can be responsible for a drop in the reaction rate. Indeed, such a phenomenon was observed in the case of Chirazyme L-2, C-2-catalyzed KR of *rac*-**3** in MTBE, which had to be elongated to 72 h to achieve a comparable 57% conv. While screening the acylation of *rac*-**3** with vinyl acetate in various co-solvent systems, we observed the following approximate rate trend: Et_2_O~MTBE > *n*-hexane > THF > CH_3_CN > 1,4-dioxane > *tert*-amyl alcohol (2-methyl-2-butanol) > acetone. Another trend order was observed for enantioselectivity: THF > MTBE ~ *tert*-amyl alcohol > Et_2_O > *n*-hexane > acetone > CH_3_CN > 1,4-dioxane. Considering the reaction rates and the values of the *E*-factor, it was clear that the most promising results were obtained when Chirazyme L-2, C-2 was suspended in THF or MTBE, respectively. Since MTBE is generally free from dangerous peroxides, it was chosen for further optimization studies due to safety regimes. 

The nature of an acyl transfer reagent is a crucial factor in the outcome of lipase-catalyzed acylation of secondary alcohols in organic solvents. This feature mainly stems from the fact that the type of acyl donor can markedly influence the selectivity and the rate of reactions performed with lipases [49,50,51,52,53]. Therefore, the effect of acyl donor in transesterification of *rac*-**3** was examined by carrying out the EKR processes in the presence of 2-fold molar excess of three different vinyl esters for 16 h at 25 °C (Table 3). In all cases, the enzyme amount was increased to 20% (*w*/*w*) in ratio to *rac*-**3**, which considerably enhanced the reaction rate, thus allowing to achieve similar conversions as in the previous step.

Undertaken comparative studies with the employed vinyl esters revealed that enantioselectivity of Chirazyme L-2, C-2-catalyzed transesterification of *rac*-**3** varied negligible (Δ*E* < 13). For comparable conversions (ca. 55%), the highest value of enantioselectivity factor (*E* = 59) was observed when vinyl butanoate was employed as the acyl donor (Table 3, entry 2). The barely marked superiority of vinyl butyrate excluded its use as an acyl donor in KR of *rac*-**3** since this reagent is relatively expensive compared to vinyl acetate. Moreover, vinyl butyrate is a significantly less volatile compound {bp 116 °C (760 Torr) [54]} than vinyl acetate {bp 73 °C (760 Torr) [55]}, and after completion of the enzymatic reaction, its excess usually hampers the purification process. The features mentioned above show that vinyl acetate seemed to be more suitable for KR of *rac*-**3**. In turn, it is noteworthy that when acyl donor was used in a lower molar excess than before (2 equiv instead of 16 equiv), the enantioselectivity factor (*E*) slightly increased from 35 to 49.

The integration of the results obtained from the optimization studies revealed that the most efficient biocatalyst was Chirazyme L-2, C-2 when used at 20 mg/mL conc. and suspended in a solution of *rac*-**3** (100 mg/mL conc.) in a mixture of vinyl acetate (2 equiv) and MTBE (1 mL/0.69 mmol of *rac*-**3**) as the co-solvent. In the next step, to show the robustness of the developed methodology, the biotransformation of *rac*-**3** was scaled up to multi-gram amounts of the racemic substrate (Table 4). 

At first, Chirazyme L-2, C-2-catalyzed transesterification of *rac*-**3** was carried out at 1 g-scale of the substrate. Termination of the EKR process after 16 h furnished slower-reacting enantiomer (*S*)-(+)-**3** in 34% yield with >99% ee and the formed optically active acetate (*R*)-(–)-**4a** in 40% yield with 75% ee and 57% conversion. This result showed that the linear enlargement of EKR parameters was prone to scalability. In turn, to obtain antipode (*R*)-(–)-**3** with higher enantiomeric purity, the preparative-scale EKR of *rac*-**3** was carried out using Amano PS-Immobead 150 as the biocatalysts, which showed ultra-selectivity toward the formation of homochiral (*R*)-(–)-**4a** from the racemic mixture. Arresting the reaction after 144 h (ca. 33% conv.) afforded (*S*)-(+)-**3** in 62% yield with 49% ee and (*R*)-(–)-**4a** in 28% yield with 99% ee, respectively. Next, optically active ester (*R*)-(–)-**4a** was hydrolyzed into the corresponding alcohol (*R*)-(–)-**3** isolated in 66% yield with 98% ee. In addition, the preparative EKR was performed using 10 g of *rac*-**3**, which resulted in the isolation of (*S*)-(+)-**3** in 36% yield with >99% ee and (*R*)-(–)-**4a** in 38% yield with 79% ee. In this case, to achieve comparable conversion (56%), the reaction had to be elongated to 27 h. A drop in the reaction rate might be attributed to mass transfer limitations, which constitute a typical drawback for the processes conducted in a batch mode. However, further optimization was discontinued as it was far beyond the scope of this study.

### 2.4. Determination of the Absolute Configuration of (S)-(+)-***3***

**Scheme 2:**
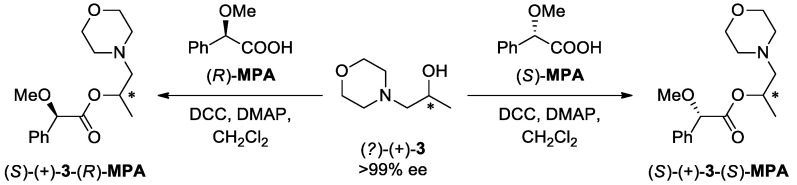
The assignment of the absolute configuration of alcohol (+)-**3** resulted from derivatization with (*R*)-**MPA** and (*S*)-**MPA**, respectively. Reagents and conditions: (*R*)- or (*S*)-**MPA** (1 equiv), DCC (1.2 equiv), DMAP (cat.), dry CH_2_Cl_2_, rt, 72 h. The asterix (*) states for asymmetric carbon atom with undefined absolute configuration.

Although the preparation of optically active alcohol (*S*)-(+)-**3** has already been reported via the reduction of the corresponding lactamide derived from ethyl (2*S*)-lactate with borane-methyl sulfide [56], clear proof of its absolute configuration has not been provided yet. Furthermore, the lack of literature data concerning a specific rotation sign of the enzymatic KR products prevented the unambiguous classification of the stereochemistry of non-racemic morpholino-alcohol derivatives. Therefore, the assignment of the absolute configuration of (*S*)-(+)-**3** was determined through a combination of synthetic transformations and spectroscopic studies by following the so-called Mosher’s methodology modified by Riguera et al. [57]. In this regard, enantiomerically pure alcohol of undefined stereochemistry (*?*)-(+)-**3** (>99% ee) was esterified in two separate experiments with both enantiomers of α-methoxy-α-phenylacetic acid (**MPA**) as the chiral derivatizing agents (CDAs) (Scheme 2). 

In practice, the double derivatization procedure employing CDAs assumes the formation of two diastereomers (in this case: (2*S*)-1-(morpholin-4-yl)propan-2-yl (2*R*)-methoxy(phenyl)acetate ((*S*)-(+)-**3**-(*R*)-**MPA**) and (2*S*)-1-(morpholin-4-yl)propan-2-yl (2*S*)-methoxy(phenyl)acetate ((*S*)-(+)-**3**-(*S*)-**MPA**) in which NMR chemical shifts of the protons present in functional groups directly attached to the stereogenic center of alcoholic (+)-**3** fragment should be different as a result of the shielding effect of the phenyl substituent of chiral CDA. This observation stems from the anisotropic effect of the π-electron-rich benzene ring. Another key feature of this experimental model is that the most stable conformer among the MPA esters of mono-functional secondary alcohols, by a value of 0.6–1.0 kcal/mol, is the *sp* conformer [58]. It means that the methoxy group, the Cα carbon, and the carbonyl group of the **MPA** unit, as well as the methine proton of the alcohol part, are in the same plane with the methoxy and the carbonyl groups in a *syn*-periplanar disposition. 

**Figure 4:**
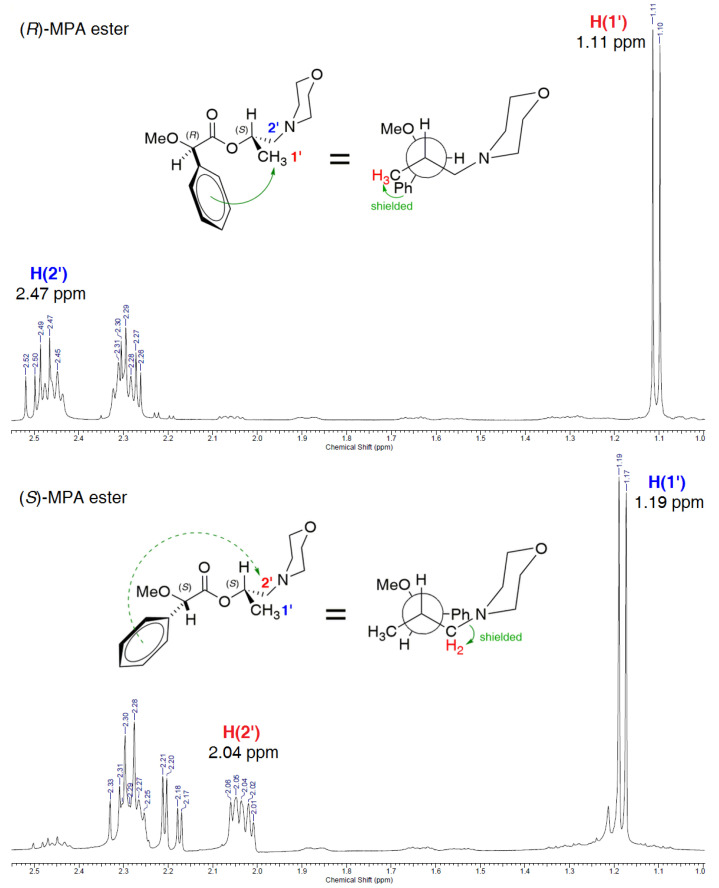
Partial ^1^H NMR (CDCl_3_, 400 MHz) spectra of diastereoisomers (*S*)-(+)-**3**-(*R*)-**MPA** (upper) and (*S*)-(+)-**3**-(*S*)-**MPA** (bottom) derived from enantiomerically pure alcohol (+)-**3** (>99% ee) of an unknown absolute configuration. With red color are marked protons of alcohol fragment (+)-**3** shielded by the phenyl substituent of chiral auxiliary **MPA**; blue labels stand for unaffected (deshielded) protons. Green arrows indicate the shielding effect caused by π-electron-rich aromatic system.

To determine the stereochemistry of a chiral carbon atom of (+)-**3**, sequential experimental-theoretical operations were made: (i) spectra for both **MPA**-esters (*S*)-(+)-**3**-(*R*)-**MPA** and (*S*)-(+)-**3**-(*S*)-**MPA** were recorded (Figure 4), (ii) proton NMR signals of both afore-mentioned species were assigned, (iii) the differences between the chemical shifts of the signals for a certain proton of the two synthesized derivatives (expressed as Δδ*^RS^*) were calculated.

The comparison of the spectra obtained from (*S*)-(+)-**3**-(*R*)-**MPA** and (*S*)-(+)-**3**-(*S*)-**MPA**, as well as considering a greater representative population of the *sp* conformer, led to the following observations: (i) the benzene ring of the chiral auxiliary projects its magnetic anisotropy more strongly toward methyl group protons H(1’) in the (*R*)-MPA derivative (*S*)-(+)-**3**-(*R*)-**MPA**, while the same group of protons in the corresponding (*S*)-MPA ester (*S*)-(+)-**3**-(*S*)-**MPA** remains unaffected; (ii) the opposite phenomenon is observed for methylene protons H(2’), which are shielded in (*S*)-(+)-**3**-(*S*)-**MPA** due to the direction of anisotropic effect of the phenyl moiety, while in (*S*)-(+)-**3**-(*R*)-**MPA** they are unaffected. 

**Figure 5:**
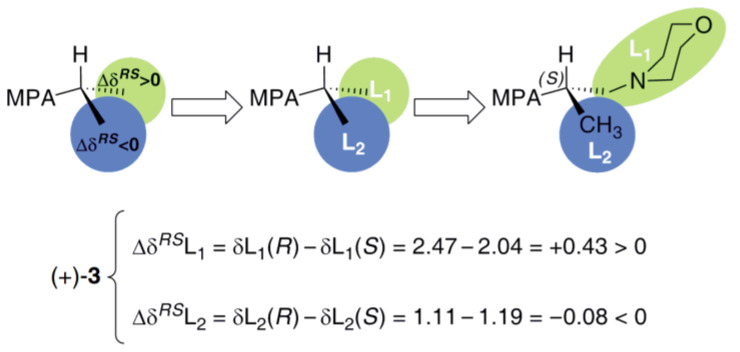
Description of the substituents (L_1_ and L_2_) for determination of the absolute configuration of (+)-**3** and Δδ*^RS^* values obtained for the MPA diastereomeric esters (*S*)-(+)-**3**-(*R*)-**MPA** and (*S*)-(+)-**3**-(*S*)-**MPA**.

From the findings mentioned above and the sign distributions of the equations presented in Figure 5, the (*S*)-configuration of the examined alcohol (+)-**3** is apparent. In this context, the results of NMR experiments proved that a simple and low-cost modified form of Mosher’s methodology could provide a reliable determination of the absolute configuration of non-racemic 1-(morpholin-4-yl)propan-2-ol (**3**). This indirect analytical tool is incredibly precious in the case of liquid or oil compounds, toward which the application of unambiguous absolute stereochemistry assignment methods, such as X-ray diffraction (XRD) analysis, are unavailable due to lack of the appropriate monocrystals.

### 2.5. Synthesis of Optically Active Ethereal Analog of iso-Moramide (S)-(+)-***10*** and (R)-(–)-***10***

Continuing the planned synthesis of potential analgesics, a commercially available diphenylacetic acid (**7**) was transformed into corresponding acid chloride **8** by means of 15 equiv of thionyl chloride (SOCl_2_) in boiling benzene. Subsequently, **8** was reacted with 3.1 equiv of pyrrolidine in 1,4-dioxane to afford the respective amide **10** in 82% yield after two steps. Next, the afore-prepared optically active morpholino-alcohols (*S*)-(+)-**3** (>99% ee) and (*R*)-(–)-**3** (98% ee) were subjected to chlorination using SOCl_2_ in chloroform (CHCl_3_). It is worth noting that this approach resulted in the formation of the respective 4-(2-chloropropyl)morpholine enantiomers (*R*)-(–)-**5** (>99% ee) and (*S*)-(+)-**5** (98% ee) in high 69–71% yield range, and with complete inversion of the stereochemical configuration, even though the reactions were carried out without additional hydrogen chloride acceptor (i.e., pyridine, triethylamine). The observed total stereoinversion might be attributed to the fact that the in situ formed HCl was effectively trapped by the nitrogen atom of the morpholine ring, thus preventing the racemization of the chiral alcohols (*S*)-(+)-**3** or (*R*)-(–)-**3** as a result of S*_N_*1-type reaction. In order to confirm the S*_N_*2 mechanism of the reaction with sole SOCl_2_, the additional (alternative) S*_N_*2-type chlorination of (*S*)-(+)-**3** using tetrachloromethane (CCl_4_) and triphenylphosphine (Ph_3_P) under Appel reaction conditions was performed. In this case, the desired chloride (*R*)-(–)-**5** was isolated in a 57% yield without racemization (>99% ee). The optical purity of both enantiomeric forms of chloro-derivative (*R*)-(–)-**5** and (*S*)-(+)-**5** was assessed with chiral HPLC after derivatization of the halogen moiety with 10 equiv of thiophenol (PhSH) under basic conditions (EtONa/EtOH) (for details, see Experimental Section 3.13). 

**Figure 6:**
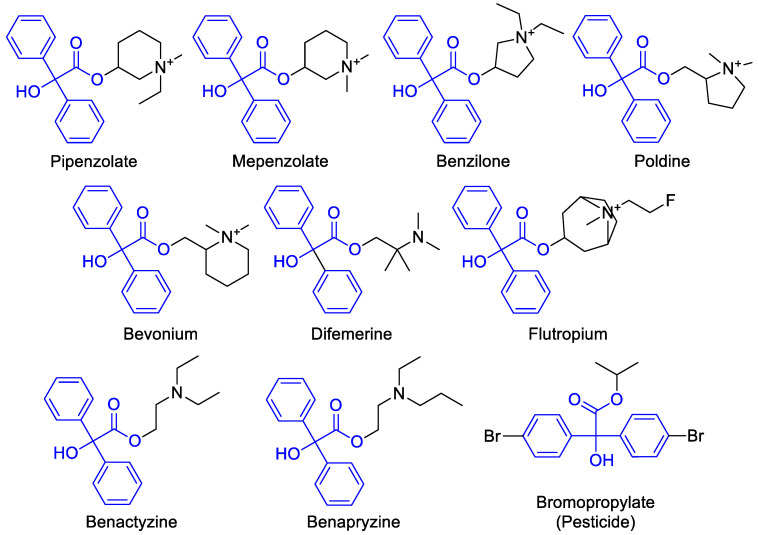
Representative examples of medicinal or agrochemical agents containing diphenylacetyl core structure.

With both enantiomers of 4-(2-chloropropyl)morpholine ((*R*)-(–)-**5** and (*S*)-(+)-**5**) in hand, we subsequently proceeded to the last step of the synthesis of target molecules (*S*)-(+)-**10** and (*R*)-(–)-**10**. In order to form an ethereal connection between *N*-diphenylacetyl-1-pyrrolidine (**9**) and 4-(2-chloropropyl)morpholine (**5**), and thus obtain desired chiral product **10**, at first, a tertiary α-hydroxycarbonyl compound had to be prepared from **9**. In general, a chemo- and regioselective α-hydroxylation of carbonyl compounds possessing diphenylacetyl moiety is a desirable synthetic task since many antimuscarinic and/or anticholinergic pharmaceuticals can be synthesized from that building block (Figure 6). 

Recently, two groundbreaking synthetic methods, including transition-metal-catalyzed C–H hydroxylation using a dinuclear Pd(II) complex as an oxygen transfer catalyst [59] and/or a transition-metal-free Cs_2_CO_3_/P(OEt)_3_/O_2_ reaction system [60], have been developed. Nevertheless, both these methods possess significant limitations. The first mentioned protocol employs an expensive and difficult-to-obtain Pd-catalyst, which complete removal from the product is a very laborious task, thus excluding it from industrial application, particularly drug synthesis. In turn, the second approach suffers from triethyl phosphite (P(OEt)_3_), a flammable and malodorous liquid that complicates the handling and utilization of contaminants. 

Therefore, we have explored an alternative procedure using a mixture of O_2_-saturated NaOH/DMSO in the presence of *N*-benzyl-*N*,*N*,*N*-triethylammonium chloride (TEBA(Cl)) as the phase transfer catalyst. Moreover, a huge 8-fold molar excess of NaOH with respect to carbonyl substrate **9** allowed the subsequent etherification of the in situ formed α-hydroxyl derivative with 1.3 equiv of (*R*)-(–)-**5** (>99% ee) under relatively mild reaction conditions. In this regard, a “one-pot” α-hydroxylation/etherification transformation of **9** was carried out at 40 °C for 4 h, furnishing (*R*)-(–)-**10** in 31% yield with 89% ee. After employing the same reaction conditions toward (*S*)-(–)-**5** (98% ee), the respective counterpart (*S*)-(+)-**10** was isolated in 20% yield with 87% ee. 

**Scheme 3:**
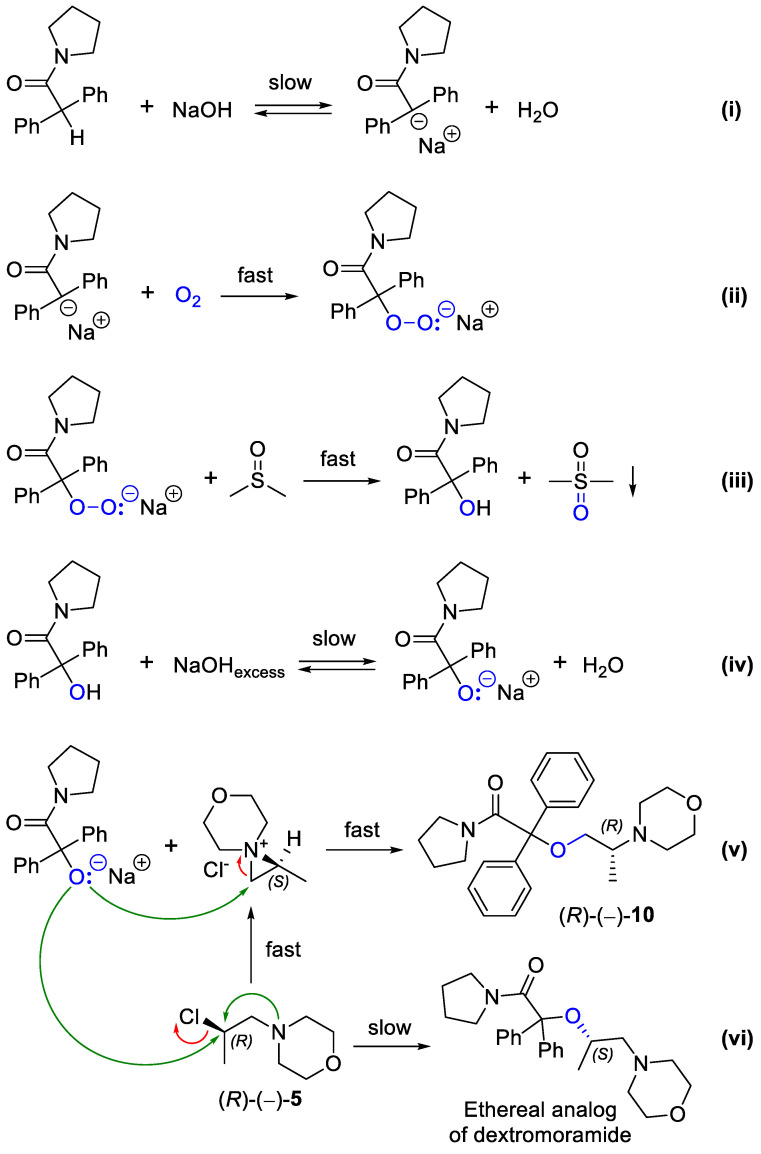
Proposed reaction mechanism for the formation of (*R*)-(–)-**10** from (*R*)-(–)-**5** mediated by O_2_-NaOH/DMSO/TEBA(Cl) catalytic system.

It seems rational that the studied reaction follows a base-catalyzed auto-oxidation mechanism proposed by Russell et al. [61,62]. Following this hypothesis, the overall α-hydroxylation/etherification process might be presented by the respective reaction sequence as depicted in Scheme 3. At first, (i) substrate **9** is deprotonated by sodium hydroxide/dimethyl sulfoxide (NaOH/DMSO) superbase, and (ii) the resulting carbanion is rapidly oxygenated by molecular oxygen (O_2_) as the oxidant to give the corresponding peroxide. In turn, the superoxide (iii) is further reduced by DMSO to the corresponding alcohol, followed by (iv and v) a regioselective ring-opening of the in situ formed aziridinium ion, leading to the formation of desired (*R*)-(–)-**10**. A relatively low reaction yield might be attributed to chemo- and regio-selectivity issues, resulting from a “double-reactivity” of the employed alkylating agent **5**. This phenomenon stems from a spontaneous intramolecular cyclization of (*R*)-(–)-**5** into a highly reactive aziridinium ion, which is most probably consumed in the unproductive side reactions. In this context, it appeared that the etherification reaction proceeds not only via a regioselective ring-opening of the aziridinium species but also (vi) through a direct nucleophilic displacement of the chloride ion present in *non*-racemic **5** as is observed in the case of convenient dextromoramide synthesis. Both these concurrent processes provide a complex mixture of isomeric products with reversed stereochemistry at the corresponding asymmetric carbon atoms, which complicate the isolation and purification of *non*-racemic derivative **10**. 

The purity and identity of isolated 2-[(2*R*)-2-(morpholin-4-yl)propoxy]-2,2-diphenyl-1-(pyrrolidin-1-yl)ethan-1-one ((*R*)-(–)-**10**) was confirmed by using complementary analytical methods including ^1^H and ^13^C NMR, DEPT-135, 2D NMR (HSQC), FT-IR, ESI-MS, GC-MS, and HPLC (for details, see the Supplementary Materials).

**Figure 7:**
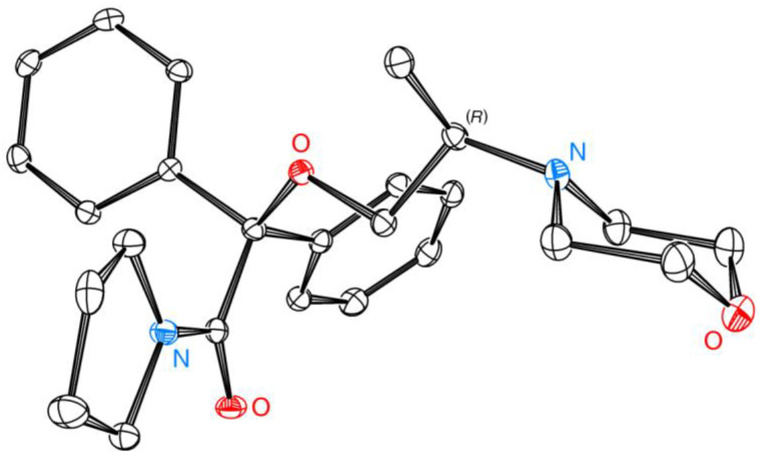
An ORTEP plot of 2-[(2*R*)-2-(morpholin-4-yl)propoxy]-2,2-diphenyl-1-(pyrrolidin-1-yl)ethan-1-one ((*R*)-(–)-**10**). The following crystal structure has been deposited at the Cambridge Crystallographic Data Centre and allocated the deposition number CCDC-2202212. Ellipsoids are set to 50% probability, and hydrogen atoms have been omitted for clarity. ORTEP plot was prepared in Ortep-3 for Windows (WinGX version 2021.3) software (Glasgow, Scotland) [63].

The chemical structure and absolute configuration of (*R*)-(–)-**10** were unambiguously confirmed by solving the crystal structure from monocrystals by using X-ray diffraction analysis (Figure 7). For details concerning the crystal growth conditions and routine crystal structure determination using XRD analysis, please see Experimental Section 3.18.

## 3. Materials and Methods

All commercially available reagents (purchased from Merck KGaA, (Darmstadt, Germany), Tokyo Chemical Industry Co., Ltd. (TCI) (Fukaya, Japan), Thermo Fisher (Kandel) GmbH (Kandel, Germany), and Fluorochem Ltd. (Hadfield Derbyshire, UK)) were used without further purification. Organic solvents (i.e., CH_2_Cl_2_, THF, DMSO) were dried by allowing them to stand over activated (oven-roasted under high-vacuum) 3Å molecular sieves (20% mass/volume (m/v) loading of the desiccant) at least for 48 h before use according to lit. [64]; DMSO was additionally passed through a column packed with activated SiO_2_ (25 g, Silica gel 60^®^ (0.015–0.040 mm), Merck KGaA, (Darmstadt, Germany)) before 3Å molecular sieves treatment; *n*-hexane, EtOAc, and acetone were purified by fractional distillation over an adequate desiccant under nitrogen before use; Et_2_O was at first distilled from H_2_SO_4_ and subsequently dried by dropping small pieces of sodium metal into a buttle containing this solvent. Chromatography grade *n*-hexane and isopropanol (2-PrOH) used in high performance liquid chromatography (HPLC) were purchased from Avantor Performance Materials Poland S.A. (formerly POCH Polish Chemicals Reagents). 

The enzyme preparations were purchased from Novozymes A/S (Bagsvaerd, Denmark), STREM Chemicals, Inc. (Newburyport, MA, USA), Amano Pharmaceutical Co., Ltd. (Nagoya, Japan), Sigma-Aldrich (currently Merck) (Darmstadt, Germany), Roche (Basel, Switzerland), Boehringer Mannheim (currently Roche Diagnostics) (Basel, Switzerland), and were used without pre-treatment (for details, see Table S1 appended in Supplementary Materials). 

Analytical scale enzymatic reactions were performed in thermo-stated glass vials (*V* = 4 mL) placed in Chemglass CG-1991-04 GOD Anodized Aluminum Reaction Block, 48 Position, 19 mm Hole Depth, For Circular Top Hot Plate Stirrer. All non-aqueous reactions were carried out under oxygen-free (argon-protective) conditions using over-dried glassware. 

Melting point (mp) ranges, uncorrected, were determined with a commercial apparatus (Thomas-Hoover “UNI-MELT” capillary melting point apparatus) on samples contained in rotating capillary glass tubes open on one side (1.35 mm inner diam. and 80 mm length). 

Analytical thin-layer chromatography was carried on TLC aluminum plates with silica gel Kieselgel 60 F_254_ (Merck, Germany) (0.2 mm thickness film containing a fluorescence indicator green 254 nm (F254) using vapours of iodide and/or UV light as a visualizing agent, respectively. 

Preparative separations were carried out by: (i) column chromatography using Merck silica gel 60 (230–400 mesh), with grain size 40–63 μm or by (ii) PLC PSC-Fertigplatten Kieselgel 60 F_254_ (20 × 20 cm with 2 mm thickness layer) glass plates purchased from Merck, (Darmstadt, Germany). 

The gas chromatography (GC) analyses were performed with an Agilent Technologies 6890N instrument (Santa Clara, CA, USA) equipped with a flame ionization detector (FID) and fitted with HP-50+ (30 m) semi-polar column (50% phenyl–50% methylpolysiloxane); the GC injector was maintained at 250 °C; Helium (2 mL/min) was used as carrier gas; retention times (*t*_R_) are given in minutes under these conditions; column temperature programs are given in Table S3 appended in Supplementary Materials.

The enantiomeric excesses (% ee) of optically active compounds were determined by high performance liquid chromatography (HPLC) analyses performed on Shimadzu CTO-10ASV chromatograph (Shimadzu Corporation, Japan) equipped with STD-20A UV detector and Chiralpak AD-H, Chiralcel OD-H or Chiralcel OJ-H (4.6 mm × 250 mm, coated on 5 µm silica gel grain size) chiral columns (Daicel Chemical Industries Ltd., Japan) equipped with dedicated pre-columns (4 mm × 10 mm, 5 µm) using mixtures of *n*-hexane/2-PrOH or *n*-hexane/*tert*-ButOH/Et_3_N as the respective mobile phases in the appropriate ratios; the HPLC analyses were executed in an isocratic and isothermal (30 °C) manner; flow (*f*) is given in mL/min; racemic compounds were used as standards; HPLC conditions and retention times (*t*_R_) are given in Table S4 appended in Supplementary Materials.

UV spectra were measured with Varian Cary 3 UV-Visible Spectrophotometer (Varian, Inc., Palo Alto, CA, USA). 

Optical rotations ([*α*]) were measured with a PolAAr 32 polarimeter in a 2 dm long cuvette using the sodium D line (589 nm) at 22 °C or 27.5 °C, respectively; [α]_D_ are given in units of: deg dm^−1^ cm^3^ g^−1^; the concentration *c* is in g/100 mL (for details, see Table S2 appended in Supplementary Materials). 

^1^H NMR and ^13^C NMR spectra were recorded on a Varian Mercury 400BB spectrometer (Varian, Inc., Palo Alto, CA, USA) operating at 400 MHz for ^1^H and 100 MHz for ^13^C nuclei (for titled product **10** ^1^H NMR (500 MHz), ^13^C NMR (126 MHz), ^13^C DEPT-135 NMR, and 2D NMR (HSQC) were recorded on Spektrometr Varian NMR System 500 MHz (Varian, Inc., USA)); chemical shifts (*δ*) are given in parts per million (ppm) on the delta scale related to the solvent peak used as reference value; signal multiplicity assignment: s, singlet; d, doublet; t, triplet; q, quartet; m, multiplet; coupling constant (*J*) are given in hertz (Hz); All samples were recorded as solutions in fully deuterated chloroform (CDCl_3_), methanol (CD_3_OD) or acetonitrile (CD_3_CN), respectively. All NMR reports for Supplementary Materials document were created by ACD/NMR Processor Academic Edition 12.0. (Freeware software provided by ACD/Labs (Advanced Chemistry Development, Inc. Toronto, Canada) and show only the delta range where signals were present. 

Mass spectrometry (MS) was recorded on a Micro-mass ESI Q-TOF spectrometer with an ESI ion source (70 eV ionization) and a linear ion trap analyzer; all samples were prepared by dilution of MeOH (0.5 mL) and additives of mixtures of CH_3_CN/MeOH/H_2_O (50:25:25, *v*/*v*/*v*) + 0.5% formic acid (HCO_2_H) each. 

GC-MS analysis was performed using an Agilent HP-6890 N gas chromatography apparatus coupled to a 5973 N quadrupole mass selective detector (Agilent Technologies, Palo Alto, CA, USA); chromatographic separations were carried out on non-polar RESTEK Rxi-1ms fused-silica capillary column (30 m × 0.25 mm) coated with 100% dimethylpolysiloxane (film thickness 0.25 mm) as a stationary phase; helium (purity 99.999%) was employed as carrier gas at a constant column flow-rate of 1.0 mL min^–1^; Injection mode: spitless at a temperature of 250 °C; column temperature program: sample was injected at an initial temperature of 100 °C, held for 1 min; ramped at 10 °C min^–1^ up to 320 °C, and held for 1 h; the mass spectrometer was operated in electron ionization mode (EI) at 70 eV, and a mass scan range from *m*/*z* = 40 to 400; ion source 280 °C; ion source vacuum 10^–5^ Torr. 

Fourier Transform Infrared spectra (FT-IR) spectra of neat samples were recorded on a Perkin Elmer System 2000 FTIR Spectrometer (PerkinElmer, Inc. Waltham, MA, USA) equipped with a Pike Technologies GladiATR attenuated total reflectance (ATR) accessory with a monolithic diamond crystal stage and a pressure clamp; FT-IR spectra were recorded in transmittance mode in the 300–4000 cm^–1^ range, in ambient air at room temperature, with 2 cm^–1^ resolution, 0.5 cm^–1^ interval and accumulation of 32 scans; the unit is given in %T. 

Molecular docking studies to establish favorable ligand binding geometries for the studied opioid analgesics and their affinity toward opioid receptors were carried out on a four CPUs-based desktop PC-computer equipped with AMD Phenom™ II X4 965 Processor 3.40 GHz and 32 GB of RAM on a Microsoft Windows 10 Professional 64-bit operating system (for details, Section 3.1).

X-ray diffraction (XRD) analysis of the selected crystals were measured with mirror monochromated CuKα radiation on an Oxford Diffraction κ-CCD Gemini A Ultra diffractometer (for details, see Section 3.18).

### 3.1. Molecular Docking

Molecular docking calculations were performed with AutoDock Vina v. 1.1.2. program (https://vina.scripps.edu/, accessed on 5 September 2022) [43] using the standard docking protocol described in our recent studies [65,66]. All ligands were prepared with ChemAxon MarvinSketch v. 14.9.1.0 (https://chemaxon.com/marvin, accessed on 5 September 2022), optimized in terms of geometry in Avogadro v. 1.2.0. (https://avogadro.cc/, accessed on 5 September 2022), and saved as .mol2 files. Macromolecule target crystal structures, including *µ*-type opioid receptor (*µ*-OR; PDB ID: 4DKL [40], 2.80 Å resolution), *δ*-type opioid receptor (*δ*-OR; PDB ID: 4EJ4 [41], 3.40 Å resolution), and *κ*-type opioid receptor (*κ*-OR; PDB ID: 4DJH [42], 2.90 Å resolution), were taken from the RCSB Protein Data Bank (https://www.rcsb.org/, accessed on 5 September 2022). All non-protein molecules (i.e., ligands and crystal waters) were removed, the polar hydrogens were then added, and Gesteiger charges were calculated using AutoDock tools v. 1.5.6. to get the appropriate file in .pdbqt format. Next, AutoGrid was used to find an appropriate grid box size in terms of *x*, *y*, *z* coordinates with the final size space dimension set as follows: *x* = 60 Å, *y* = 60 Å, *z* = 60 Å, and a grid spacing of 0.375 Å. Dockings were performed with an exhaustiveness level of 48 concerning global search. For each ligand molecule, 100 independent runs were performed using the Lamarckian Genetic Algorithm (GA) with at most 106 energy evaluations and a maximum number of generations of >27,000 Å^3^ (the search space volume). The rest of the docking parameters, including the remaining Lamarckian GA parameters, were set as default using the standard values for genetic Vina algorithms (the posed dockings were below 5.00 Å rmsd). The docking modes of each ligand were clustered and ranked based on a mutual ligand-protein affinity expressed as absolute free binding energies (Δ*G*_calc_ (kcal/mol)) as well as the rmsd-values in both modes regarding rmsd lower bound (l.b.) and rmsd upper bound (u.b.), respectively. The receptor-ligand interactions were visualized using PyMOL Molecular Graphics System software v. 1.3, Schrödinger, LLC (https://pymol.org/2/, accessed on 5 September 2022). The validation of the docking protocol was achieved by ensuring that the database ligands (i.e., methyl 4-{[(5β,6α)-17-(cyclopropylmethyl)-3,14-dihydroxy-4,5-epoxymorphinan-6-yl]amino}-4-oxobutanoate (BFO, in the case of 4DKL), (4b*S*,8*R*,8a*S*,14b*R*)-7-(cyclopropylmethyl)-5,6,7,8,14,14b-hexahydro-4,8-methano [1]benzofuro [2,3-a]pyrido [4,3-b]carbazole-1,8a(9*H*)-diol (naltrindole, in the case of 4EJ4), and (3*R*)-7-hydroxy-*N*-{(2*S*)-1-[(3*R*,4*R*)-4-(3-hydroxyphenyl)-3,4-dimethylpiperidin-1-yl]-3-methylbutan-2-yl}-1,2,3,4-tetrahydroisoquinoline-3-carboxamide (JDTic, in the case of 4DJH)) could be re-docked to the respective OR under the established parameters, resulting in the same accommodation as in the co-crystallized complexes. For docking scoring, see Table S5 appended in Supplementary Materials.

### 3.2. General Procedure for the Synthesis of 1-(Morpholin-4-yl)propan-2-ol rac-***3***

To a solution of propylene oxide (*rac*-**2**, 20 g, 0.34 mol) in H_2_O (160 mL), morpholine (**1**, 36 g, 0.41 mol) was added in one portion at the temperature of NaCl-ice-bath (0–5 °C), and then warmed slowly to room temperature (ca. 25 °C) under vigorous stirring for 24 h. Next, H_2_O (100 mL) was added, and the aqueous mixture was extracted with Et_2_O (3 × 200 mL) and next EtOAc (2 × 200 mL), respectively. The collected organic phase was dried over anhydrous Na_2_SO_4_, and after filtering off the drying agent, the solvents were removed under reduced pressure to give the crude reaction mixture, which was purified by short-pad column chromatography on silica gel using a mixture of CHCl_3_/MeOH (80:20, *v*/*v*) as an eluent yielding desired *rac*-**3** (34.2 g, 68% yield) as a colorless liquid. *R*_f_ (CHCl_3_/MeOH; 80:20, *v*/*v*) 0.82 or *R*_f_ (CHCl_3_/MeOH; 95:5, *v*/*v*) 0.38; ^1^H NMR (CDCl_3_, 400 MHz) *δ*: 1.08 (d, *J* = 6.2 Hz, 3H, C*H*_3_), 2.14–2.41 (m, 4H, C*H*_2_N(CH_2_)C*H*_2_), 2.54–2.68 (m, 2H, NC*H*_2_CH), 3.40 (s, 1H, O*H*), 3.60–3.72 (m, 4H, C*H*_2_OC*H*_2_), 3.75–3.85 (m, 1H, C*H*); ^13^C NMR (CDCl_3_, 100 MHz) *δ*: 19.9 (*C*H_3_), 53.5 (*C*H_2_N(CH_2_)*C*H_2_), 61.9 (N*C*H_2_CH), 66.0 (*C*H), 66.8 (*C*H_2_O*C*H_2_); MS (ESI) *m*/*z* [M+H]^+^ calcd for C_7_H_16_NO_2_^+^ 146.1181, found 146.1169; FT-IR (neat) *ν*_max_ (cm^−1^): 3434, 2964, 2932, 2856, 2810, 1735, 1455, 1374, 1324, 1295, 1271, 1241, 1208, 1140, 1114, 1069, 1011, 941, 910, 864, 844, 798, 752, 631, 495; U*V*/*V*IS: λ_max_ = 208 nm (EtOH); GC (80–260 (10 °C/min)): *t_R_* = 4.43 min or (120–260 (10 °C/min)): *t_R_* = 2.20 min; HPLC (*n*-hexane/EtOH (90:10, *v*/*v*); f = 0.6 mL/min; λ = 208 nm; *T* = 30 °C, Chiralpak AD-H): *t_R_ =* 14.611 min (*R*-isomer) and 16.213 min (*S*-isomer).

### 3.3. General Procedure for the Synthesis of 1-(Morpholin-4-yl)propan-2-yl acetate rac-***4a***

The solution of racemic 1-(morpholin-4-yl)propan-2-ol (*rac*-**3**, 1 g, 6.89 mmol) in dry CH_2_Cl_2_ (15 mL) was cooled to 0–5 °C and acetic anhydride (Ac_2_O, 844 mg, 8.26 mmol), Et_3_N (2.09 g, 20.66 mmol, 2.5 mL) and DMAP (15 mg) were added. The reaction mixture was stirred at room temperature (ca. 25 °C) overnight, then quenched with H_2_O (15 mL), and then with saturated NaHCO_3_ solution (15 mL). The combined aqueous phase was extracted with Et_2_O (3 × 15 mL). The organic layer was additionally washed with brine (25 mL) and dried over anhydrous Na_2_SO_4_. After filtering off the drying agent and the solvent evaporation under vacuum, the crude product was purified by column chromatography on silica gel using a gradient of CHCl_3_/MeOH (95:5, 90:10, 80:20, *v*/*v*) mixture as an eluent yielding *rac*-**4a** (1.06 g, 82% yield) as yellowish oil. *R*_f_ (CHCl_3_/MeOH 95:5, *v*/*v*) 0.64; ^1^H NMR (CDCl_3_, 400 MHz) *δ*: 1.20 (d, *J* = 6.3 Hz, 3H, C*H*_3_CH), 2.01 (s, 3H, C*H*_3_C=O), 2.25–2.55 (m, 6H, C*H*_2_N(CH_2_)C*H*_2_ and NC*H*_2_CH), 3.64 (t, *J* = 4.6 Hz, 4H, C*H*_2_OC*H*_2_), 5.01–5.12 (m, 1 H, C*H*); ^13^C NMR (CDCl_3_, 100 MHz) *δ*: 18.4 (*C*H_3_CH), 21.3 (*C*H_3_C=O), 53.9 (2C, *C*H_2_N(CH_2_)*C*H_2_), 63.3 (*C*H), 66.9 (2C, *C*H_2_O*C*H_2_), 67.4 (N*C*H_2_CH), 170.5 (C=O); MS (ESI) *m*/*z* [M+H]^+^ calcd for C_9_H_18_NO_3_^+^ 188.1287, found 188.0999; FT-IR (neat) *ν*_max_ (cm^−1^): 2961, 2935, 2856, 2809, 1732, 1455, 1371, 1297, 1276, 1236, 1154, 1116, 1060, 1013, 957, 932, 901, 864, 827, 797, 630, 607, 484; U*V*/*V*IS: λ_max_ = 208 nm (EtOH); GC (80–260 (10 °C/min)): *t_R_* = 6.39 min or (120–260 (10 °C/min)): *t_R_* = 3.28 min.

### 3.4. General Procedure for the Synthesis of 1-(Morpholin-4-yl)propan-2-yl butanoate rac-***4b***

To a solution of racemic 1-(morpholin-4-yl)propan-2-ol (*rac*-**3**, 1 g, 6.89 mmol) in dry CH_2_Cl_2_ (15 mL), Et_3_N (767 mg, 7.58 mmol, 0.92 mL), butanoyl chloride (807 mg, 7.58 mmol) and DMAP (20 mg, 0.16 mmol) were added. The reaction mixture was stirred at room temperature (ca. 25 °C) overnight, and then the content of the flask was quenched with H_2_O (15 mL) and then with saturated NaHCO_3_ solution (15 mL). The combined aqueous phase was extracted with Et_2_O (15 mL) and CH_2_Cl_2_ (2 × 15 mL), respectively. The combined organic extracts were washed with a saturated solution of NaHCO_3_ (15 mL) and brine (15 mL). After drying the organic phase over Na_2_SO_4_, filtration of the drying agent under vacuum, and solvent evaporation on a rotatory evaporator, the crude product was purified by column chromatography on silica gel using sole EtOAc (100%) as the eluent, thus yielding *rac*-**4b** (620 mg, 42% yield) as yellowish oil. *R*_f_ (EtOAc, 100%) 0.56; ^1^H NMR (CDCl_3_, 400 MHz) *δ*: 0.93 (t, *J* = 8.0 Hz, 3H, C*H*_3_CH_2_), 1.19 (d, *J* = 8.0 Hz, 3H, C*H*_3_CH), 1.62 (sxt, *J* = 8.0 Hz, 2H, CH_3_C*H*_2_), 2.20–2.26 (m, 2H, C*H*_2_C=O), 2.27–2.55 (m, 6H, C*H*_2_N(CH_2_)C*H*_2_ and NC*H*_2_CH), 3.63 (t, *J* = 4.6 Hz, 4H, C*H*_2_OC*H*_2_), 5.03–5.14 (m, 1H, C*H*); ^13^C NMR (CDCl_3_, 100 MHz) *δ*: 13.6 (*C*H_3_CH_2_), 18.4 (CH_3_*C*H_2_), 18.5 (*C*H_3_CH), 36.5 (*C*H_2_C=O), 53.9 (2C, *C*H_2_N(CH_2_)*C*H_2_), 63.4 (C*H*), 66.9 (2C, *C*H_2_O*C*H_2_), 67.0 (N*C*H_2_CH), 173.1 (C=O); FT-IR (neat) *ν*_max_ (cm^−1^): 2963, 2935, 2854, 2812, 1730, 1455, 1377, 1296, 1276, 1252, 1183, 1117, 1059, 1014, 952, 933, 904, 864, 798, 631, 484; U*V*/*V*IS: λ_max_ = 202 nm (EtOH); MS (ESI) *m*/*z* [M+H]^+^ calcd for C_11_H_22_NO_3_^+^ 216.1600, found 216.1278; GC (120–260 (10 °C/min)): *t_R_* = 4.98 min.

### 3.5. General Procedure for the Synthesis of 1-(Morpholin-4-yl)propan-2-yl decanoate rac-***4c***

To a solution of racemic 1-(morpholin-4-yl)propan-2-ol (*rac*-**3**, 1 g, 3.89 mmol) in dry CH_2_Cl_2_ (15 mL), Et_3_N (433 mg, 4.27 mmol, 0.52 mL), decanoyl chloride (815 mg, 4.27 mmol) and DMAP (20 mg, 0.16 mmol) were added. The combined aqueous phase was extracted with Et_2_O (15 mL) and CH_2_Cl_2_ (2 × 15 mL), respectively. The combined organic extracts were washed with a saturated solution of NaHCO_3_ (15 mL), brine (15 mL), and dried over Na_2_SO_4_. After filtration and solvent evaporation under reduced pressure, the crude product was purified by column chromatography on silica gel using sole EtOAc (100%) as the eluent to afford *rac*-**4c** (731 mg, 63% yield) as yellowish oil. *R*_f_ (EtOAc, 100%) 0.69; ^1^H NMR (CDCl_3_, 400 MHz) *δ*: 0.84 (t, *J* = 7.0 Hz, 3H, C*H*_3_CH_2_), 1.18 (d, *J* = 6.5 Hz, 3H, C*H*_3_CH), 1.20–1.34 (m, 12H, C*H*_2_), 1.57–1.67 (m, 2H, C*H*_2_CH_2_C=O), 2.20–2.55 (m, 8H, C*H*_2_N(CH_2_)C*H*_2_ and NC*H*_2_CH and C*H*_2_C=O), 3.63 (t, *J* = 4.63 Hz, 4H, C*H*_2_OC*H*_2_), 5.03–5.15 (m, 1H, C*H*); ^13^C NMR (CDCl_3_, 100 MHz) *δ*: 14.0 (*C*H_3_CH_2_), 18.4 (*C*H_3_CH), 22.6 (*C*H_2_), 25.0 (*C*H_2_CH_2_C=O), 29.0 (*C*H_2_), 29.2 (*C*H_2_), 29.2 (*C*H_2_), 29.4 (*C*H_2_), 31.8 (*C*H_2_), 34.6 (*C*H_2_C=O), 53.9 (2C, *C*H_2_N(CH_2_)*C*H_2_), 63.4 (*C*H), 66.9 (2C, *C*H_2_O*C*H_2_), 67.0 (N*C*H_2_CH), 173.2 (C=O); MS (ESI) *m*/*z* [M+H]^+^ calcd for C_17_H_34_NO_3_^+^ 300.2539, found 300.2323; FT-IR (neat) *ν*_max_ (cm^−1^): 2956, 2924, 2854, 1735, 1455, 1377, 1296, 1276, 1246, 1173, 1118, 1062, 1014, 902, 865, 798, 722, 631, 481; U*V*/*V*IS: λ_max_ = 202 nm (EtOH); GC (120–260 (10 °C/min)): *t_R_* = 11.15 min.

### 3.6. General Procedure for Kinetic Resolution of rac-***3***–Enzyme Screening

To the solution of racemic 1-(morpholin-4-yl)propan-2-ol (*rac*-**3**, 100 mg, 0.69 mmol) in MTBE (1 mL) the suspension of the appropriate lipase (20 mg, 20% *w*/*w* (catalyst/substrate *rac*-**3**)) in vinyl acetate (948 mg, 11 mmol, 1 mL) and added in one portion. The reaction mixture was stirred (500 rpm, IKA RCT basic) in a thermo-stated glass vial (*V* = 4 mL) at 25 °C. Aliquots were regularly checked by gas chromatography (GC), and after the achievement of the required conversion, the enzyme preparation was removed by filtration and washed with portions of MTBE (10 mL) and MeOH (5 mL), respectively. The excess vinyl acetate and the volatile solvents were evaporated under reduced pressure. The crude residue was purified by column chromatography on silica gel using a mixture of CHCl_3_/MeOH (95:5, *v*/*v*) as an eluent to afford enantiomerically enriched alcohol (*S*)-(+)-**3** and acetate (*R*)-(–)-**4a**, respectively. To determine the ee-value of the ester, (*R*)-(–)-**4a** was subjected to basic methanolysis in the manner described below (for details, see Section 3.11). For HPLC analysis, the samples were prepared by dilution with *n*-hexane-2-PrOH (1.5 mL, 3:1, *v*/*v*) and filtered before injection. For additional data, see Table 1.

### 3.7. General Procedure for Kinetic Resolution of rac-***3***–Solvent Screening

The reaction mixture containing *rac*-**3** (100 mg, 0.69 mmol), Chirazyme L-2, C-2 (10 mg, 10% *w*/*w* (catalyst/substrate *rac*-**3**)), vinyl acetate (948 mg, 11 mmol, 1 mL), and the appropriate organic solvent (1 mL) was stirred (500 rpm, IKA RCT basic) in thermo-stated glass vial (*V* = 4 mL) at 25 °C. The rest of the procedure was essentially the same as in the previous section (for details, see Section 3.6). For details, see Table 2. 

### 3.8. General Procedure for Kinetic Resolution of rac-***3***–Acyl Donor Screening

The reaction mixture containing *rac*-**3** (100 mg, 0.69 mmol), Chirazyme L-2, C-2 (20 mg, 20% *w*/*w* (catalyst/substrate *rac*-**3**)), MTBE (1 mL), and the respective acyl donor (1.38 mmol, 2 equiv) (i.e., vinyl acetate (119 mg, 127 μL) or vinyl butanoate (157 mg, 169 μL) or vinyl decanoate (273 mg, 293 μL)) was stirred (500 rpm, IKA RCT basic) in thermo-stated glass vial (*V* = 4 mL) at 25 °C. The rest of the procedure was essentially the same as in the previous sections (for details, see Section 3.6). For details, see Table 3.

### 3.9. General Procedure for Gram-Scale Kinetic Resolution of rac-***3***

Racemic alcohol *rac*-**3** (1 g, 6.88 mmol) was dissolved in MTBE (10 mL). Afterward, the suspension of the corresponding lipase (200 mg, 20% *w*/*w* (catalyst/substrate *rac*-**3**)) in vinyl acetate (1.19 g, 13.77 mol, 1.27 mL) was added in one portion. The reaction mixture was stirred (500 rpm, IKA RCT basic) in a round-bottomed flask (*V* = 25 mL) at 25 °C for 16 h (in the case of Chirazyme L-2, C-2) and for 144 h (in the case of Amano PS-Immobead 150). Next, the biocatalyst was removed by filtration, and the filtrate cake was washed with MTBE (20 mL) and MeOH (10 mL), respectively. The volatile compounds were evaporated from the permeate under reduced pressure, and the residue was purified by column chromatography on SiO_2_ using mixture of CHCl_3_/MeOH (95:5, *v*/*v*) to afford enantiomerically enriched (*S*)-(+)-1-(morpholin-4-yl)propan-2-ol ((*S*)-(+)-**3**, 340 mg, 34% yield, >99% ee, [α]_D_^27.5^ = +50.40 (*c* 1.3, CHCl_3_) in the case of Chirazyme L-2, C-2 or 623 mg, 62% yield, 49% ee in the case of Amano PS-Immobead 150) and (*R*)-(–)-1-(morpholin-4-yl)propan-2-yl acetate ((*R*)-(–)-**4a**, 521 mg, 40% yield, 75% ee in the case of Chirazyme L-2, C-2 or 364 mg, 28% yield, 99% ee, [α]_D_^27.5^ = –4.66 (*c* 1.2, CHCl_3_) in the case of Amano PS-Immobead 150). The optically active ester (*R*)-(–)-**4a** was hydrolyzed by means of NaOH (1.1 equiv) in MeOH (3.5 mL) to give corresponding alcohol (*R*)-(–)-**3** (for details, see Section 3.11). The details concerning the results of preparative-scale EKR of *rac*-**3** are collected in Table 4. The physical, spectroscopic, and analytical data are identical as for the racemic standard compounds. 

### 3.10. General Procedure for Multigram-Scale Kinetic Resolution of rac-***3***

Racemic 1-(morpholin-4-yl)propan-2-ol (*rac*-**3**, 10 g, 68.9 mmol) was dissolved in MTBE (100 mL). Next, the suspension of Chirazyme L-2, C-2 [2 g, 20% *w*/*w* (catalyst/substrate *rac*-**3**)] in vinyl acetate (11.89 g, 1.38 mol, 12.72 mL) was added in one portion. The resulting mixture was stirred (500 rpm, IKA RCT basic) in a round-bottomed flask (500 mL) equipped with a magnetic stir bar (2 cm × 5 mm, 2 g) for 27 h at 25 °C. After enzyme removal and its subsequent washing with portions of MTBE (150 mL) and MeOH (50 mL), the filtrate was evaporated to dryness, and the remaining oil was subjected to column chromatography (20 g of SiO_2_ was applied for 1 g of the crude reaction mixture) using gradient of CHCl_3_/MeOH (95:5, 90:10, *v*/*v*) mixture as an eluent to yield enantiomerically enriched alcohol (*S*)-(+)-**3** (3.64 g, 36% yield, >99% ee, [α]_D_^27.5^ = +67.38 (*c* 1.18, CHCl_3_)) and acetate (*R*)-(–)-**4a** (4.85 g, 85% yield, 79% ee). The optically active ester (*R*)-(–)-**4a** was hydrolyzed by means of NaOH (1.1 equiv) in MeOH (3.5 mL) to give corresponding alcohol (*R*)-(–)-**3** (for details, see Section 3.11). The details concerning the results of preparative-scale EKR of *rac*-**3** are collected in Table 4. The physical, spectroscopic, and analytical data are identical to the standard racemic compounds.

### 3.11. General Procedure for Base-Mediated Methanolysis of (R)-(-)-1-(Morpholin-4-yl)propan-2-yl acetate (R)-(–)-***4a***

A solution of enantiomerically enriched acetate (*R*)-(–)-**4a** (3 g, 16 mmol, 99% ee) dissolved in MeOH (25 mL) was treated with a solution of NaOH (705 mg, 17.6 mmol) in MeOH (75 mL). The resulting mixture was stirred at room temperature (ca. 25 °C) for 30 min until complete consumption of the starting material (according to TLC). Then, the organic solvent was evaporated under reduced pressure, and the resulting solution was suspended in H_2_O (100 mL). The aqueous solution was subsequently extracted with CH_2_Cl_2_ (3 × 150 mL), Et_2_O (3 × 100 mL), and EtOAc (1 × 100 mL). The organic layers were combined, partially condensed on a rotary evaporator, and dried over anhydrous Mg_2_SO_4_. After filtering off the drying agent and solvent evaporation in vacuo, the crude reaction mixture was purified by column chromatography on silica gel eluting with a gradient of CHCl_3_/MeOH (95:5, 90:10, 80:20, *v*/*v*) mixture to yield (*R*)-(–)-**3** (1.54 g; 66% yield; 98% ee, [α]_D_^22^ = –73.03 (*c* 1.8, CHCl_3_)). 

### 3.12. General Procedure for the Determination of the Absolute Configuration of (S)-(+)-1-(Morpholin-4-yl)propan-2-ol (S)-(+)-***3*** Realized Via Esterification of (S)-(+)-***3*** with Enantiomers of α-Methoxy-α-phenylacetic Acid (R)-**MPA** or (S)-**MPA**

A catalytic amount of DMAP (5 mg) was added to a solution of enantiopure (*S*)-(+)-1-(morpholin-4-yl)propan-2-ol ((*S*)-(+)-**3**, 87 mg, 0.60 mmol, >99% ee), (*R*)- or (*S*)-α-methoxy-α-phenylacetic acid (100 mg, 0.60 mmol) as appropriate, and *N*,*N*’-dicyclohexylcarbodiimide (DCC, 148 mg, 0.72 mmol) in anhydrous CH_2_Cl_2_ (4 mL). After 72 h of stirring the reaction mixture at room temperature (ca. 25 °C), precipitated dicyclohexylurea was removed by filtration, and then the urea cake was rinsed with PhCH_3_ (3 × 10 mL). The combined organic solutions were washed with cold 1M HCl (2 × 10 mL), saturated NaHCO_3_ (2 × 10 mL), and brine (1 × 10 mL). Next, the organic layer was dried over MgSO_4_, filtered, evaporated to dryness, and the crude product as a yellow oil was diluted with a mixture of CHCl_3_/MeOH (2 mL, 1:1, *v*/*v*) and purified by preparative thin-layer chromatography using a mixture of CHCl_3_/MeOH (95:5, *v*/*v*) as an eluent. Appropriately, the separated fraction was removed from the glass plate with SiO_2_ gel, the silica matrix was ground, and the powder was placed in a round-bottomed flask and stirred with CHCl_3_/MeOH (100 mL, 1:1, *v*/*v*) for over 1 h. Finally, silica gel was filtered off, rinsed with MeOH (2 × 50 mL), and the resulting filtrate was evaporated to dryness to afford esters of (*S*)-(+)-**3** with (*R*)-**MPA** and (*S*)-**MPA**, respectively.

(2*S*)-1-(Morpholin-4-yl)propan-2-yl (2*R*)-methoxy(phenyl)acetate (ester of (*S*)-(+)-**3** and (*R*)-**MPA**, (*S*)-(+)-**3**-(*R*)-**MPA**). Yellowish semisolid; 85% yield; *R*_f_ (CHCl_3_/MeOH 95:5, *v*/*v*) 0.82; ^1^H NMR (CDCl_3_, 400 MHz) *δ*: 1.11 (d, *J* = 6.3 Hz, 3H, C*H*_3_), 2.24–2.34 (m, 3H, C*H*_2_N(CH_2_)C*H*_2_), 2.40–2.53 (m, 3H, NC*H*_2_CH and partially C*H*_2_N(CH_2_)C*H*_2_), 3.41 (s, 3H, OC*H*_3_), 3.54 (t, *J* = 4.6 Hz, 4H, C*H*_2_OC*H*_2_), 4.73 (s, 1H, C*H*C=O), 5.09–5.21 (m, 1H, CH_3_C*H*(O)CH_2_), 7.26–7.35 (m, 3H, Ph*H*), 7.40–7.46 (m, 2H, Ph*H*); ^13^C NMR (CDCl_3_, 100 MHz) *δ*: 17.9 (*C*H_3_), 53.8 (2C, *C*H_2_N(CH_2_)*C*H_2_), 57.3 (O*C*H_3_), 63.2 (CH_3_*C*H(O)CH_2_), 66.8 (2C, *C*H_2_O*C*H_2_), 68.2 (N*C*H_2_CH), 82.8 (*C*HC=O), 127.0 (2C, *o*-Ph), 128.3 (2C, *m*-Ph), 128.4 (*p*-Ph), 136.2 (*Ph*CH), 170.1 (C=O); MS (ESI) *m*/*z* [M+H]^+^ calcd for C_16_H_24_NO_4_^+^ 294.1705, found 294.1561. 

(2*S*)-1-(Morpholin-4-yl)propan-2-yl (2*S*)-methoxy(phenyl)acetate (ester of (*S*)-(+)-**3** and (*S*)-**MPA**, (*S*)-(+)-**3**-(*S*)-**MPA**). Yellowish semisolid; 94% yield; *R*_f_ (CHCl_3_/MeOH 95:5, *v*/*v*) 0.82; ^1^H NMR (CDCl_3_, 400 MHz) *δ*: 1.18 (d, *J* = 6.6 Hz, 3H, C*H*_3_), 1.99–2.09 (m, 2H, NC*H*_2_CH), 2.16–2.36 (m, 4H, C*H*_2_N(CH_2_)C*H*_2_), 3.33–3.43 (m, 7H, OC*H*_3_ and C*H*_2_OC*H*_2_), 4.69 (s, 1H, C*H*C=O), 5.12 (ddd, *J* = 8.5, 6.3, 3.5 Hz, 1H, CH_3_C*H*(O)CH_2_), 7.23–7.35 (m, 3H, Ph*H*), 7.40–7.43 (m, 2H, Ph*H*); ^13^C NMR (CDCl_3_, 100 MHz) *δ*: 18.2 (*C*H_3_), 53.5 (2C, *C*H_2_N(CH_2_)*C*H_2_), 57.1 (O*C*H_3_), 63.1 (CH_3_*C*H(O)CH_2_), 66.6 (2C, *C*H_2_O*C*H_2_), 68.4 (N*C*H_2_CH), 82.4 (*C*HC=O), 127.2 (2C, *o*-Ph), 128.3 (2C, *m*-Ph), 128.4 (*p*-Ph), 136.3 (*Ph*CH), 169.9 (C=O); MS (ESI) *m*/*z* [M+H]^+^ calcd for C_16_H_24_NO_4_^+^ 294.1705, found 294.1561.

### 3.13. General Procedure for the Synthesis of 4-[(2R)-(–)-2-Chloropropyl]morpholine (R)-(–)-***5*** or 4-[(2S)-(+)-2-Chloropropyl]morpholine (S)-(+)-***5***

*Method A:* To a stirred solution of the respective optically active alcohol (*S*)-(+)-**3** (99% ee) or (*R*)-(–)-**3** (98% ee) (10 g, 68.87 mmol) in CHCl_3_ (100 mL), a solution of SOCl_2_ (16.4 g, 0.14 mol, 10 mL) in CHCl_3_ (50 mL) was added dropwise at 0–5 °C under nitrogen atmosphere within a period of 1 h. Next, the resultant reaction mixture was stirred for 2 h at room temperature (ca. 25 °C) until hydrochloride of morpholino derivative precipitated as a heavy-pasty white solid. Then, the solution was refluxed (the solid has been dissolved meantime) until completion of starting material (followed by TLC, approx. 2 h) and stopped by quenching the content of the flask with saturated NaHCO_3_ (2 × 100 mL). The water phase was extracted with CHCl_3_ (3 × 100 mL), the combined organic layer was dried over anhydrous MgSO_4_, the drying agent was filtered off, and the permeate was concentrated in a vacuum. The crude reaction mixture was purified on silica gel column chromatography eluting with a mixture of CHCl_3_/MeOH (95:5, *v*/*v*) to afford (*R*)-(–)-**5** (8.05 g, 71% yield, >99% ee, [α]_D_^22^ = –19.87 (*c* 2.3, CHCl_3_); obtained from (*S*)-(+)-**3**] or (*S*)-(+)-**5** [7.78 g, 69%, 98% ee, [α]_D_^22^ = +23.00 (*c* 1.3, CHCl_3_); obtained from (*R*)-(–)-**3**) as pale amber oil, respectively. The enantiomeric excesses (% ee) of (*R*)-(–)-**5** and (*S*)-(+)-**5** were determined after thioetherification of the respective chloro-derivative with sodium thiophenolate (for details, see Section 3.14).

*Method B:* A solution of (*S*)-(+)-**5** (5 g, 34.4 mmol) and CCl_4_ (7.95 g, 51.6 mmol) in dry CH_2_Cl_2_ (30 mL) was cooled to 0–5 °C. Next, triphenylphosphine (Ph_3_P, 13.5 g, 51.6 mmol) was added portion-wise via a powder funnel over 30 min with vigorous stirring. Upon addition of the phosphine, the colorless solution turned a pale brown color and was stirred for an additional 6 h at room temperature (ca. 25 °C). Next, EtOAc (50 mL) was added to the remaining mixture with vigorous stirring until the formation of white precipitate, which was filtered off, and washed with cold EtOAc (10 mL). The combined solutions were concentrated in a vacuum, and the remaining oil was subjected to silica gel column chromatography using a mixture of *n*-hexane/EtOAc (50:50, *v*/*v*) to afford (*R*)-(–)-**5** (3.2 g, 57% yield, >99% ee) as a yellowish oil. The enantiomeric excesses (% ee) of (*R*)-(–)-**5** and (*S*)-(+)-**5** were determined after thioetherification of chloro-derivatives with sodium thiophenolate (for details, see Section 3.14).

4-[(2*R*)-(–)-2-Chloropropyl]morpholine [(*R*)-(–)-**5**] or 4-[(2*S*)-(+)-2-chloropropyl]morpholine ((*S*)-(+)-**5**). *R*_f_ (CHCl_3_/MeOH 95:5, *v*/*v*) 0.75 or *R*_f_ (*n*-hexane/EtOAc 50:50, *v*/*v*) 0.55; ^1^H NMR (CDCl_3_, 400 MHz) *δ*: 1.51 (d, *J* = 6.6 Hz, 3H, C*H*_3_), 2.42–2.55 (m, 5H, C*H*_2_N(CH_2_)C*H*_2_ and partially NC*H*_2_CH), 2.65 (dd, *J* = 13.0, 7.0 Hz, 1H, one of NC*H*_2_CH), 3.65–3.74 (m, 4H, C*H*_2_OC*H*_2_), 4.02–4.12 (m, 1H, C*H*); ^13^C NMR (CDCl_3_, 100 MHz) *δ*: 23.3 (*C*H_3_), 53.9 (2C, *C*H_2_N(CH_2_)*C*H_2_), 54.1 (*C*H), 66.8 (N*C*H_2_CH), 66.8 (2C, *C*H_2_O*C*H_2_); FT-IR (neat) *ν*_max_ (cm^−1^): 2961, 2931, 2856, 2812, 1454, 1373, 1298, 1277, 1144, 1115, 1070, 1035, 1011, 934, 903, 864, 800, 684, 635, 622, 472; U*V*/*V*IS: λ_max_ = 208 nm (EtOH); MS (ESI) *m*/*z* [M+H]^+^ calcd for C_7_H_15_ClNO^+^ 164.0842, found 164.0877; GC (80–260 (10 °C/min)): *t_R_* = 4.66.

### 3.14. General Procedure for the Synthesis of (R)- and (S)-4-[2-(Phenylsulfanyl)propyl]morpholine (R)-***5***–**SPh** and (S)-***5***–**SPh**

Clean sodium metal (281 mg, 12.22 mmol) was added portion-wise to absolute EtOH (5 mL) and stirred for 1 h at room temperature (ca. 25 °C). Next, a solution of thiophenol (1.35 g, 12.22 mmol) in anhydrous EtOH (2 mL) was added under an argon atmosphere, and the mixture was stirred for 30 min at room temperature (ca. 25 °C). Afterward, the respective optically active 4-(2-chloropropyl)morpholine ((*R*)-(–)-**5** or (*S*)-(+)-**5**, 200 mg, 1.22 mmol) was added in one portion, and the reaction mixture was stirred for additional 12 h. The residue was treated with H_2_O (20 mL), and the aqueous phase was extracted with CH_2_Cl_2_ (3 × 25 mL). After drying the combined organic layer over anhydrous MgSO_4_ and evaporation of the volatiles under vacuum, the crude product was purified by column chromatography (SiO_2_) using a gradient of *n*-hexane/EtOAc (75:25, 50:50, *v*/*v*) mixture to afford desired optically active sulfide (*R*)-**5**–**SPh** (42 mg, 15% yield, 98% ee) or (*S*)-**5**–**SPh** (47 mg, 16% yield, >99% ee) as a pale-yellow oil.

(*R*)-4-[2-(Phenylsulfanyl)propyl]morpholine ((*R*)-**5**–**SPh**) or (*S*)-4-[2-(phenylsulfanyl)propyl]morpholine ((*S*)-**5**–**SPh**). *R*_f_ (*n*-hexane/EtOAc 50:50, *v*/*v*) 0.38; ^1^H NMR (CDCl_3_, 400 MHz) *δ*: 1.15 (d, *J* = 6.3 Hz, 3H, C*H*_3_), 2.47–2.60 (m, 4H, C*H*_2_N(CH_2_)C*H*_2_ and partially NC*H*_2_CH), 2.77–2.85 (m, 2H, NC*H*_2_CH), 3.14–3.26 (m, 1H, C*H*), 3.62–3.76 (m, 4H, C*H*_2_OC*H*_2_), 7.13–7.19 (m, 1H, Ph), 7.24–7.30 (m, 2H, Ph), 7.31–7.37 (m, 2H, Ph); ^13^C NMR (CDCl_3_, 100 MHz) *δ*: 14.2 (*C*H_3_), 37.5 (*C*H), 48.8 (*C*H_2_N(CH_2_)*C*H_2_), 58.9 (N*C*H_2_CH), 67.2 (2C, *C*H_2_O*C*H_2_), 125.7 (*p*-Ph), 128.8 (2C, Ph), 128.9 (2C, Ph), 137.1 (*Ph*CH); U*V*/*V*IS: λ_max_ = 254 nm (EtOH); MS (ESI) *m*/*z* [M+H]^+^ calcd for C_13_H_20_NOS^+^ 238.1266, found 238.1358; GC (80–260 (10 °C/min)): *t_R_* = 15.24; HPLC (*n*-hexane/*tert*-ButOH/Et_3_N (96.5:3.0:0.5, *v*/*v*/*v*); f = 1.0 mL/min; λ = 254 nm; *T* = 30 °C, Chiralcel OJ-H): *t_R_ =* 19.417 min (*R*-isomer) and 20.940 min (*S*-isomer).

### 3.15. General Procedure for the Synthesis of Diphenylacetic Acid Chloride (***8***)

To a solution of diphenylacetic acid (**7**, 10 g, 47.12 mmol) in benzene (25 mL) SOCl_2_ (84 g, 0.71 mol, 51 mL) was added dropwise under a nitrogen atmosphere. The reaction mixture was refluxed with stirring for 6 h, then cooled to room temperature and stirred for an additional 24 h. The progress of the reaction was controlled using TLC and the mixtures of PhCH_3_/EtOAc (50:10, *v*/*v*) or *n*-hexane/EtOAc (70:10, *v*/*v*) as the eluent systems, respectively. Next, the excess of SOCl_2_ and benzene was removed under vacuum to obtain desired acid chloride **8** (10.5 g, 97% yield) as a colorless oil, which solidified on standing. White solid; mp 49–50 °C (benzene) (Ref. [67] 50.5–51.5 °C (no data)); *R*_f_ (PhCH_3_/EtOAc 50:10, *v*/*v*) 0.76 or *R*_f_ (*n*-hexane/EtOAc 70:10, *v*/*v*) 0.60; ^1^H NMR (CDCl_3_, 400 MHz) *δ*: 5.53 (s, 1H, C*H*), 7.24–7.62 (m, 10H, Ph); ^13^C NMR (CDCl_3_, 100 MHz) *δ*: 68.6 (*C*H), 128.1 (2C, *p*-Ph), 128.6 (4C, *m*-Ph), 128.9 (4C, *o*-Ph), 136.1 (2C, *Ph*CH), 173.4 (C=O).

### 3.16. General Procedure for the Synthesis of N-Diphenylacetyl-1-pyrrolidine (***9***)

A solution of diphenylacetyl chloride (**8**, 10.5 g, 45.52 mmol) in 1,4-dioxane (40 mL) was added dropwise into an ice-bath cold solution of pyrrolidine (10 g, 0.14 mol, 11.6 mL) in 1,4-dioxane (30 mL) under argon. After the initial vigorous reaction had subsided, the mixture was stirred for 1 h at 50 °C, chilled to room temperature, and subsequently diluted with H_2_O (50 mL). The progress of the reaction was controlled on TLC plates using mixtures of PhCH_3_/EtOAc (50:10, *v*/*v*) and CHCl_3_/MeOH (95:5, *v*/*v*) for confirmation, respectively. The precipitated crude amide was washed with H_2_O (2 × 75 mL) and purified by recrystallization from a mixture of Et_2_O/MeOH (100 mL; 50:50, *v*/*v*) to afford **9** (10.3 g, 85% yield) as white crystals. Mp 162–163 °C (Et_2_O/MeOH) (Ref. [68] 162–163 °C (Et_2_O/MeOH)); *R*_f_ (PhCH_3_/EtOAc 50:10, *v*/*v*) 0.22 or *R*_f_ (CHCl_3_/MeOH 95:5, *v*/*v*) 0.73; ^1^H NMR (CDCl_3_, 400 MHz) *δ*: 1.78–1.95 (m, 4H, C*H*_2_C*H*_2_) 3.45 (t, *J* = 6.7 Hz, 2H, C*H*_2_NCH_2_), 3.55 (t, *J* = 6.7 Hz, 2H, CH_2_NC*H*_2_), 5.09 (s, 1H, C*H*), 7.21–7.37 (m, 10H, Ph); ^13^C NMR (CDCl_3_, 100 MHz) *δ*: 24.2 (*C*H_2_CH_2_), 26.1 (CH_2_*C*H_2_), 46.1 (*C*H_2_NCH_2_), 46.7 (CH_2_N*C*H_2_), 56.4 (*C*H), 126.8 (2C, *p*-Ph), 128.4 (4C, *o*-Ph), 128.9 (4C, *m*-Ph), 139.4 (*Ph*CH), 170.0 (C=O); FT-IR (neat) *ν*_max_ (cm^−1^): 2970, 2948, 2878, 1739, 1627, 1599, 1495, 1456, 1419, 1358, 1342, 1301, 1266, 1255, 1227, 1190, 1170, 1083, 1039, 917, 865, 758, 749, 725, 708, 695, 626, 562, 493, 474; U*V*/*V*IS: λ_max_ = 219 nm (EtOH); MS (ESI) *m*/*z* [M+H]^+^ calcd for C_18_H_20_NO^+^ 266.1545, found 266.1563; GC (150–260 (10 °C/min)): *t_R_* = 14.83 min.

### 3.17. General Procedure for the Synthesis of 2-[(2R)-2-(Morpholin-4-yl)propoxy]-2,2-diphenyl-1-(pyrrolidin-1-yl)ethan-1-one ((R)-(–)-***10***) and 2-[(2S)-2-(Morpholin-4-yl)propoxy]-2,2-diphenyl-1-(pyrrolidin-1-yl)ethan-1-one ((S)-(+)-***10***)

To a solution of *N*-diphenylacetyl-1-pyrrolidine (**9**, 500 mg, 1.88 mmol) and *N*-benzyl-*N*,*N*,*N*-triethylammonium chloride (TEBA(Cl), 42 mg, 0.19 mmol) in dry DMSO (8 mL), a grounded NaOH (603 mg; 15.07 mmol) was added in gentle flow of molecular O_2_. The resulting mixture was stirred at room temperature for 15 min, and then a solution of chloro-derivative (*R*)-(–)-**5** (400 mg, 2.45 mmol, >99% ee) in dry DMSO (2 mL) was added in one portion, and the resulting mixture was stirred vigorously with a mechanical stirrer for additional 4 h at 40 °C under oxygen provided from a balloon. The reaction was stopped by the addition of H_2_O (20 mL). After extraction of an aqueous phase with Et_2_O (6 × 30 mL), the combined organic layer was dried over anhydrous MgSO_4_, the drying agent was filtered off, and the excess solvent was removed under reduced pressure affording the crude product, which was subsequently purified by PLC using a gradient of *n*-hexane/acetone (30:10, 15:10, *v*/*v*). The appropriate fraction was removed, placed in the round-bottomed flask, suspended in the mixture of *n*-hexane/acetone (150 mL, 10:20, *v*/*v*), and vigorously stirred for 1 h at room temperature. Next, the silica gel was filtered off, washed with portions of *n*-hexane/acetone (2 × 50 mL, 1:1, *v*/*v*), and the permeate was concentrated under reduced pressure resulting in a yellowish semi-solid, which was subsequently dissolved in warm EtOAc (1 mL) and after careful addition of *n*-hexane (0.5 mL) was kept in the fridge for 2 days to afford (*R*)-(–)-**10** (230 mg, 31% yield, 89% ee, [α]_D_^22^ = –22.6 (*c* 1.9, CHCl_3_)) as colorless crystals. The synthesis of optically active (*S*)-(+)-**10** (150 mg, 20% yield, 87% ee, [α]_D_^22^ = +19.2 (*c* 1.8, CHCl_3_)) was carried out essentially by the same route using or (*S*)-(+)-**5** (98% ee) as an alkylating agent. 

2-[(2*R*)-2-(Morpholin-4-yl)propoxy]-2,2-diphenyl-1-(pyrrolidin-1-yl)ethan-1-one ((*R*)-(–)-**10**) or 2-[(2*S*)-2-(morpholin-4-yl)propoxy]-2,2-diphenyl-1-(pyrrolidin-1-yl)ethan-1-one ((*S*)-(+)-**10**). Mp 83–84 °C (*n*-hexane/EtOAc); *R*_f_ (*n*-hexane/acetone 30:10, *v*/*v*) 0.33 or *R*_f_ (*n*-hexane/acetone 15:10, *v*/*v*) 0.51 or *R*_f_ (CH_2_Cl_2_/MeOH 90:10, *v*/*v*) 0.69 or *R*_f_ (CHCl_3_/MeOH 90:10, *v*/*v*) 0.76; ^1^H NMR (CD_3_CN, 500 MHz) *δ*: 1.11 (d, *J* = 6.9 Hz, 3H, C*H*_3_), 1.55–1.79 (m, 4H, C*H*_2_C*H*_2_ in pyrrolidine), 2.44–2.64 (m, 4H, C*H*_2_NC*H*_2_ in morpholine), 2.84–2.95 (m, 1H, C*H*), 3.07–3.25 (m, 3H, CH_2_NC*H*_2_ in pyrrolidine and one of OC*H*_2_CH), 3.41–3.54 (m, 3H, CH_2_NC*H*_2_ in pyrrolidine and one of OC*H*_2_CH), 3.55–3.70 (m, 4H, C*H*_2_OC*H*_2_ in morpholine), 7.16–7.24 (m, 2H, Ph), 7.24–7.40 (m, 4H, Ph), 7.49–7.68 (m, 4H, Ph); ^13^C NMR (CD_3_CN, 126 MHz) *δ*: 13.4 (*C*H_3_), 24.0 (*C*H_2_CH_2_), 47.9 (CH_2_N*C*H_2_ in pyrrolidine), 48.3 (*C*H_2_NCH_2_ in pyrrolidine), 50.8 (*C*H_2_N*CH*_2_ in morpholine), 60.4 (*C*H), 67.7 (O*C*H_2_CH), 68.1 (*C*H_2_O*C*H_2_ in morpholine), 87.4 (*C*), 127.8 (Ph), 128.0 (Ph), 128.9 (Ph), 143.9 (Ph*C*), 169.2 (C=O); ^13^C DEPT-135 NMR (CD_3_CN, 126 MHz) *δ*: 13.4 (*C*H_3_, +ve), 24.0 (*C*H_2_CH_2_, –ve), 47.9 (CH_2_N*C*H_2_ in pyrrolidine, –ve), 48.3 (*C*H_2_NCH_2_ in pyrrolidine, –ve), 50.8 (*C*H_2_N*CH*_2_ in morpholine, –ve), 60.4 (*C*H, +ve), 67.7 (O*C*H_2_CH), 68.1 (*C*H_2_O*C*H_2_ in morpholine, –ve), 127.8 (Ph, +ve), 128.0 (Ph, +ve), 128.9 (Ph, +ve) (+ve refers to positive phasing and –ve refers to negative phasing); FT-IR (neat) *ν*_max_ (cm^−1^): 2966, 2875, 2813, 1631, 1491, 1449, 1406, 1332, 1289, 1268, 1185, 1164, 1116, 1068, 917, 875, 851, 727, 703, 645; U*V*/*V*IS: λ_max_ = 222 nm (EtOH); MS (ESI) *m*/*z* [M+H]^+^ calcd for C_25_H_33_N_2_O_3_^+^ *m*/*z*: 409.2486, found 409.6588; GC-MS (EI, 70 eV) *t_R_* = 18.870 min, *m*/*z* (%): 236 (12.5), 165 (30), 128 (37.5), 100 (100), 56 (28), 42 (13); HPLC (*n*-hexane/2-PrOH (90:10, *v*/*v*); f = 0.8 mL/min; λ = 222 nm; *T* = 30 °C, Chiralcel OD-H): *t_R_ =* 9.026 min (*S*-isomer) and 19.519 min (*R*-isomer). 

### 3.18. XRD Analyses 

#### 3.18.1. Conditions for Crystal Growth of 2-[(2*R*)-2-(Morpholin-4-yl)propoxy]-2,2-diphenyl-1-(pyrrolidin-1-yl)ethan-1-one ((*R*)-(–)-**10**)

Colorless single crystal of sufficient quality for X-ray diffraction (XRD) analysis was prepared using standard vapor diffusion methodology. In this regard, (*R*)-(–)-**10** (15 mg, 89% ee) was dissolved in EtOAc (1 mL) and transferred into an open-neck glass vial (V = 4mL) opened and placed in a bottle that contained *n*-hexane. The outer vessel was sealed, and the composed system was stored at room temperature for 10 days until monocrystals were grown. 

#### 3.18.2. Crystal Structure Determination of (*R*)-(–)-**10**

A colorless single crystal of (*R*)-(–)-**10** (0.67 × 0.42 × 0.25 mm^3^), suitable for X-ray diffraction analysis, was selected under the polarizing microscope, mounted in an inert oil, and transferred to the cold gas stream of the Oxford Diffraction *κ*-CCD Gemini A Ultra diffractometer. Diffraction data were collected at 100.0(1) K with mirror monochromated Cu*K*α radiation. Cell refinement and data collection, as well as data reduction and analysis, were performed with the Crysalis^pro^ software [69]. The absolute-structure of (*R*)-(–)-**10** was solved with the Olex2 [70] using the ShelXT [71] structure solution program (Intrinsic Phasing) and refined with the SHELXL-2018/3 [72] refinement package (Least Squares minimization). All non-hydrogen atoms were refined with anisotropic displacement parameters. Hydrogen atoms attached to carbon atoms were added to the structure model at geometrically idealized coordinates and refined as riding atoms with Uiso(H) = 1.2Ueq(CH and CH_2_) or Uiso(H) = 1.5Ueq(CH_3_). The absolute configuration for the compound molecule (*R*) was successfully determined using anomalous dispersion effects. Flack parameter [73] calculated from 3549 selected quotients (Parsons’ method) [74] equals 0.03(3). Further analysis of the absolute structure assignment was performed using likelihood methods with PLATON [75]. A total of 3677 Bijvoet pairs (coverage of 1.00) were included in the calculations. The resulting value of the Hooft parameter [76] was 0.02(6), with a P3 probability for an inverted structure smaller than 1 × 10^−60^. These results indicated that the absolute-structure has been correctly assigned.

#### 3.18.3. Crystal Data for (*R*)-(–)-**10**

C_25_H_32_N_2_O_3_ (*M* = 408.52 g/mol): monoclinic, space group *P*2_1_ (no. 4), *a* = 11.46510(10) Å, *b* = 9.10430(10) Å, *c* = 21.3925(2) Å, *β* = 98.3240(10)°, *V* = 2209.46(4) Å^3^, *Z* = 4, *T* = 100.0(1) K, *μ*(Cu Kα) = 0.638 mm^−1^, *Dcalc* = 1.228 g/cm^3^, 93508 reflections measured (7.794° ≤ 2Θ ≤ 134.06°), 7903 unique (*R*_int_ = 0.0550, *R*_sigma_ = 0.0184) which were used in all calculations. The final *R*_1_ was 0.0354 (*I* > 2*σ*(I)) and *wR*_2_ was 0.0929 (all data). For details concerning crystal data and structure refinement parameters for (*R*)-(–)-**10**, see Table S6 appended in Supplementary Materials. CCDC-2202212 contains the supplementary crystallographic data for compound (*R*)-(–)-**10**. This can be obtained free of charge on application to CDC, 12 Union Road, Cambridge CB21EZ, UK (Fax: (+44)1223-336-033; email: deposit@ccdc.cam.ac.uk).

## 4. Conclusions

The principal aim of the present work was to design and synthesize a novel potent analog of iso-moramide utilizing molecular docking calculations and a chemoenzymatic methodology. Initial docking studies revealed that the ethereal analog of (*R*)-*iso*-moramide, namely 2-[(2*R*)-2-(morpholin-4-yl)propoxy]-2,2-diphenyl-1-(pyrrolidin-1-yl)ethan-1-one, exhibits substantial affinity toward all three opioid receptors (ORs), with the highest selectivity calculated for *κ*-OR. Based on in silico investigations, it is most likely that the newly designed dextromoramide analog might exhibit reduced side effects and increased analgesic activity compared to the original drug, and therefore, elaboration of its synthesis seemed to be rational. In this regard, a chemoenzymatic synthesis of both enantiomeric forms of a novel ethereal analog of *iso*-moramide, exhibiting potentially high analgesic activity, was developed using classical lipase-catalyzed kinetic resolution (KR) of racemic 1-(morpholin-4-yl)propan-2-ol as a key step. Optimization studies on the enantioselective transesterification of the afore-mentioned racemate revealed that immobilized lipases isolated from *Candida antarctica* type B (Chirazyme L-2, C-2) and *Burkholderia cepacia* (Amano PS-Immobead 150) were the most efficient biocatalysts. The employment of the selected enzymes and appropriate conditions allowed to accomplish a multi-gram scale KR with enantiomerically pure products being obtained–(*S*)-morpholino-alcohol (>99% ee) and the respective (*R*)-acetate (99% ee). The stereochemical preference of the lipase preparations in the transesterification reaction of racemic 1-(morpholin-4-yl)propan-2-ol was determined by the absolute configuration assignment using a modified form of Mosher’s methodology. After comparing ^1^H NMR spectra recorded for diastereomeric esters prepared from the respective chiral auxiliaries, the (*S*)-configuration was assigned to the slower-reacting enantiomer of morpholino-alcohol. The results of the spectroscopic experiments confirmed that all the employed lipases behaved as typical Kazlauskas-type catalysts [77]. The biocatalytic step was followed by convenient NaOH-mediated hydrolysis of the (*R*)-acetate leading to (*R*)-morpholino-alcohol (98% ee) and the subsequent chlorination of both chiral antipodes of hydroxyl-derivatives using SOCl_2_. Further chemical transformations of optically active chlorides employing *N*-diphenylacetyl-1-pyrrolidine under PTC-assisted conditions with the use of TEBA(Cl) as the phase-transfer catalyst and the liquid–solid system comprising grounded NaOH suspended in O_2_-saturated DMSO as an oxidation agent resulted in the preparation of (*R*)-*iso*-moramide-ether in 31% yield and 89% ee from (*R*)-chloride (>99% ee), and (*S*)-*iso*-moramide-ether in 20% yield and 87% ee from (*S*)-chloride (98% ee), respectively. Based on the literature, a plausible reaction mechanism was proposed for a “one-pot” α-hydroxylation/etherification reaction indicating as this transformation partially follows the Russell mechanism concerning the oxidation of di-benzylic carbonation and subsequent DMSO-mediated reduction of the generated peroxide followed by regioselective ring-opening of the in situ formed aziridinium ion by α-hydroxylated *N*-diphenylacetyl-1-pyrrolidine. The suitable monocrystals of the synthesized active agent were obtained by slow evaporation/precipitation from a solution using a standard diffusion methodology, and their structures were determined by single crystal X-ray diffraction. Further in vitro and in vivo pharmacological characterization of this compound is required. 

## Figures and Tables

**Table 2:**
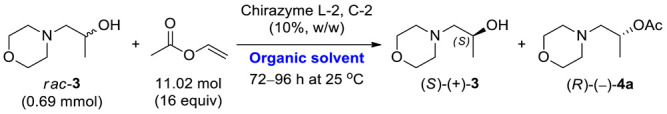
Solvent screening for Chirazyme L-2, C-2-catalyzed enantioselective acetylation of *rac*-**3** using vinyl acetate under kinetically controlled conditions.

Entry	Solvent ^a^ (log*P*) ^b^	*t* (h)	Conv. (%) ^c^	ee_s_ (%) ^d^	ee_p_ (%) ^e^	*E* ^f^
1	1,4-Dioxane (–0.31)	96	56	76	60	9
2	CH_3_CN (0.17)	96	57	93	70	19
3	Acetone (0.20)	96	53	87	77	21
4	THF (0.40)	72	54	97	83	45
5	Et_2_O (0.76)	72	57	98	74	30
6	MTBE (0.96)	72	57	99	75	35
7	*tert*-Amyl alcohol (1.09)	96	55	97	79	35
8	*n*-Hexane (3.00)	72	55	95	78	29

^a^ Conditions: *rac*-**3** 100 mg, Chirazyme L-2, C-2 10 mg, organic solvent 1 mL, vinyl acetate 948 mg (16 equiv), 25 °C, 500 rpm; ^b^ Logarithm of the partition coefficient of a given solvent between *n*-octanol and water according to ChemBioDraw Ultra 13.0 software; ^c^ Determined by GC analysis using a calibration curve; ^d^ Determined by HPLC analysis using a chiral stationary phase; ^e^ Determined by chiral HPLC analysis of the corresponding alcohol (*R*)-(–)-**3** obtained after NaOH-catalyzed methanolysis of (*R*)-(–)-**4a**; ^f^ Calculated according to Chen et al. [45], using the equation: *E* = {ln[(1 − conv.)(1 − ee_s_)]}/{ln[(1 − conv.)(1 + ee_s_)]}.

**Table 3:**
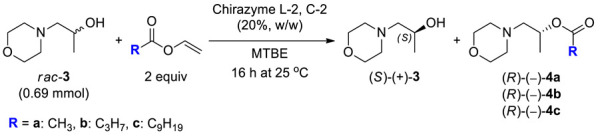
The influence of acyl donor on Chirazyme L-2, C-2-catalyzed enantioselective transesterification of *rac*-**3** in MTBE after 16 h.

Entry	Acyl Donor ^a^	Conv. (%) ^b^	ee_s_ (%) ^c^	ee_p_ (%) ^d^	*E* ^e^
1	Vinyl acetate	55	>99	81	49
2	Vinyl butanoate	54	>99	84	59
3	Vinyl decanoate	55	>99	80	46

^a^ Conditions: *rac*-**3** 100 mg, Chirazyme L-2, C-2 20 mg, MTBE 1 mL, vinyl ester (2 equiv), 16 h at 25 °C, 500 rpm; ^b^ Determined by GC analysis using a calibration curve; ^c^ Determined by HPLC analysis using a chiral stationary phase; ^d^ Determined by chiral HPLC analysis of the corresponding alcohol (*R*)-(–)-**3** obtained after NaOH-catalyzed methanolysis of (*R*)-(–)-**4a**–**c**; ^e^ Calculated according to Chen et al. [45], using the equation: *E* = {ln[(1 − conv.)(1 − ee_s_)]}/{ln[(1 − conv.)(1 + ee_s_)]}.

**Table 4:**
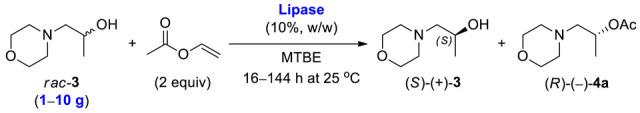
Gram- and multigram-scale lipase-catalyzed enantioselective transesterification of *rac*-**3**.

Entry	Scale (g)	Lipase Preparation	*t* (h)	Conv. (%) ^a^	ee_s_ (%) ^b^/ Yield ^c^ (%)	ee_p_ (%) ^d^/ Yield ^c^ (%)	*E* ^e^
1	1.0 ^f^	Chirazyme L-2, C-2	16	57	>99/34	75/40	35
2	1.0 ^g^	Amano PS-Immobead 150	144	33	49/62	99/28	324
3	10.0 ^h^	Chirazyme L-2, C-2	27	56	>99/36	79/38	44

^a^ Determined by GC analysis using a calibration curve; ^b^ Determined by HPLC analysis using a chiral stationary phase; ^c^ Isolated yield after column chromatography; ^d^ Determined by chiral HPLC analysis of the corresponding alcohol (*R*)-(–)-**3** obtained after NaOH-catalyzed methanolysis of (*R*)-(–)-**4a**; ^e^ Calculated according to Chen et al. [45], using the equation: *E* = {ln[(1 − conv.)(1 − ee_s_)]}/{ln[(1 − conv.)(1 + ee_s_)]}; ^f^ Conditions: *rac*-**3** 1 g, Chirazyme L-2, C-2 200 mg, MTBE 10 mL, vinyl acetate 1.19 g or 1.27 mL (2 equiv), 25 °C, 500 rpm; ^g^ Conditions: *rac*-**3** 1 g, Amano PS-Immobead 150 200 mg, MTBE 10 mL, vinyl acetate 1.19 g or 1.27 mL (2 equiv), 25 °C, 500 rpm; ^h^ Conditions: *rac*-**3** 10 g, Chirazyme L-2, C-2 2 g, MTBE 100 mL, vinyl acetate 11.89 g or 12.72 mL (2 equiv), 25 °C, 500 rpm.

## Data Availability

Not applicable. The data presented in this article are openly available.

## Supplementary Materials

The following supporting information can be downloaded at: https://www.mdpi.com/article/10.3390/ijms231911803/s1.
