# Supplementary material for: Chemoenzymatic Synthesis of Optically Active Ethereal Analog of iso-Moramide—A Novel Potentially Powerful Analgesic
Source: Int J Mol Sci. 2022 Oct 5;23(19):11803. doi: 10.3390/ijms231911803 (PMC9569485; doi:10.3390/ijms231911803)

Supporting Information

for

Chemoenzymatic Synthesis of Optically Active Ethereal Analog of *iso*-Moramide – a Novel Potentially Powerful Analgesic

Paweł Borowiecki

Laboratory of Biocatalysis and Biotransformation, Department of Drugs Technology and Biotechnology, Warsaw University of Technology, Koszykowa St. 75, 00–662 Warsaw, Poland. Correspondence: pawel.borowiecki@pw.edu.pl

Table of contents

Table S1. List of commercial enzyme preparations employed in these studies …..……….. S2

Table S2. The results of specific rotation values for optically active products …………..… S2

Table S3. Analytical separation conditions of studied compounds by GC column ……….… S3

Table S4. HPLC analytical separation conditions of racemic compounds ....… S4

Table S5. Docking scoring for ligands complexed with opioid receptors (ORs) …….... S5–S8

Table S6. Crystal data and structure refinement parameters for (*R*)-(–)-**10** ………………… S9

Copies of HPLC chromatograms …...……….……………………………….............. S10–S12

Copies of NMR, MS, and FT-IR spectra ..……...…………………...……….…..... S13–S36

Table S1. List of commercial enzyme preparations employed in these studies.

Enzyme and its origin (microorganism/tissue)	Enzyme preparation[a] (brand name)	Usage form of enzyme preparation	Enzyme specified activity	Commercial supplier
Lipase from *Candida antarctica* B (CAL-B)	**Novozym 435**	immobilized on macroporous acrylic resin [poly (methyl methacrylate-co-butyl methacrylate)]	>10000 U/g or 10 PLU/mg, water content 1.4%	Novozymes A/S
	**Chirazyme L-2, c.-f., C2, Lyo.**	immobilized on the carrier-fixed (carrier 2)	150 kU	Roche
	**Chirazyme L-2, c.-f., C3, Lyo.**	immobilized on the carrier-fixed (carrier 3)	150 kU	Roche
Lipase from *Candida antarctica* A (CAL-A)	**Chirazyme L-5**	native	unspecified	Boehringer Mannheim[b]
Lipase from *Burkholderia cepacia* (formerly *Pseudomonas cepacia*)	**Amano PS**	native	>23.000 U/g	Amano Pharmaceutical Co., Ltd.
	**Amano PS-IM**	immobilized on diatomite	500 U/g	Amano Pharmaceutical Co., Ltd.
	**PS-Immobead 150**	immobilized on Immobead 150	≥900 U/g	Sigma-Aldrich
Lipase from *Pseudomonas fluorescens*	**Amano AK**	native	>20.000 U/g	Amano Pharmaceutical Co., Ltd.

[a] All commercial formulations of enzymes studied herein were used without pre-treatment.
[b] Currently: Roche Diagnostics.

Table S2. The results of specific rotation values for optically active products.

Compound	ee [%]	Measured specific rotation $[\alpha]_D$	Literature specific rotation $[\alpha]_D^{lit.}$
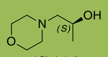 (*S*)-(+)-**3**	>99	$[\alpha]_D^{27.5}$ = +50.40 (*c* 1.3, $CHCl_3$)	Lack of data
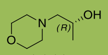 (*R*)-(–)-**3**	98	$[\alpha]_D^{22}$ = –73.03 (*c* 1.8, $CHCl_3$)	Lack of data
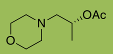 (*R*)-(–)-**4a**	99	$[\alpha]_D^{27.5}$ = –4.66 (*c* 1.2, $CHCl_3$)	Lack of data
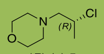 (*R*)-(–)-**5**	>99	$[\alpha]_D^{22}$ = –19.87 (*c* 2.3, $CHCl_3$)	Lack of data
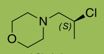 (*S*)-(+)-**5**	98	$[\alpha]_D^{22}$ = +23.00 (*c* 1.3, $CHCl_3$)	Lack of data
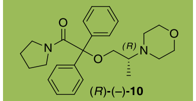 (*R*)-(–)-**10**	89	$[\alpha]_D^{22}$ = –22.6 (*c* 1.9, $CHCl_3$)	Lack of data
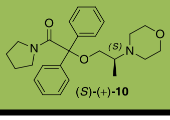 (*S*)-(+)-**10**	87	$[\alpha]_D^{22}$ = +19.2 (*c* 1.8, $CHCl_3$)	Lack of data

Table S3. Analytical separation conditions of studied compounds by GC column.

Compound	Temperature program [°C]	Retention time [min]
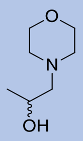 *rac*-**3**	80–260 (10 °C/min)	4.43
	120–260 (10 °C/min)	2.20
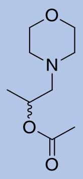 *rac*-**4a**	80–260 (10 °C/min)	6.39
	120–260 (10 °C/min)	3.28
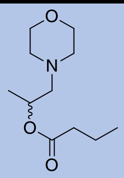 *rac*-**4b**	120–260 (10 °C/min)	4.98
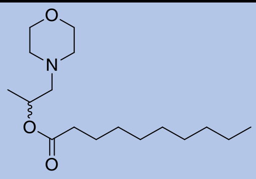 *rac*-**4c**	120–260 (10 °C/min)	11.15
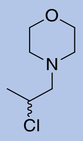 *rac*-**5**	80–260 (10 °C/min)	4.66
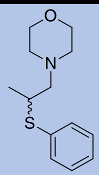 *rac*-**5–SPh**	80–260 (10 °C/min)	15.24
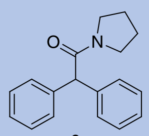 **9**	150–260 (10 °C/min)	14.83
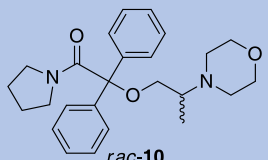 *rac*-**10**	250–260 (10 °C/min) and then 15 min at 260 °C	30.45

Table S4. HPLC analytical separation conditions of racemic compounds.

Compound	HPLC Column	Mobile Phase	Flow Rate [mL/min] / Pressure [MPa]	Detection [nm] / Temperature [°C]	Retention Time [min]
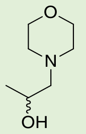 *rac*-**3**	Chiralpak AD-H	*n*-hexane/EtOH (90:10, v/v)	0.6 / 2.7	208 / 30	14.611 (*R*) and 16.213 (*S*)
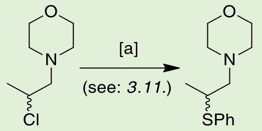 *rac*-**5** → *rac*-**5–SPh**	Chiralcel OJ-H	*n*-hexane/*tert*-ButOH/Et_3N (96.5:3.0:0.5, v/v/v)	1.0 / 4.4	254 / 30	19.417 (*R*) and 20.940 (*S*)
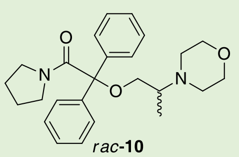 *rac*-**10**	Chiralcel OD-H	*n*-hexane/2-PrOH (90:10, v/v)	0.8 / 3.0	222 / 30	9.026 (*S*) and 19.519 (*R*)

[a] Derivatization of *rac*-**5** into *rac*-**5**–**SPh** was performed using thiophenol (10 equiv) in EtONa (10 equiv)/EtOH, 12 h at 25 °C.

Table S5. Docking scoring of the respective ligands complexed with opioid receptors (ORs).

Entry	Ligand	OR	Pose[a]	Affinity (kcal/mol)	Distance from best mode[b]	
					rmsd l.b.	rmsd u.b.
1			S1	–7.9	0.000	0.000
2			S2	–7.7	8.375	10.081
3			S3	–7.5	2.560	4.445
4			S4	–7.1	8.092	10.366
5		*μ*-OR (PDB ID: 4DKL)	S5	–6.9	9.020	10.999
6			S6	–6.9	9.524	11.945
7			S7	–6.9	8.563	11.057
8			S8	–6.8	8.938	11.097
9			S9	–6.7	8.338	10.729
10			S1	–7.6	0.000	0.000
11			S2	–7.3	2.561	4.265
12			S3	–6.1	2.275	4.311
13			S4	–6.1	15.622	17.950
14	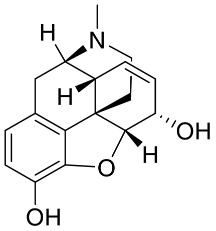 Morphine	*δ*-OR (PDB ID: 4EJ4)	S5	–6.1	12.937	15.126
15			S6	–6.0	16.654	18.709
16			S7	–6.0	11.256	13.364
17			S8	–6.0	11.356	13.831
18			S9	–6.0	3.416	5.155
19			S1	–7.8	0.000	0.000
20			S2	–7.8	3.145	4.663
21			S3	–7.8	11.398	12.939
22			S4	–7.8	2.638	4.239
23		*κ*-OR (PDB ID: 4DJH)	S5	–7.6	2.110	2.888
24			S6	–7.6	1.824	3.964
25			S7	–7.5	1.516	2.032
26			S8	–7.4	10.564	12.414
27			S9	–7.0	3.386	5.235
28			S1	–8.4	0.000	0.000
29			S2	–8.4	0.062	1.510
30			S3	–8.3	0.081	1.510
31			S4	–8.3	0.142	1.076
32		*μ*-OR (PDB ID: 4DKL)	S5	–8.1	1.673	7.375
33			S6	–8.1	1.691	7.473
34			S7	–7.8	2.203	8.255
35			S8	–7.5	3.108	4.614
36			S9	–7.5	1.260	1.769
37			S1	–7.0	0.000	0.000
38			S2	–6.9	3.035	5.002
39			S3	–6.8	14.751	16.236
40			S4	–6.8	2.156	3.990
41	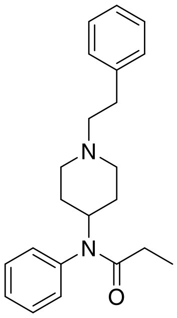 Fentanyl	*δ*-OR (PDB ID: 4EJ4)	S5	–6.8	1.602	2.427
42			S6	–6.8	2.894	8.772
43			S7	–6.7	2.248	9.467
44			S8	–6.7	2.221	3.584
45			S9	–6.7	2.409	3.288
46			S1	–7.7	0.000	0.000
47			S2	–7.7	11.001	12.666
48			S3	–7.6	12.524	15.704
49			S4	–7.6	0.223	1.520
50		*κ*-OR (PDB ID: 4DJH)	S5	–7.5	12.552	15.802
51			S6	–7.3	4.340	8.252
52			S7	–7.3	11.202	14.990
53			S8	–7.3	4.132	8.022
54			S9	–7.3	13.137	16.068

[a] The pose S1 represents the lowest value of ΔG_{calc} (kcal/mol), which means that ligand-binding affinity to receptor is the highest, and in contrary, the S9 mode represent the lowest ligand-binding affinity.

[b] The values <2.000 rmsd represent the closest distance between the ligand and the opioid receptor binding site.

Table S5. Docking scoring of the respective ligands complexed with opioid receptors (ORs) – continued.

Entry	Ligand	OR	Pose[a]	Affinity (kcal/mol)	Distance from best mode[b]	
					rmsd l.b.	rmsd u.b.
55		μ-OR (PDB ID: 4DKL)	S1	–9.3	0.000	0.000
56			S2	–8.6	1.192	1.574
57			S3	–8.6	2.731	3.870
58			S4	–8.5	1.368	1.562
59			S5	–8.1	2.264	3.451
60			S6	–7.9	3.486	7.660
61			S7	–7.8	1.808	2.161
62			S8	–7.8	3.288	6.639
63			S9	–7.8	2.963	3.857
64	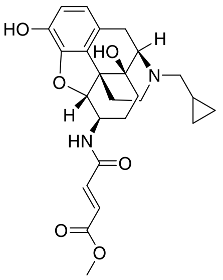	δ-OR (PDB ID: 4EJ4)	S1	–9.1	0.000	0.000
65			S2	–8.8	1.155	1.388
66			S3	–8.8	2.185	3.462
67			S4	–8.6	1.678	2.752
68			S5	–8.6	2.685	3.212
69			S6	–7.7	1.899	3.232
70			S7	–7.6	2.481	4.227
71			S8	–7.4	14.717	17.865
72	β-funaltrexamine (β-FNA)		S9	–7.3	15.226	18.108
73		κ-OR (PDB ID: 4DJH)	S1	–7.9	0.000	0.000
74			S2	–7.9	3.539	7.041
75			S3	–7.9	13.462	16.581
76			S4	–7.9	7.479	10.610
77			S5	–7.9	16.239	19.390
78			S6	–7.9	3.626	7.199
79			S7	–7.8	14.123	16.680
80			S8	–7.7	13.193	16.407
81			S9	–7.7	1.413	1.797
82		μ-OR (PDB ID: 4DKL)	S1	–9.2	0.000	0.000
83			S2	–9.2	2.075	10.006
84			S3	–9.1	2.934	10.105
85			S4	–8.9	3.228	4.971
86			S5	–8.9	2.877	4.179
87			S6	–8.8	5.779	10.009
88	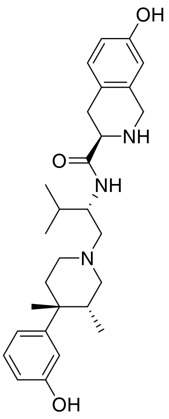		S7	–8.8	2.862	3.947
89			S8	–8.7	2.939	10.007
90			S9	–8.6	4.945	9.454
91		δ-OR (PDB ID: 4EJ4)	S1	–8.7	0.000	0.000
92			S2	–8.6	3.481	8.576
93			S3	–8.4	3.717	6.056
94			S4	–8.3	4.658	5.353
95			S5	–8.2	4.137	8.592
96			S6	–8.2	4.434	9.199
97			S7	–8.1	11.821	16.216
98			S8	–8.0	10.812	13.721
99			S9	–8.0	4.637	5.631
100		κ-OR (PDB ID: 4DJH)	S1	–9.6	0.000	0.000
101			S2	–9.2	3.905	7.474
102	JDTic		S3	–9.0	1.990	2.768
103			S4	–9.0	4.308	7.534
104			S5	–8.8	3.909	6.425
105			S6	–8.8	11.291	14.030
106			S7	–8.8	3.722	5.290
107			S8	–8.7	4.169	6.220
108			S9	–8.7	4.369	7.899

[a] The pose S1 represents the lowest value of ΔG_{calc} (kcal/mol), which means that ligand-binding affinity to receptor is the highest, and in contrary, the S9 mode represent the lowest ligand-binding affinity.

[b] The values <2.000 rmsd represent the closest distance between the ligand and the opioid receptor binding site.

Table S5. Docking scoring of the respective ligands complexed with opioid receptors (ORs) – continued.

Entry	Ligand	OR	Pose[a]	Affinity (kcal/mol)	Distance from best mode[b]	
					rmsd l.b.	rmsd u.b.
109	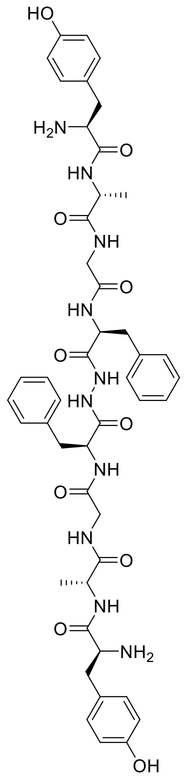 Biphalin	μ-OR (PDB ID: 4DKL)	S1	–9.3	0.000	0.000
110			S2	–9.3	2.443	5.912
111			S3	–9.2	2.533	8.272
112			S4	–9.2	2.641	7.153
113			S5	–9.1	2.229	7.540
114			S6	–9.0	2.367	4.987
115			S7	–9.0	2.183	6.312
116			S8	–8.9	2.653	6.808
117			S9	–8.9	2.708	6.123
118		δ-OR (PDB ID: 4EJ4)	S1	–9.9	0.000	0.000
119			S2	–9.6	3.459	5.022
120			S3	–9.5	3.034	12.152
121			S4	–9.5	2.363	4.021
122			S5	–9.4	1.626	2.563
123			S6	–9.3	1.822	14.672
124			S7	–9.3	2.252	3.463
125			S8	–9.2	5.071	8.288
126			S9	–9.2	3.160	5.744
127		κ-OR (PDB ID: 4DJH)	S1	–9.6	0.000	0.000
128			S2	–9.6	2.070	7.564
129			S3	–9.5	2.074	8.131
130			S4	–9.2	2.168	7.994
131			S5	–9.0	2.156	7.687
132			S6	–8.9	1.637	2.271
133			S7	–8.9	2.058	8.762
134			S8	–8.9	2.345	6.662
135			S9	–8.8	2.307	9.438
136	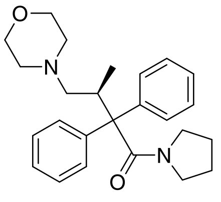 Dextromoramide	μ-OR (PDB ID: 4DKL)	S1	–8.0	0.000	0.000
137			S2	–7.8	1.461	4.753
138			S3	–7.7	1.695	4.147
139			S4	–7.7	2.330	5.998
140			S5	–7.6	2.177	5.119
141			S6	–7.5	1.580	2.295
142			S7	–7.5	5.479	8.762
143			S8	–7.5	1.816	6.490
144			S9	–7.4	3.921	6.438
145		δ-OR (PDB ID: 4EJ4)	S1	–8.0	0.000	0.000
146			S2	–7.8	2.128	6.156
147			S3	–7.3	1.561	2.200
148			S4	–7.3	2.018	5.746
149			S5	–7.3	1.745	4.636
150			S6	–7.2	2.038	5.665
151			S7	–7.2	1.823	5.618
152			S8	–7.2	1.921	4.642
153			S9	–7.2	2.032	5.358
154		κ-OR (PDB ID: 4DJH)	S1	–7.8	0.000	0.000
155			S2	–7.8	11.083	13.749
156			S3	–7.8	1.632	4.633
157			S4	–7.7	1.728	2.729
158			S5	–7.7	1.910	4.492
159			S6	–7.7	10.839	13.325
160			S7	–7.6	3.406	7.119
161			S8	–7.6	1.643	4.711
162			S9	–7.6	11.332	14.262

[a] The pose S1 represents the lowest value of ΔG_{calc} (kcal/mol), which means that ligand-binding affinity to receptor is the highest, and in contrary, the S9 mode represent the lowest ligand-binding affinity.

[b] The values <2.000 rmsd represent the closest distance between the ligand and the opioid receptor binding site.

Table S5. Docking scoring of the respective ligands complexed with opioid receptors (ORs) – continued.

Entry	Ligand	OR	Pose[a]	Affinity (kcal/mol)	Distance from best mode[b]	
					rmsd l.b.	rmsd u.b.
163		μ-OR (PDB ID: 4DKL)	S1	–7.8	0.000	0.000
164			S2	–7.7	1.553	4.319
165			S3	–7.7	1.543	2.597
166			S4	–7.7	1.953	4.992
167			S5	–7.6	2.011	4.739
168			S6	–7.6	2.036	4.956
169			S7	–7.5	2.313	5.442
170			S8	–7.5	5.419	8.322
171			S9	–7.5	1.832	4.660
172		δ-OR (PDB ID: 4EJ4)	S1	–7.6	0.000	0.000
173			S2	–7.6	1.411	2.113
174			S3	–7.5	1.531	2.193
175			S4	–7.4	1.299	4.468
176	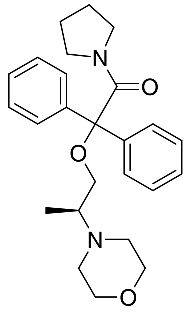		S5	–7.4	1.637	2.355
177			S6	–7.4	1.829	4.822
178			S7	–7.4	1.816	4.790
179			S8	–7.3	1.811	4.398
180			S9	–7.2	1.533	2.076
181	(*S*)-(+)-**10**	κ-OR (PDB ID: 4DJH)	S1	–7.7	0.000	0.000
182			S2	–7.7	1.147	1.947
183			S3	–7.7	10.819	12.732
184			S4	–7.7	1.360	4.496
185			S5	–7.7	11.720	14.686
186			S6	–7.6	12.036	14.375
187			S7	–7.6	1.981	3.482
188			S8	–7.4	12.248	14.632
189			S9	–7.4	11.912	14.636
190		μ-OR (PDB ID: 4DKL)	S1	–8.1	0.000	0.000
191			S2	–7.8	1.411	4.303
192			S3	–7.7	2.084	4.910
193			S4	–7.7	1.393	1.982
194			S5	–7.6	1.553	4.359
195			S6	–7.6	1.915	4.698
196			S7	–7.6	1.508	4.437
197			S8	–7.6	1.856	4.381
198			S9	–7.4	2.197	5.312
199		δ-OR (PDB ID: 4EJ4)	S1	–8.3	0.000	0.000
200			S2	–8.1	2.388	5.043
201			S3	–8.0	2.173	4.981
202			S4	–7.9	2.107	4.966
203	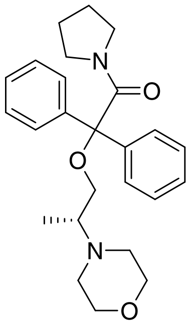		S5	–7.9	2.413	5.153
204			S6	–7.8	2.303	5.990
205			S7	–7.8	1.683	2.551
206			S8	–7.7	2.156	3.143
207			S9	–7.7	2.326	5.235
208	(*R*)-(–)-**10**	κ-OR (PDB ID: 4DJH)	S1	–8.8	0.000	0.000
209			S2	–8.8	10.483	12.202
210			S3	–8.8	10.190	12.758
211			S4	–8.7	10.406	12.386
212			S5	–8.7	1.382	4.591
213			S6	–8.6	11.920	14.495
214			S7	–8.5	12.094	14.633
215			S8	–8.5	2.035	6.436
216			S9	–8.5	1.776	2.540

[a] The pose S1 represents the lowest value of ΔG_{calc} (kcal/mol), which means that ligand-binding affinity to receptor is the highest, and in contrary, the S9 mode represent the lowest ligand-binding affinity.

[b] The values <2.000 rmsd represent the closest distance between the ligand and the opioid receptor binding site.

Table S6. Crystal data and structure refinement parameters for (*R*)-(–)-10.

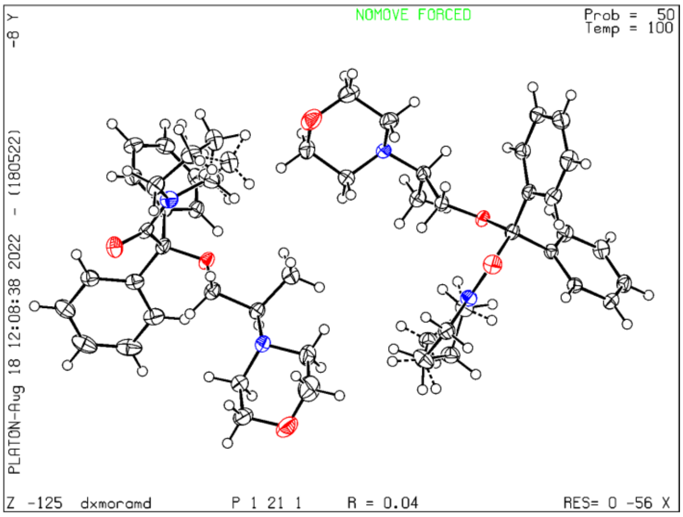

Compound	(*R*)-(–)-**10**
Chemical formula	$C_{25}H_{32}N_2O_3$
M /g·mol^{-1}	408.52
T /K	100.0(1)
Crystal system	monoclinic
Space group	$P2_1$
a/Å	11.4651(1)
b/Å	9.1043(1)
c/Å	21.3925(2)
β/°	98.324(1)
V /Å^3	2209.46(4)
Z	4
D_{calc} /g·cm^{-3}	1.228
μ/mm^{-1}	0.638
F(000)	880.0
Crystal size/mm^3	0.67 × 0.42 × 0.251
Radiation, λ/ Å	CuKα (λ = 1.54184)
2Θ Range /°	7.794 to 134.06
Reflections collected	93508
Independent reflections	7903
R_{int}	0.0550
Data/restraints/parameters	7903/5/552
S (F^2)[a]	1.030
R1, wR2 (I>2$\sigma(I)$)[b]	0.0354, 0.0923
R1, wR2 (all data)	0.0361, 0.0929
$\Delta\rho_{min/max}$/eÅ^{-3}	+0.46/–0.26
Flack parameter	0.01(6)

[a] Goodness-of-fit $S=\{\Sigma[w(F_o^2-F_c^2)^2]/(n-p)\}^{1/2}$ where n is the reflections number and p is the parameters number.
[b] $R1=\Sigma||F_o|-|F_c||/\Sigma|F_o|$, $wR2=\{\Sigma[w(F_o^2-F_c^2)^2]/\Sigma[w(F_o^2)^2]\}^{1/2}$.

HPLC of *rac*-3 on Chiralpak AD-H at 30 °C

Conditions: *n*-hexane-EtOH (90:10, v/v); f=0.6 mL/min; λ=208 nm

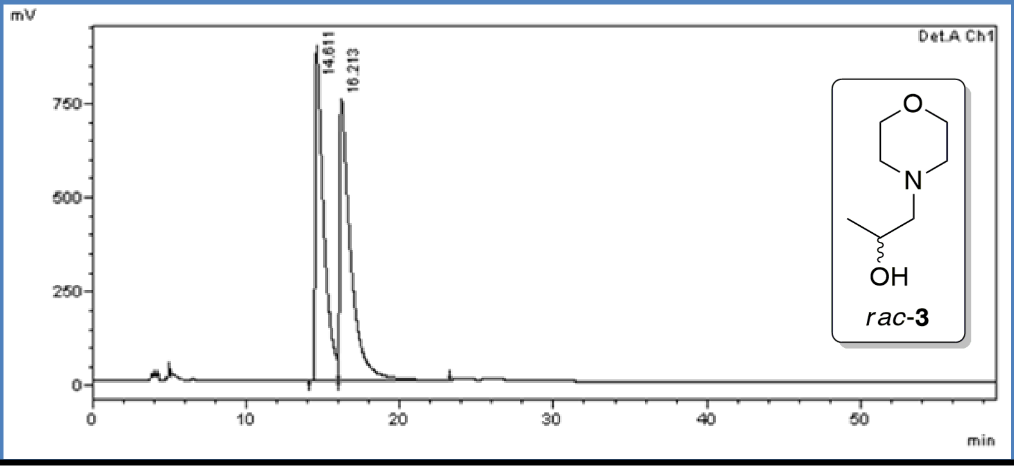

HPLC of (*S*)-(+)-3 on Chiralpak AD-H at 30 °C

Conditions: *n*-hexane-EtOH (90:10, v/v); f=0.6 mL/min; λ=208 nm

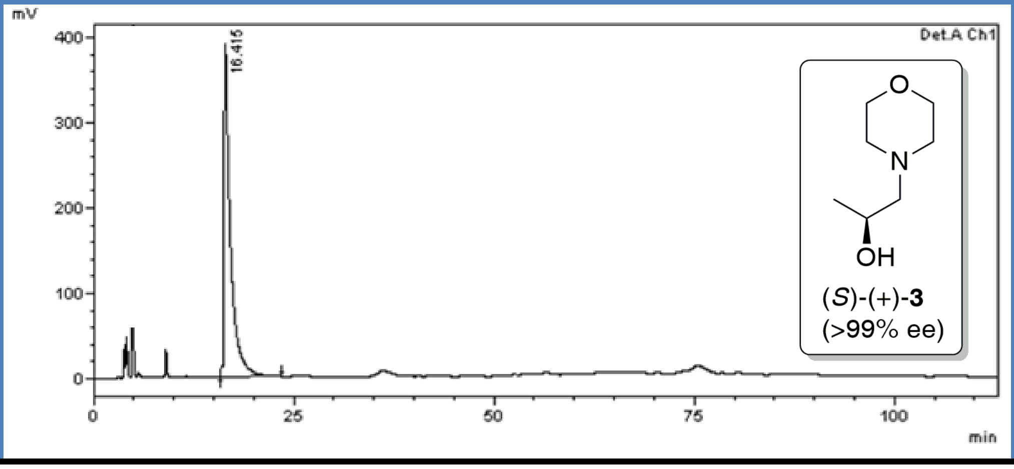

HPLC of (*R*)-(–)-3 on Chiralpak AD-H at 30 °C

Conditions: *n*-hexane-EtOH (90:10, v/v); f=0.6 mL/min; λ=208 nm

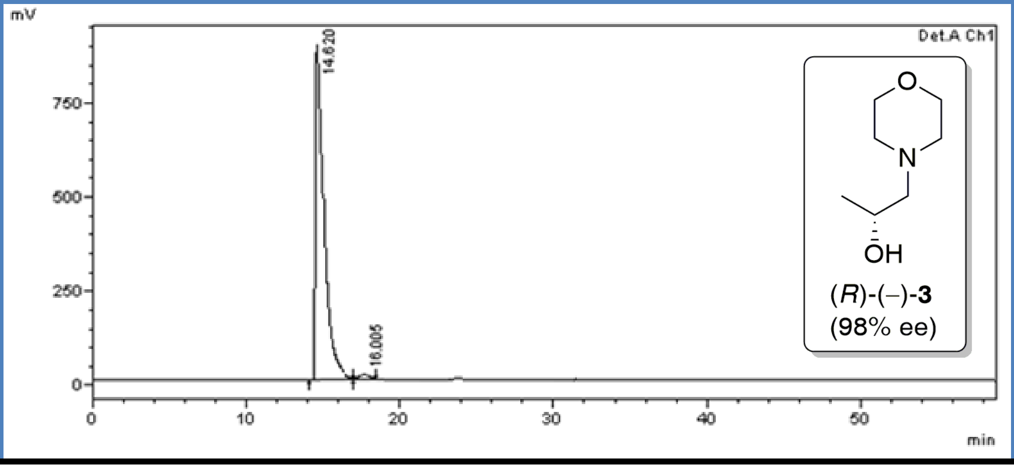

HPLC of *rac*-5-SPh (obtained from *rac*-5) on Chiralcel OJ-H at 30 °C

Conditions: *n*-hexane/*tert*-ButOH/Et_3N (96.5:3.0:0.5, v/v/v); f=1.0 mL/min; λ=254 nm

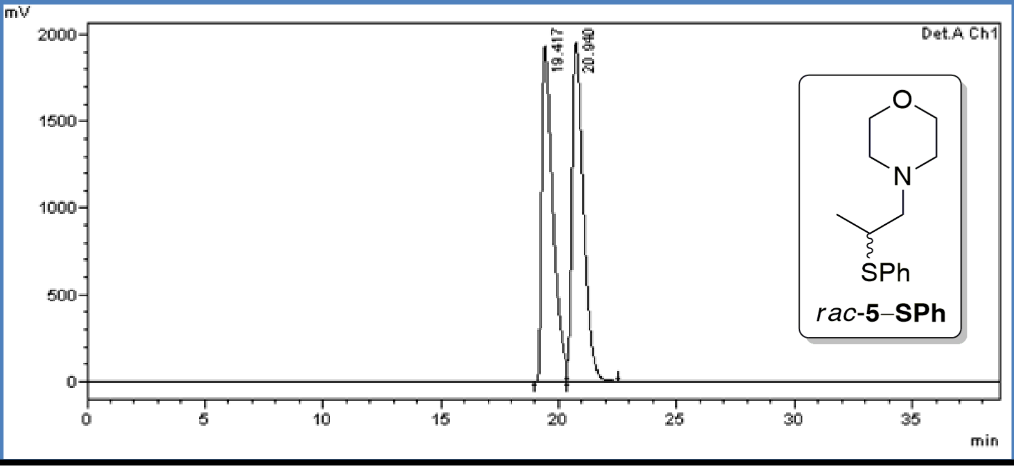

HPLC of (*S*)-5–SPh [obtained from (*R*)-(–)-5] on Chiralcel OJ-H at 30 °C

Conditions: *n*-hexane/*tert*-ButOH/Et_3N (96.5:3.0:0.5, v/v/v); f=1.0 mL/min; λ=254 nm

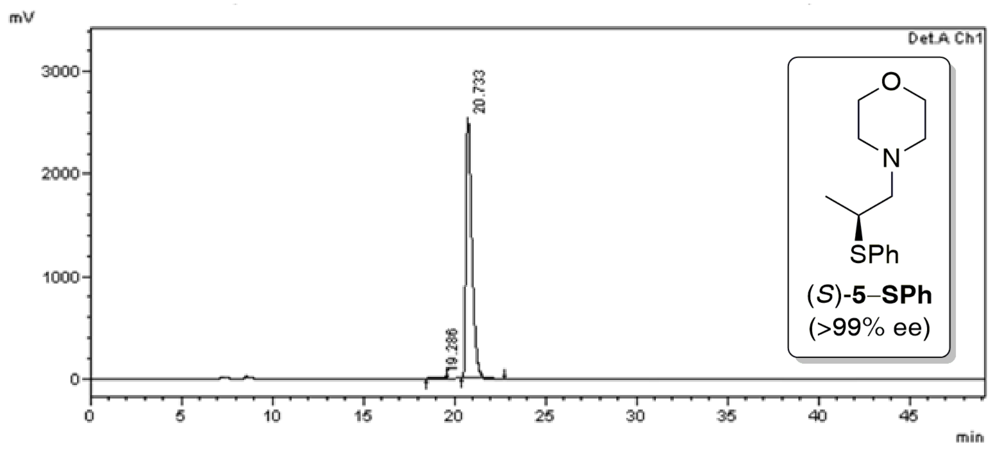

HPLC of (*R*)-5–SPh [obtained from (*S*)-(+)-5] on Chiralcel OJ-H at 30 °C

Conditions: *n*-hexane/*tert*-ButOH/Et_3N (96.5:3.0:0.5, v/v/v); f=1.0 mL/min; λ=254 nm

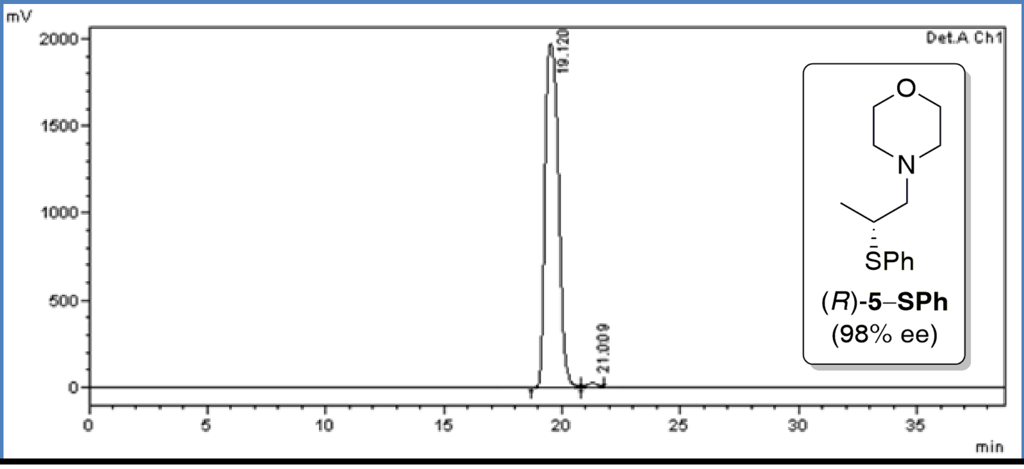

HPLC of *rac*-10 on Chiralcel OD-H at 30 °C

Conditions: *n*-hexane-2-PrOH (90:10, v/v); f=0.8 mL/min; λ=222 nm

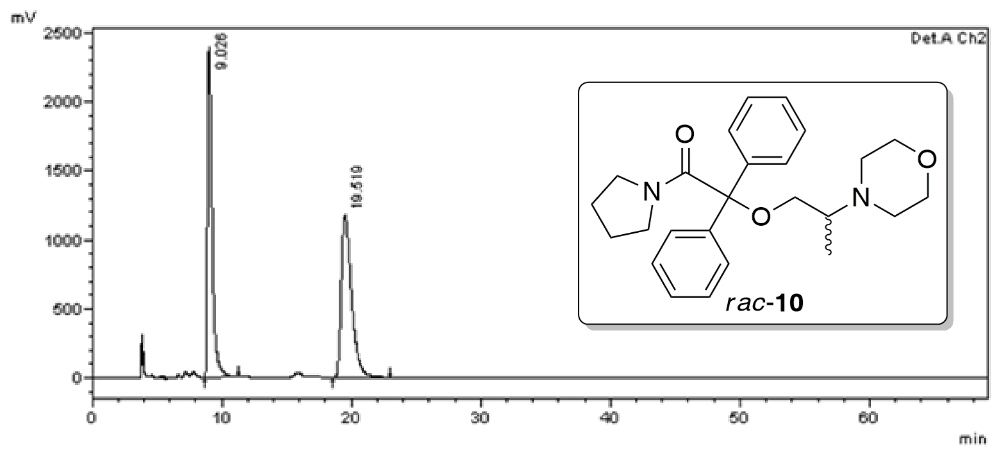

HPLC of (*S*)-(+)-10 on Chiralcel OD-H at 30 °C

Conditions: *n*-hexane-2-PrOH (90:10, v/v); f=0.8 mL/min; λ=222 nm

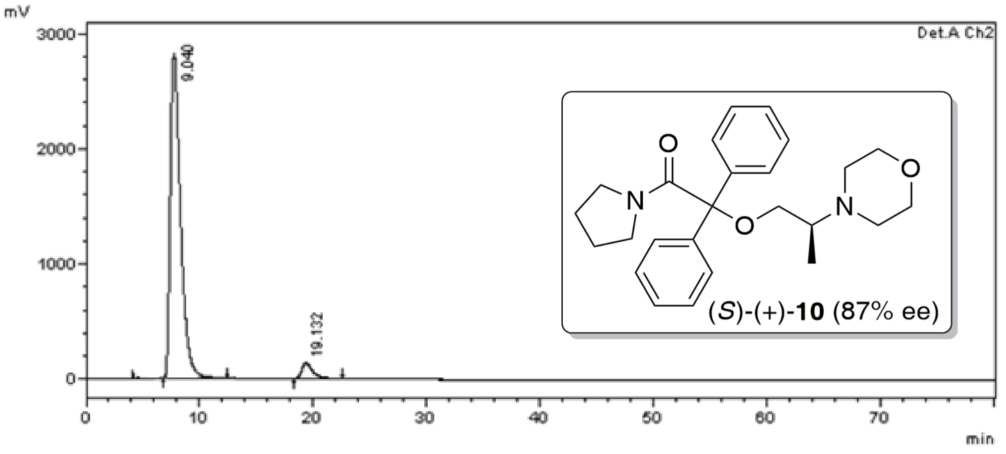

HPLC of (*R*)-(–)-10 on Chiralcel OD-H at 30 °C

Conditions: *n*-hexane-2-PrOH (90:10, v/v); f=0.8 mL/min; λ=222 nm

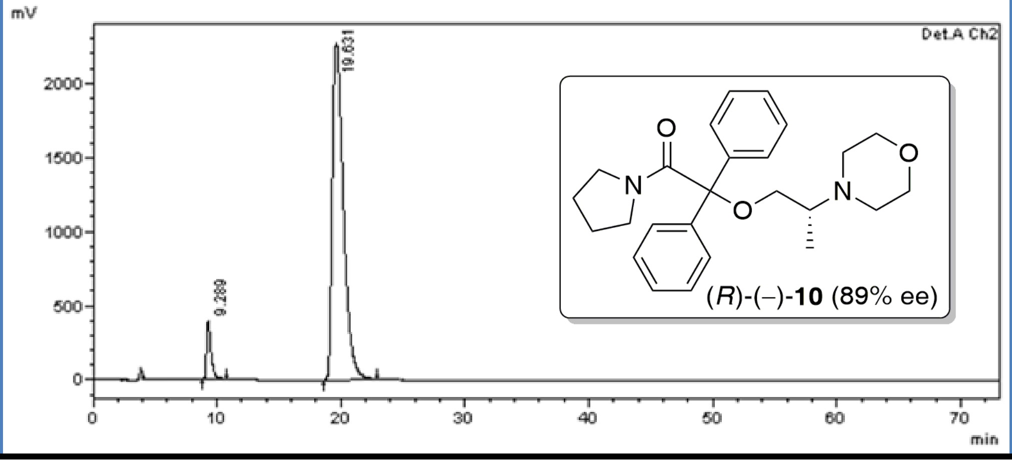

1-(Morpholin-4-yl)propan-2-ol (*rac*-**3**)

^{1}H NMR spectrum of *rac*-**3** (400 MHz, $CDCl_3$)

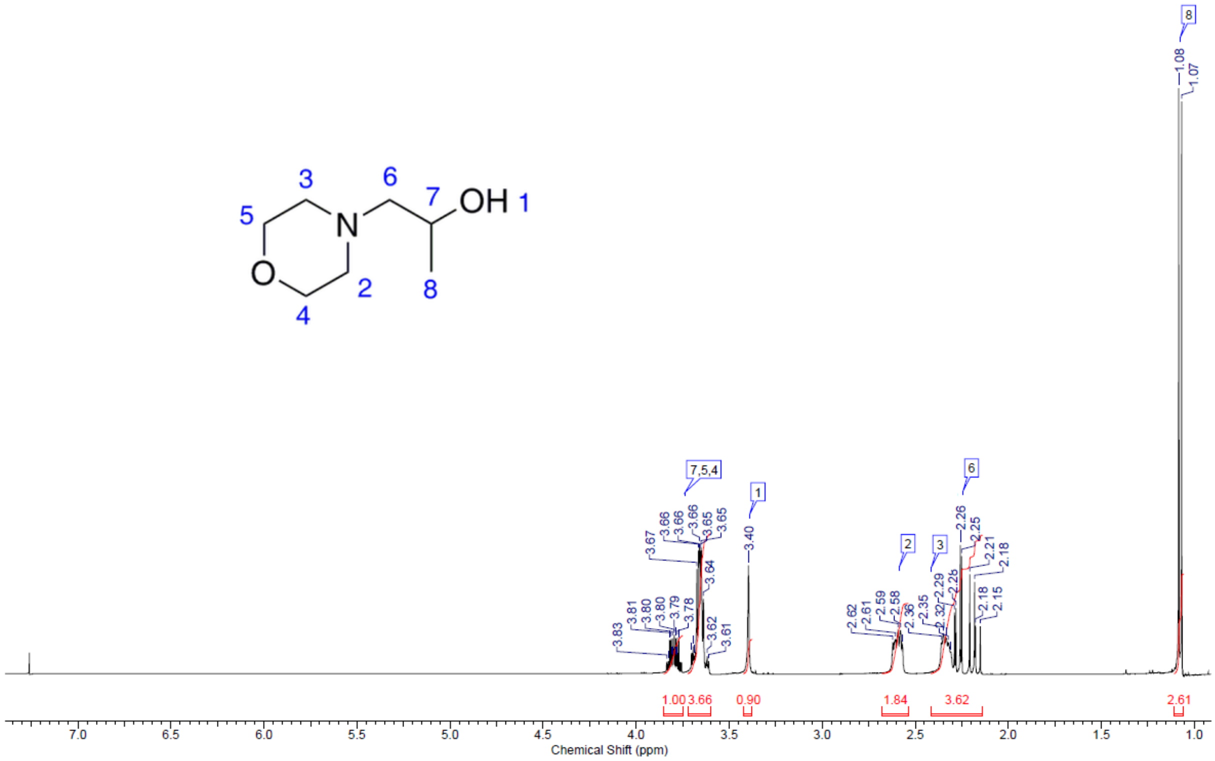

^{13}C NMR spectrum of *rac*-**3** (100 MHz, $CDCl_3$)

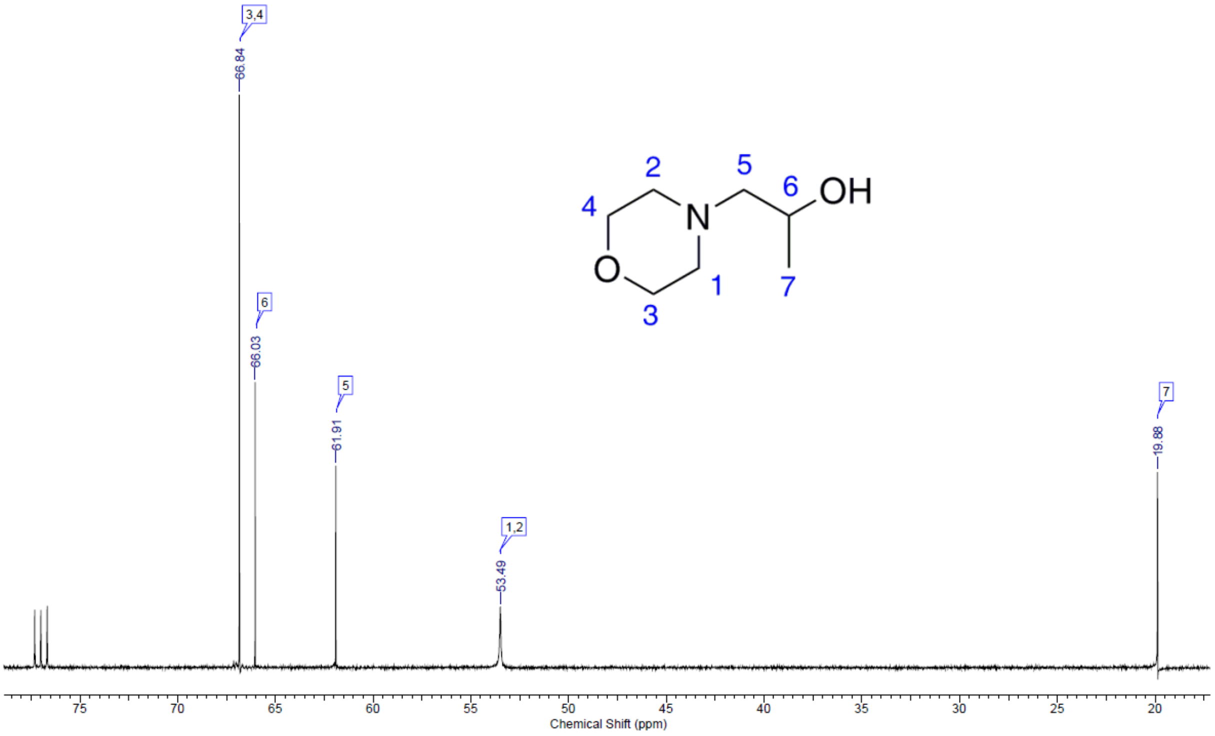

MS spectrum of *rac*-**3** (ESI-TOF)

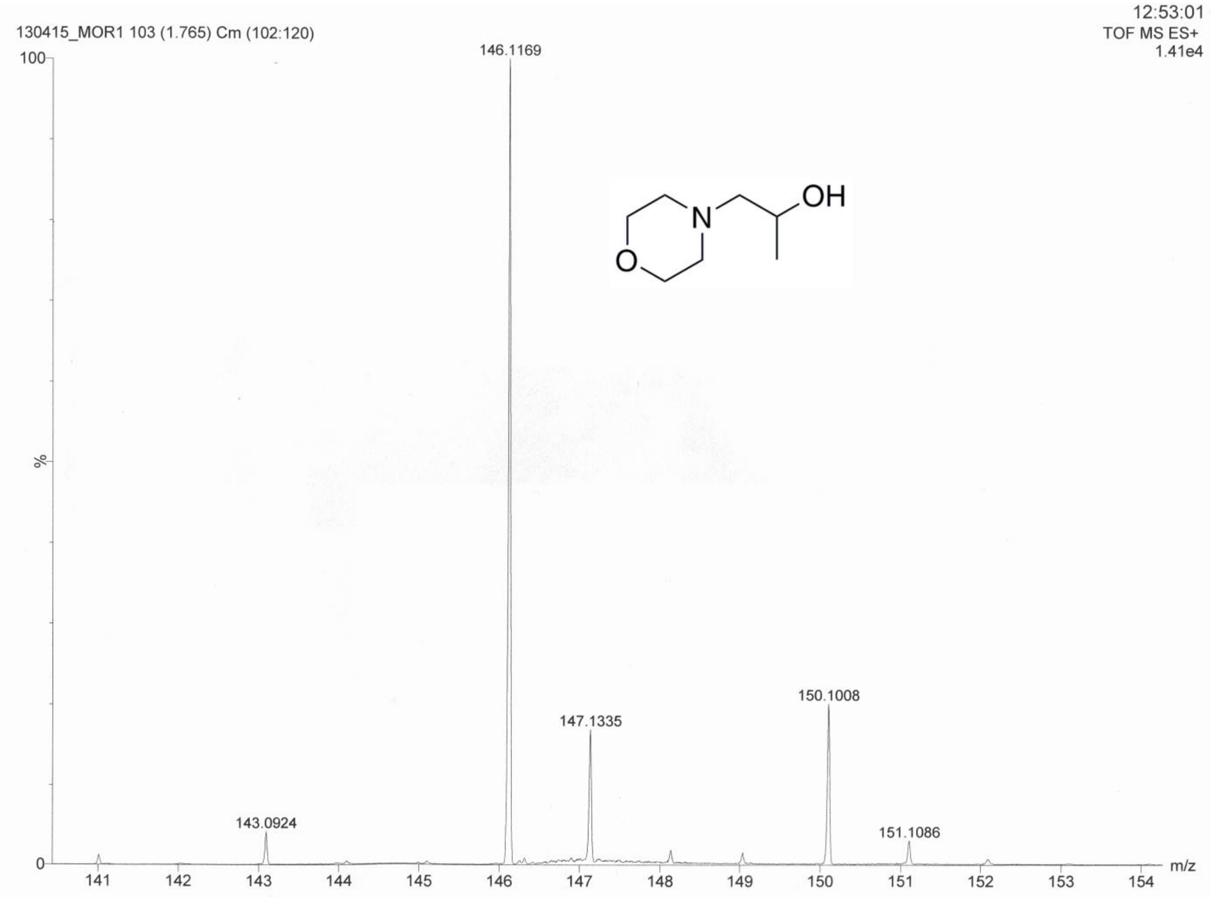

FT-IR spectrum of *rac*-**3** (neat)

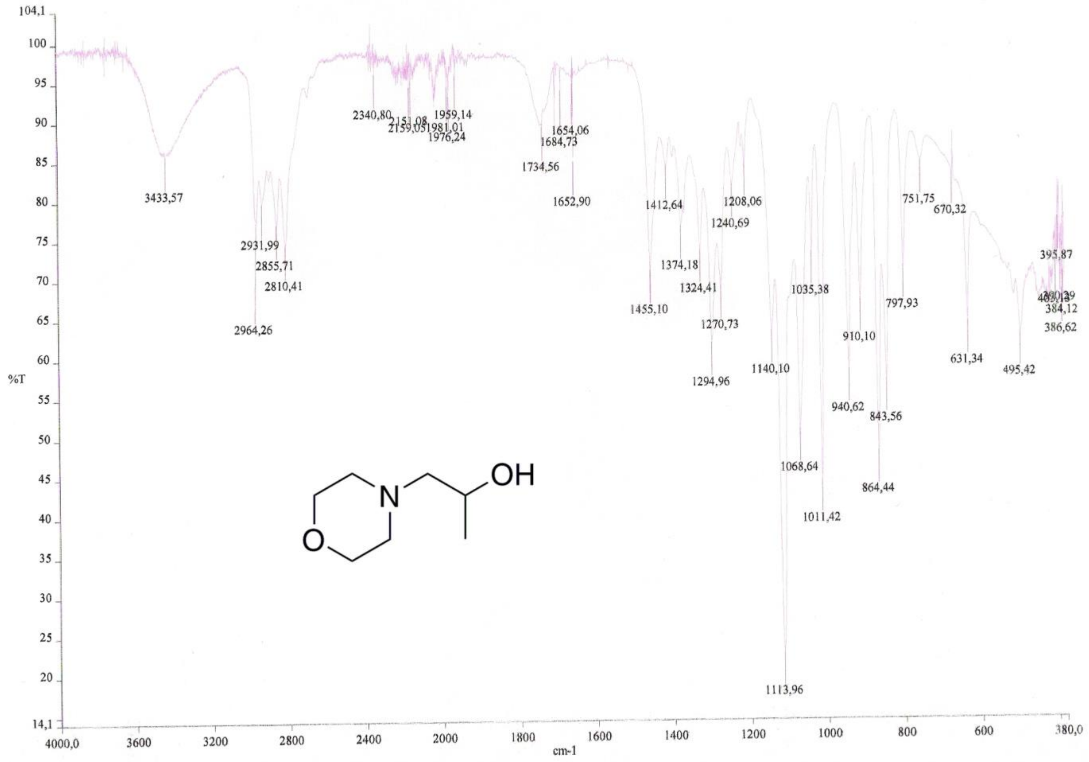

1-(Morpholin-4-yl)propan-2-yl acetate (*rac*-**4a**)

^{1}H NMR spectrum of *rac*-**4a** (400 MHz, $CDCl_3$)

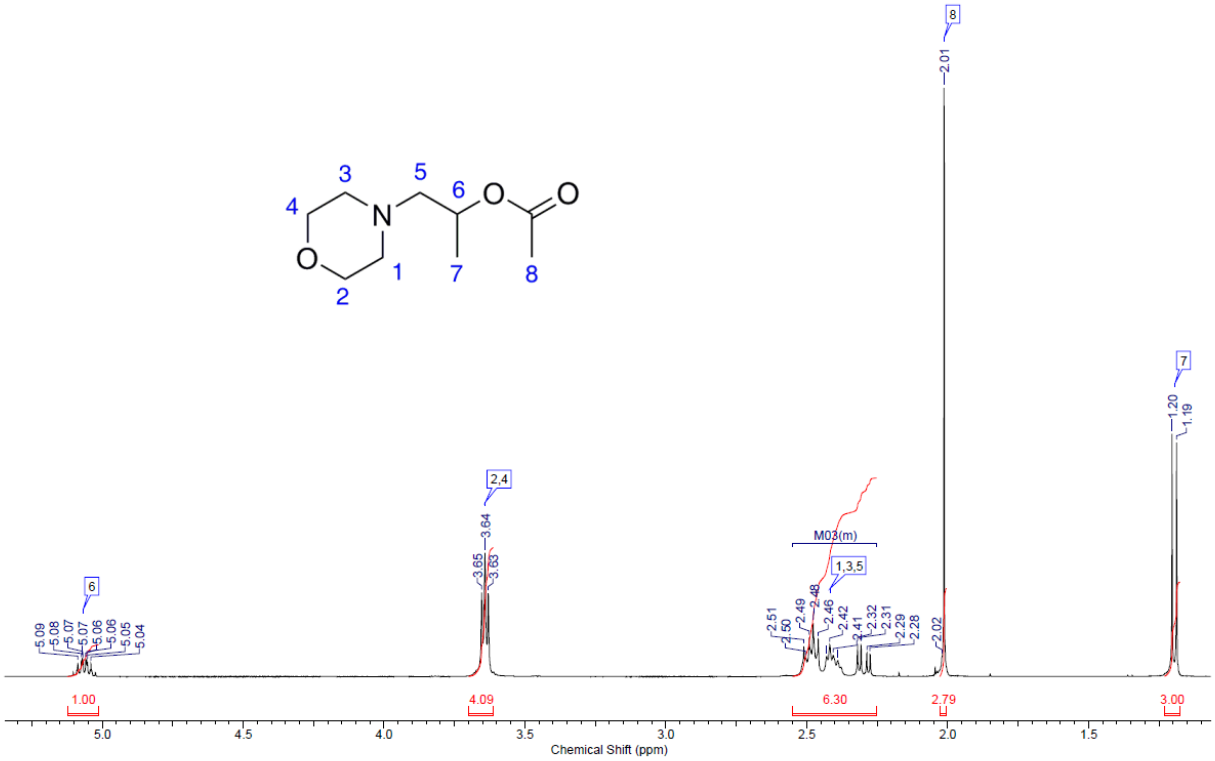

^{13}C NMR spectrum of *rac*-**4a** (100 MHz, $CDCl_3$)

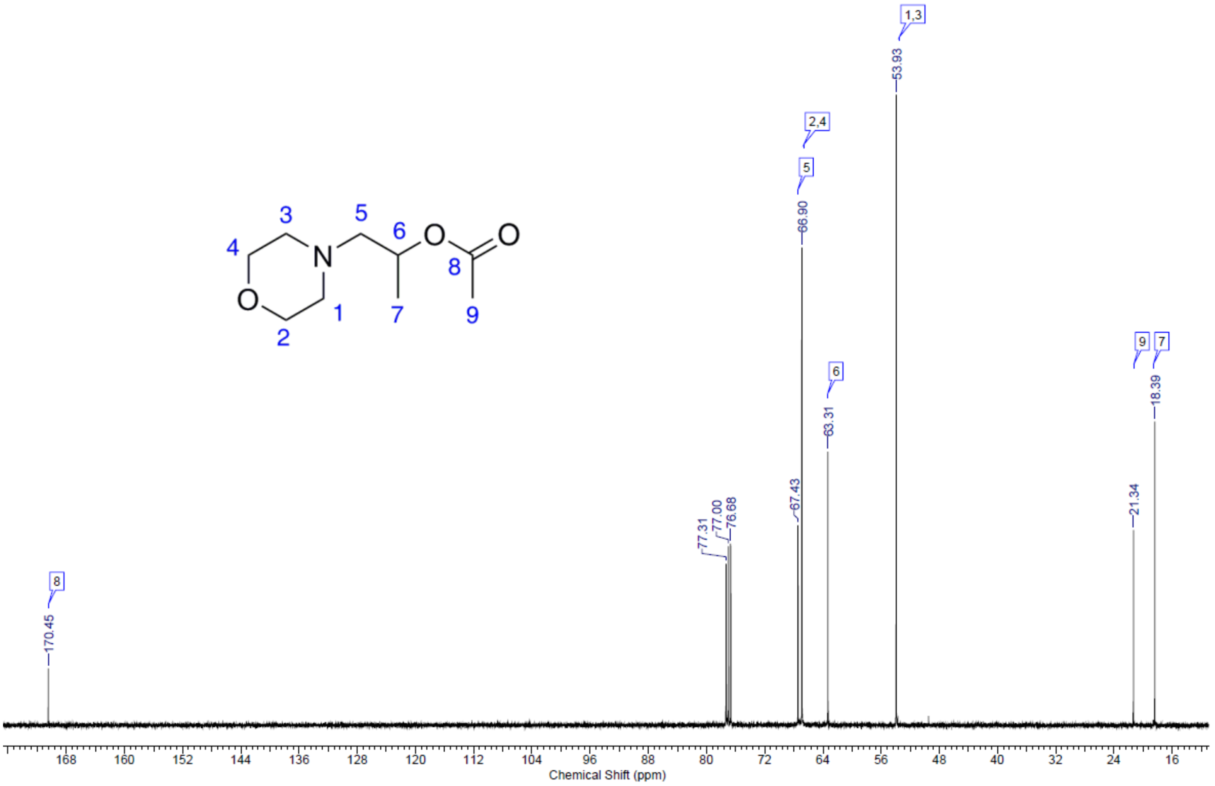

MS spectrum of *rac*-**4a** (ESI-TOF)

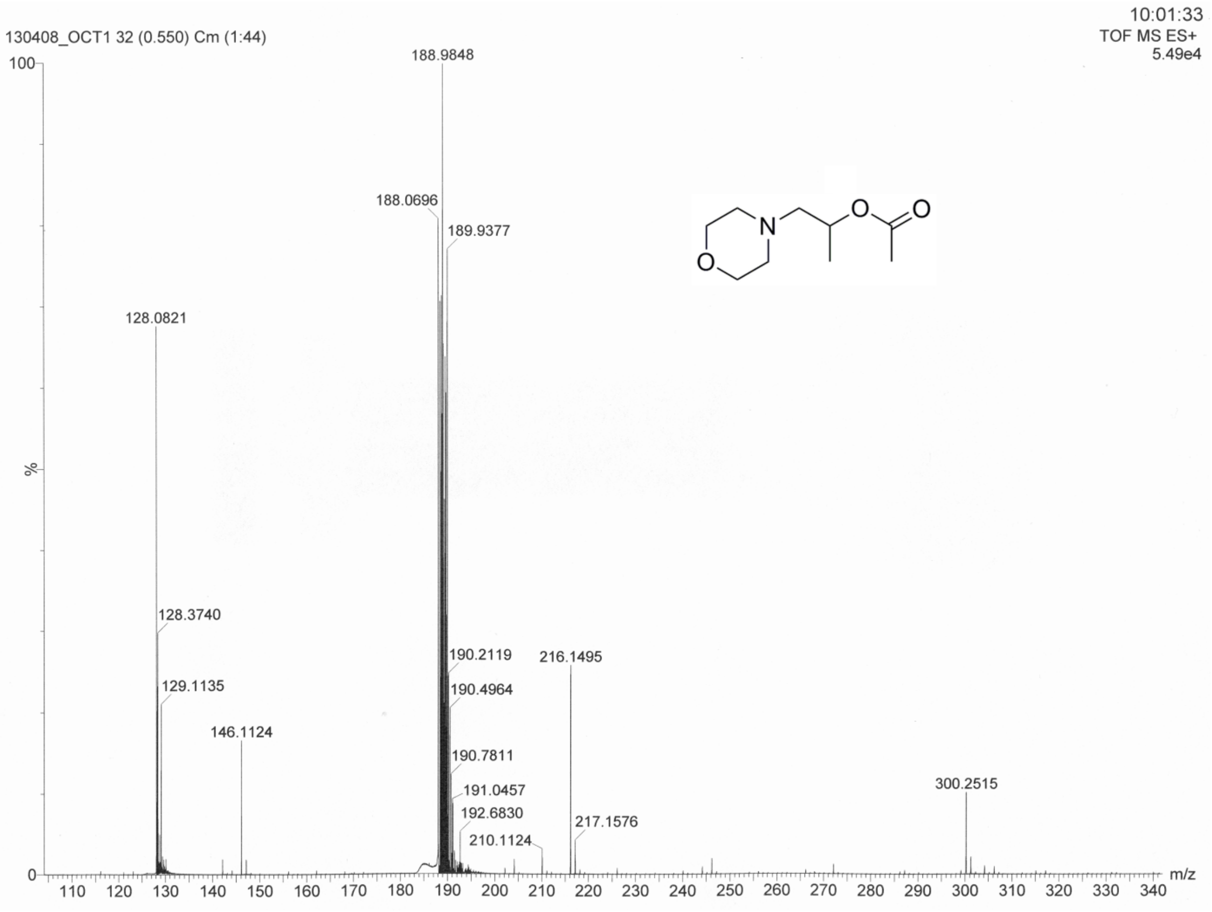

FT-IR spectrum of *rac*-**4a** (neat)

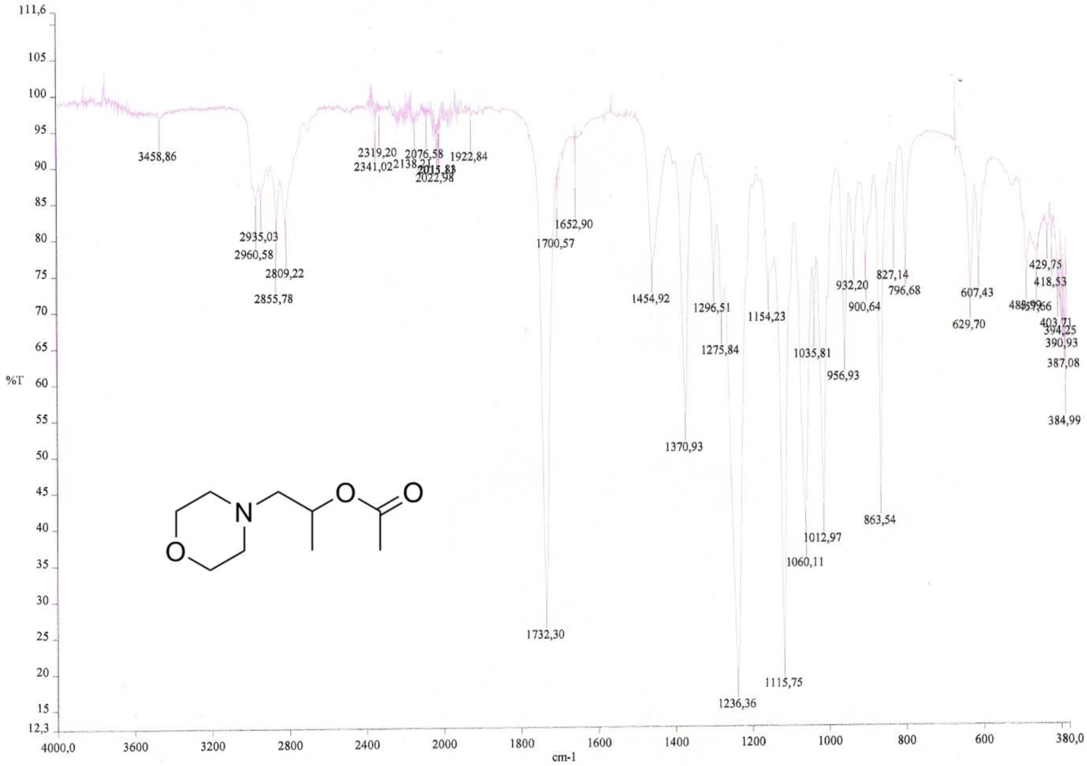

1-(Morpholin-4-yl)propan-2-yl butanoate (*rac*-**4b**)

^{1}H NMR spectrum of *rac*-**4b** (400 MHz, $CDCl_3$)

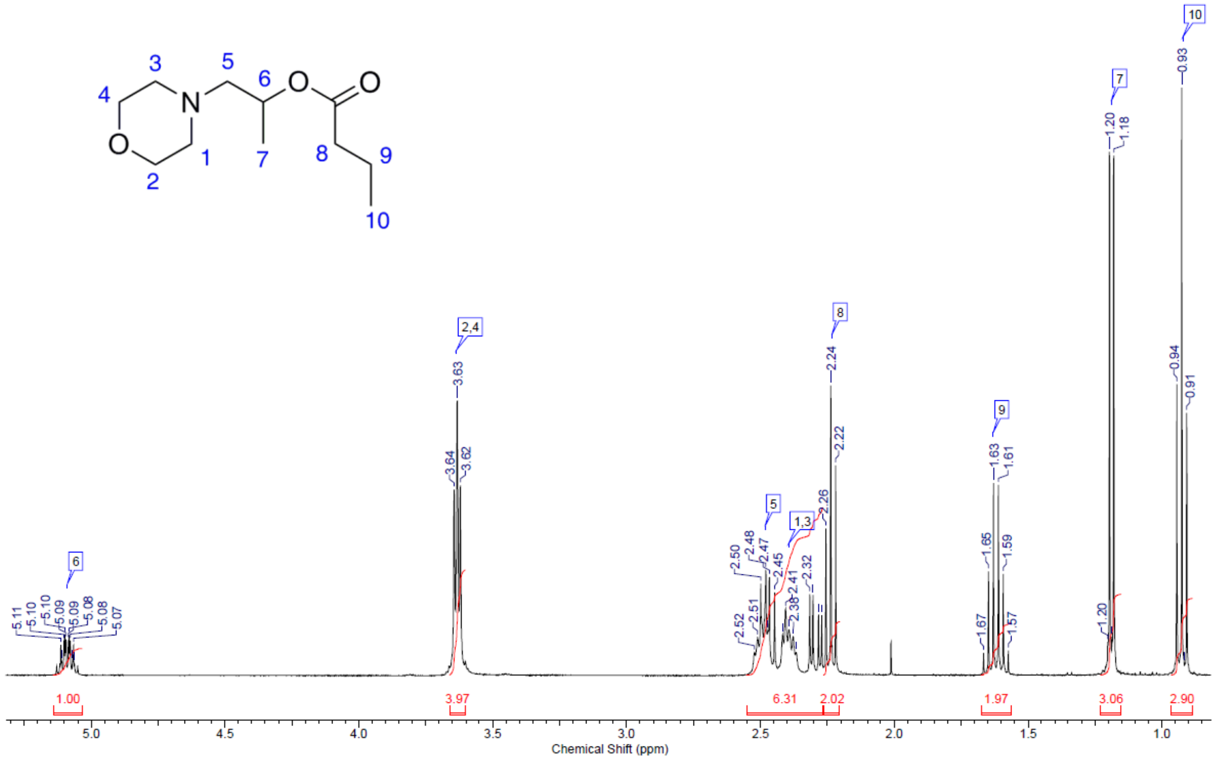

^{13}C NMR spectrum of *rac*-**4b** (100 MHz, $CDCl_3$)

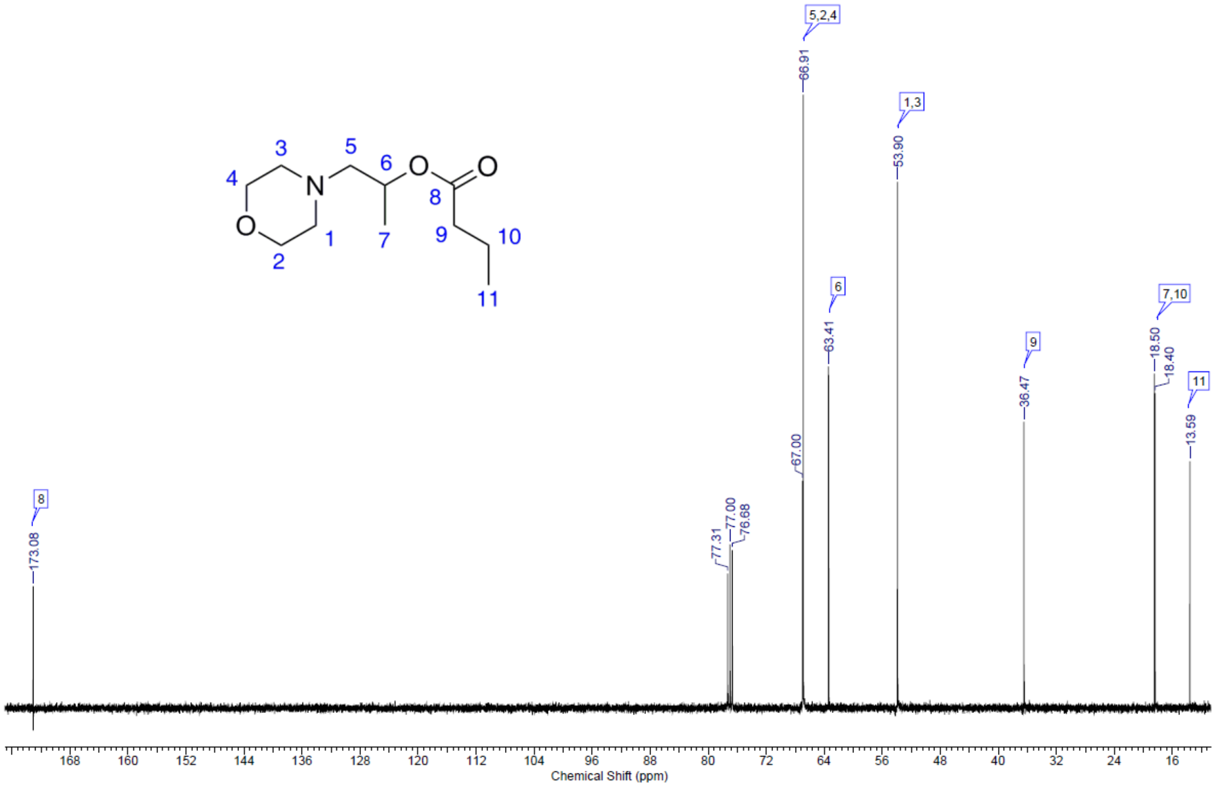

MS spectrum of *rac*-**4b** (ESI-TOF)

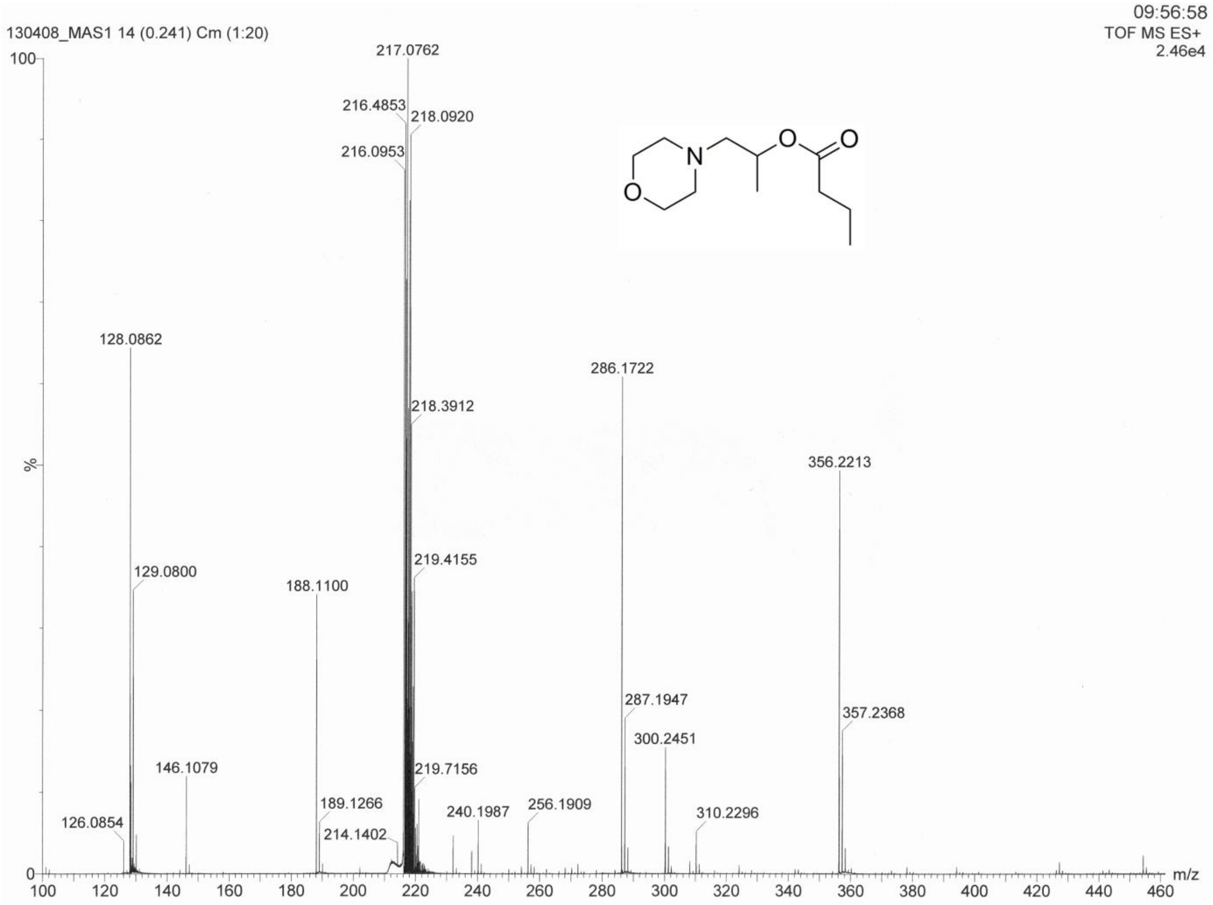

FT-IR spectrum of *rac*-**4b** (neat)

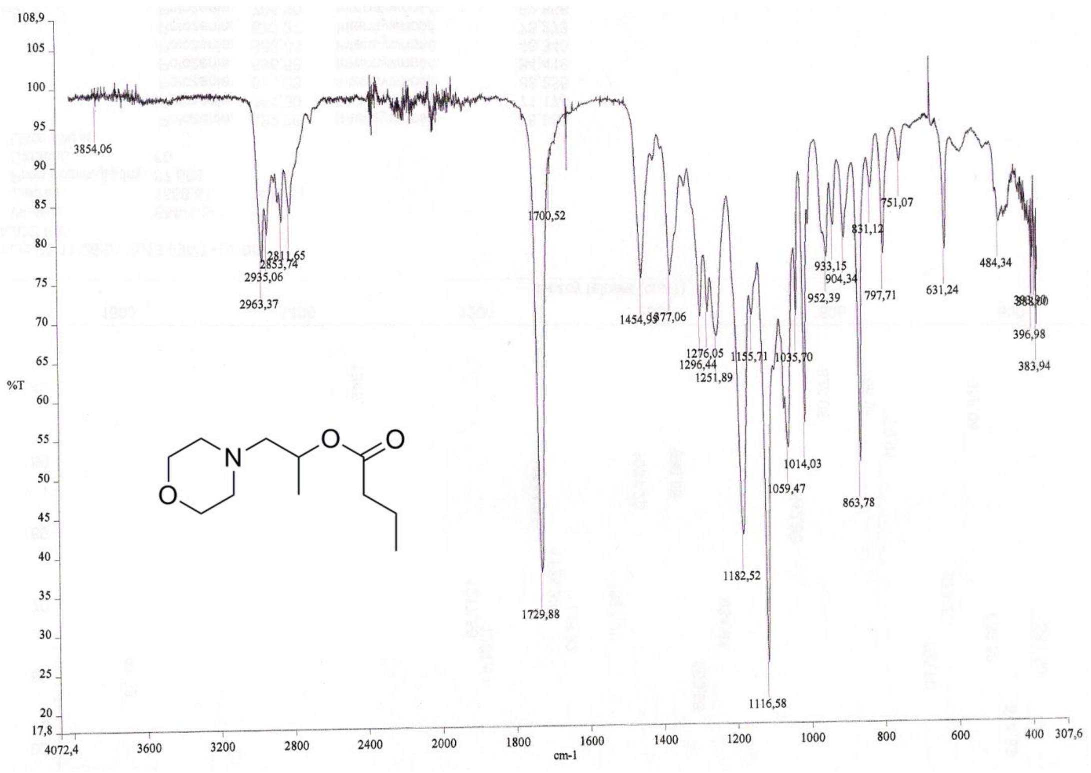

1-(Morpholin-4-yl)propan-2-yl decanoate (*rac*-**4c**)

^{1}H NMR spectrum of *rac*-**4c** (400 MHz, $CDCl_3$)

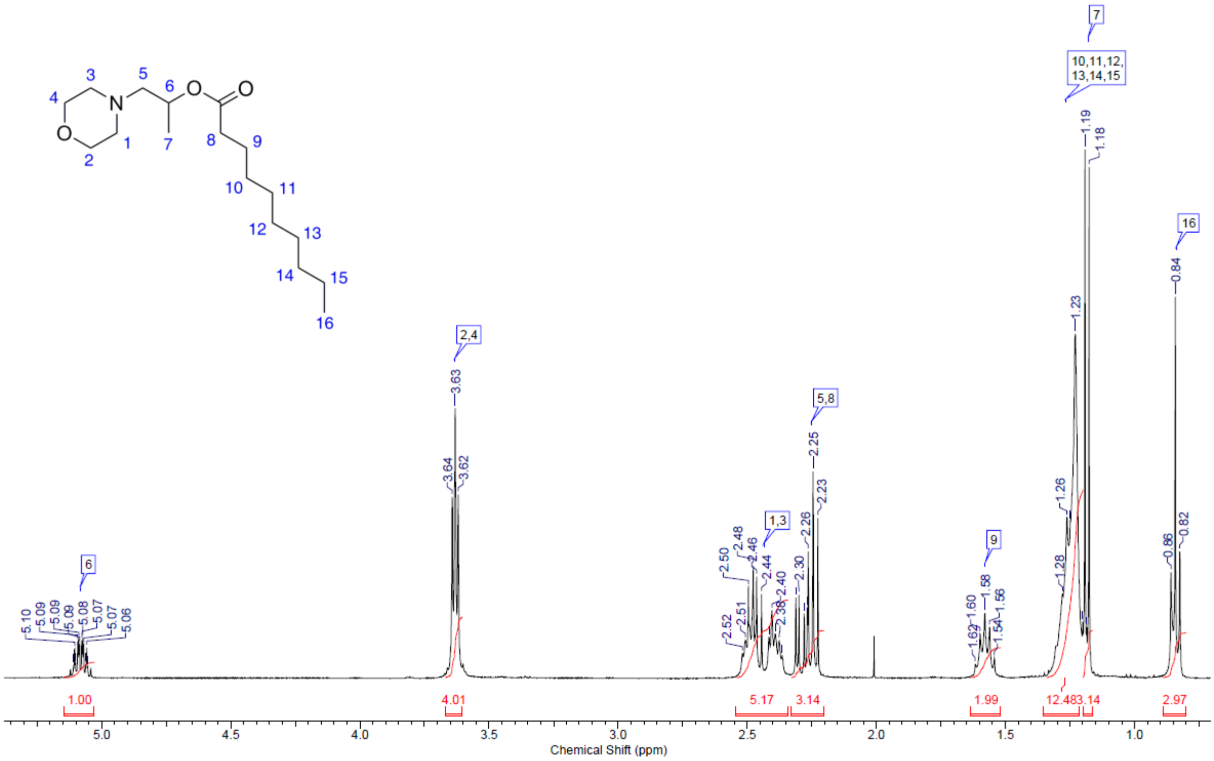

^{13}C NMR spectrum of *rac*-**4c** (100 MHz, $CDCl_3$)

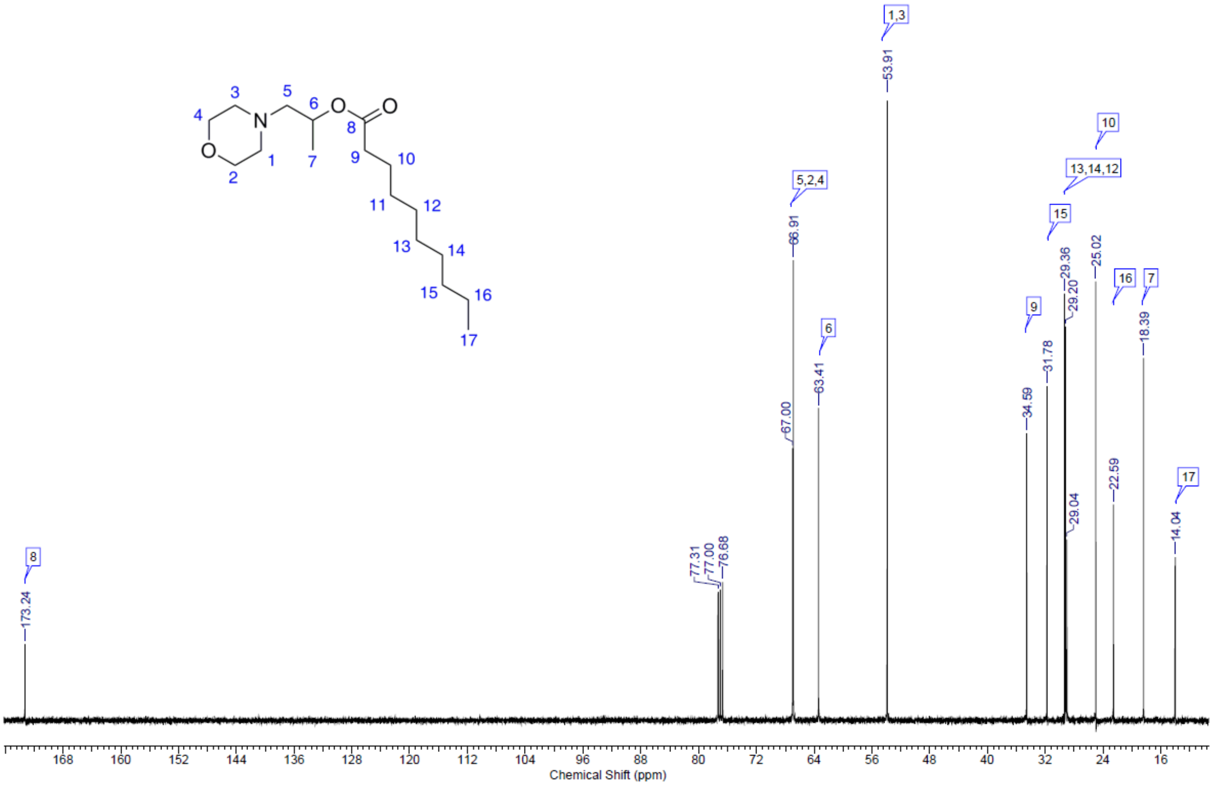

MS spectrum of *rac*-**4c** (ESI-TOF)

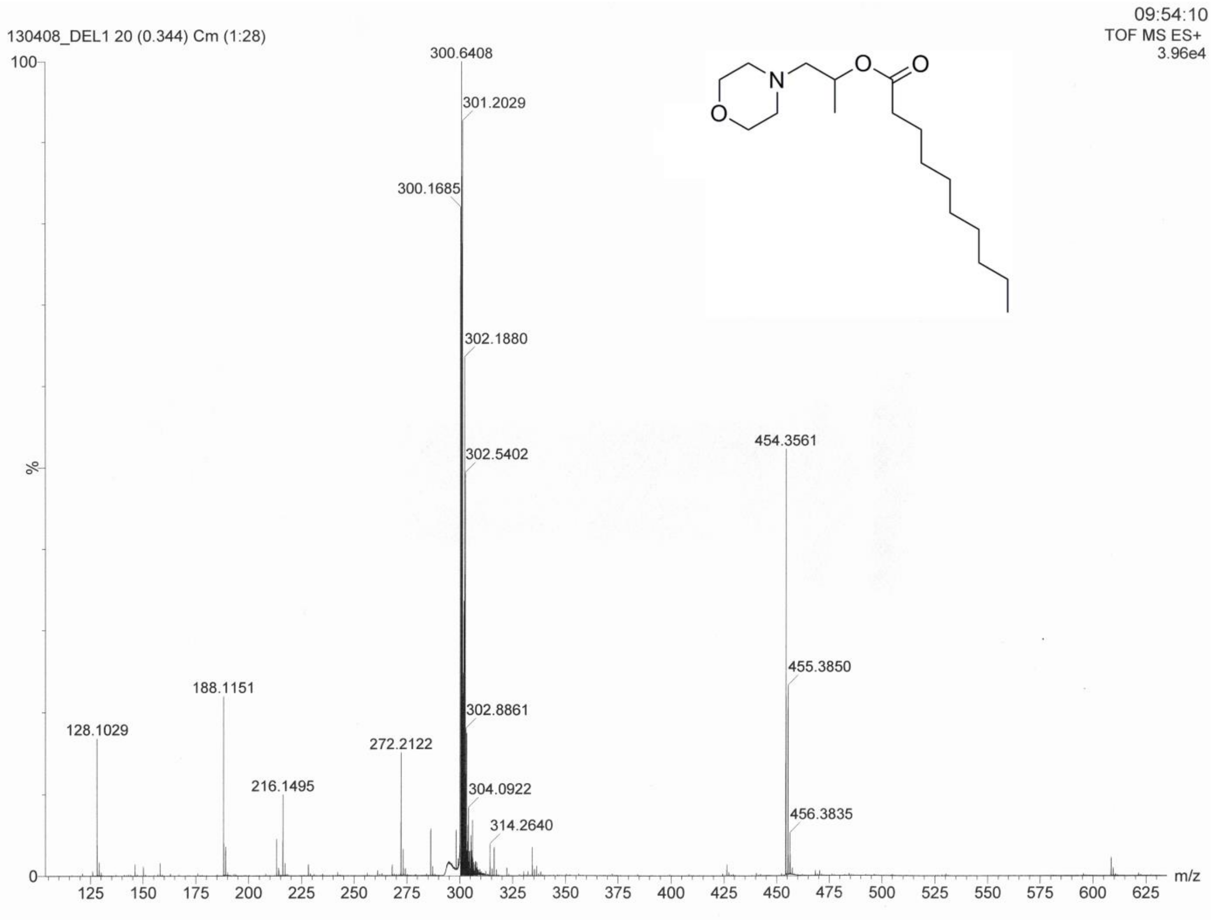

FT-IR spectrum of *rac*-**4c** (neat)

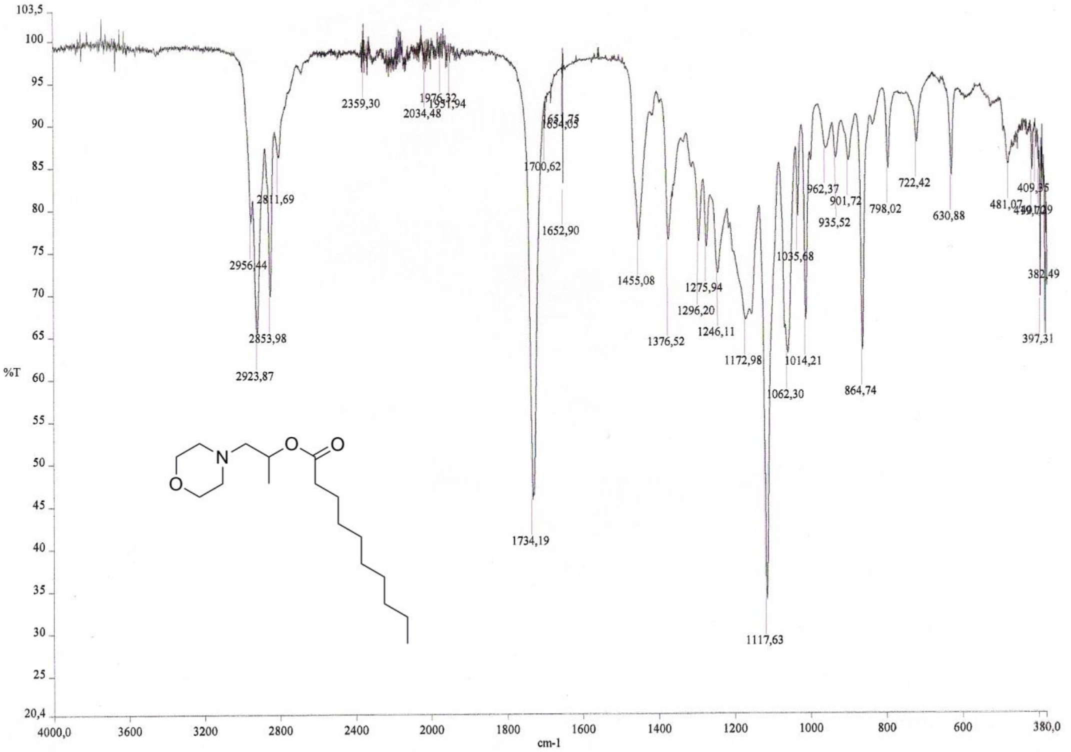

(2*S*)-1-(Morpholin-4-yl)propan-2-yl (2*R*)-methoxy(phenyl)acetate [(*S*)-(+)-**3**-(*R*)-**MPA**]

^{1}H NMR spectrum of (*S*)-(+)-**3**-(*R*)-**MPA** (400 MHz, $CDCl_3$)

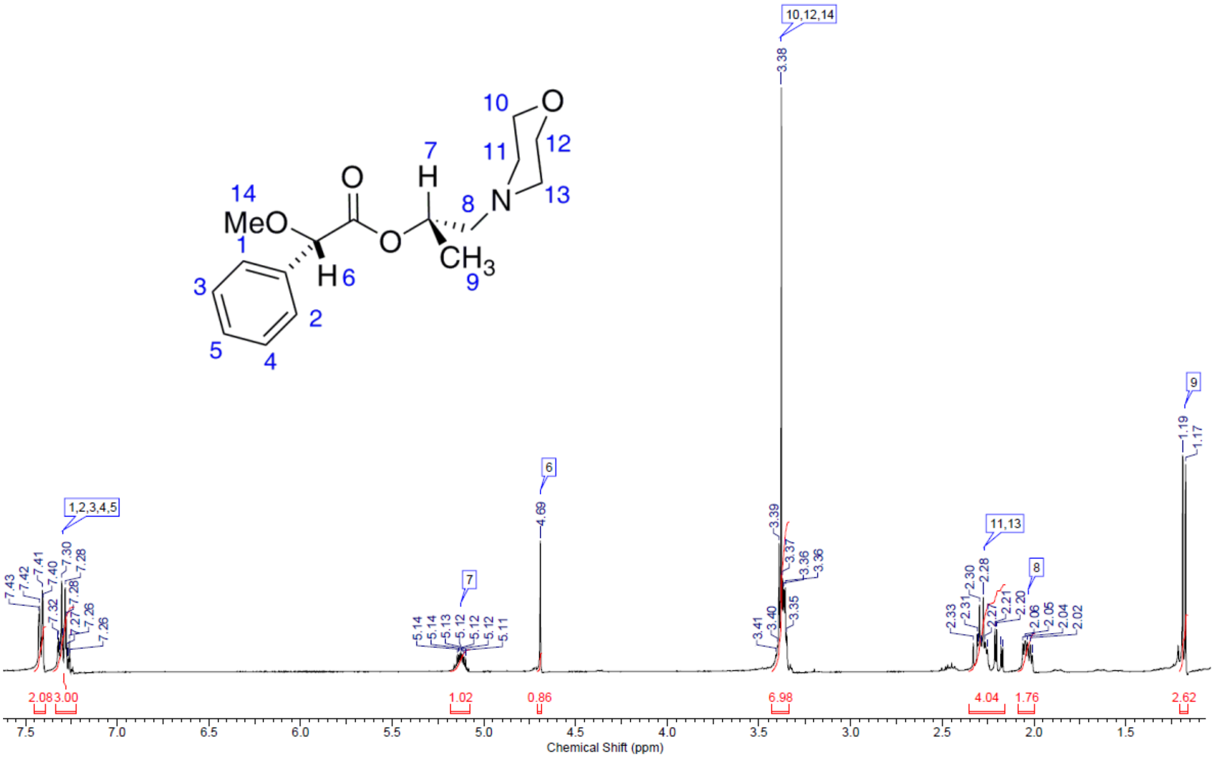

^{13}C NMR spectrum of (*S*)-(+)-**3**-(*R*)-**MPA** (100 MHz, $CDCl_3$)

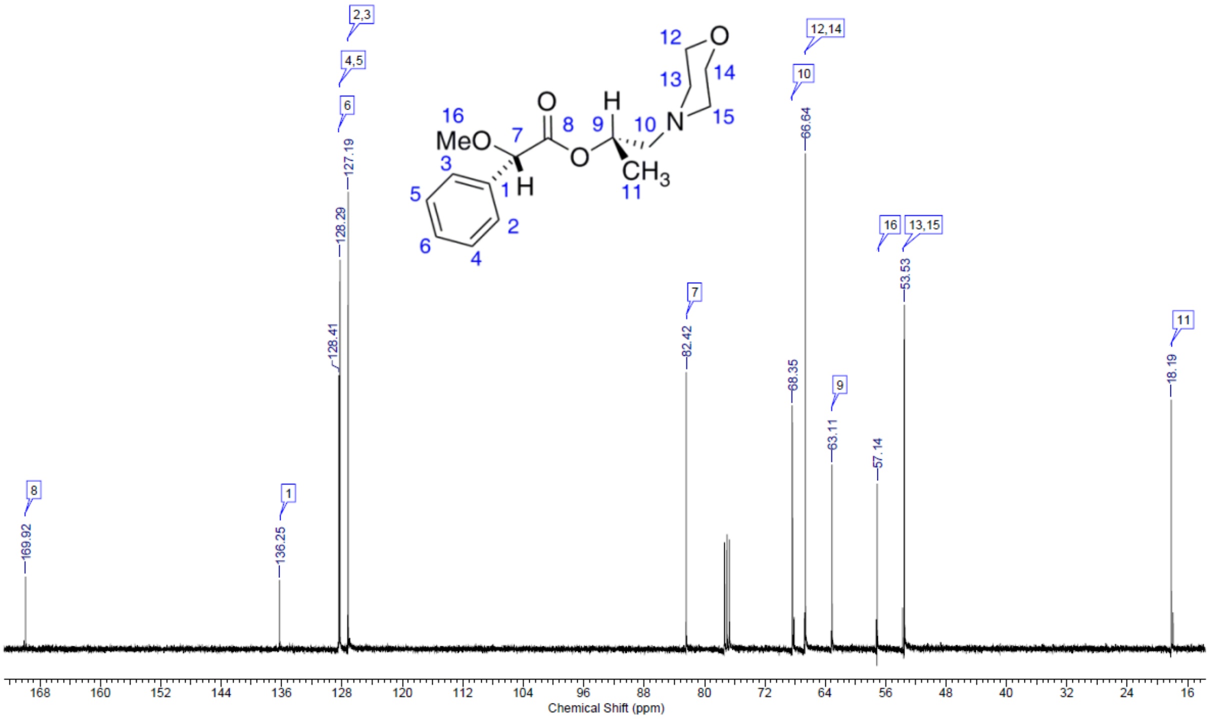

MS spectrum of (*S*)-(+)-**3**-(*R*)-**MPA** (ESI-TOF)

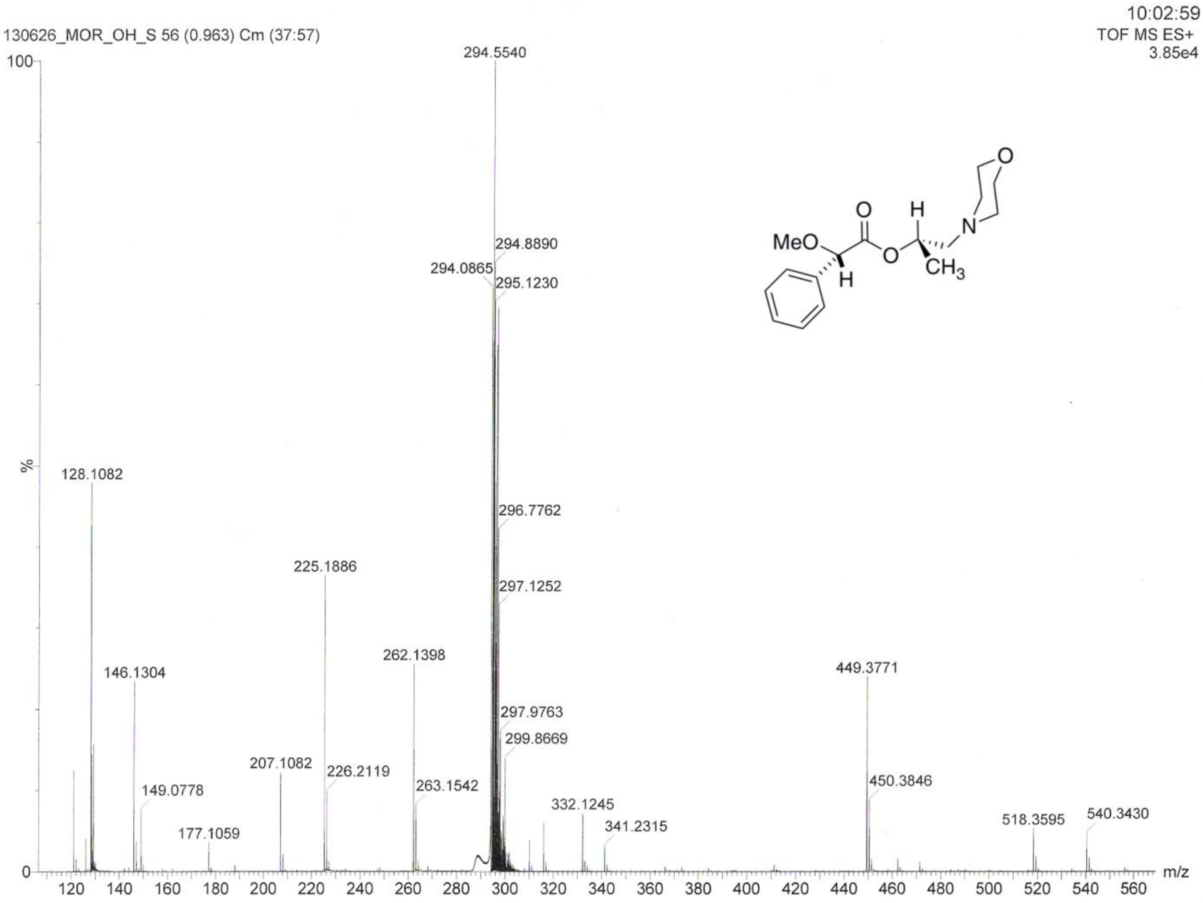

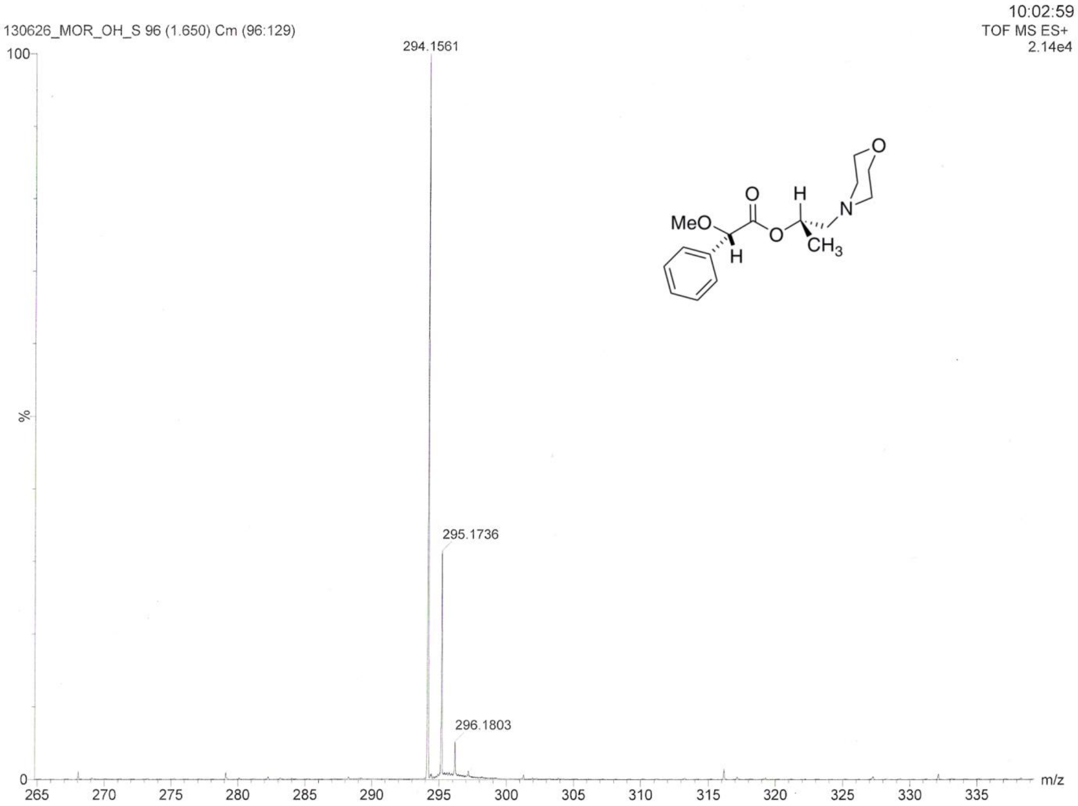

(2*S*)-1-(Morpholin-4-yl)propan-2-yl (2*S*)-methoxy(phenyl)acetate [(*S*)-(+)-**3**-(*R*)-**MPA**]

^{1}H NMR spectrum of (*S*)-(+)-**3**-(*R*)-**MPA** (400 MHz, $CDCl_3$)

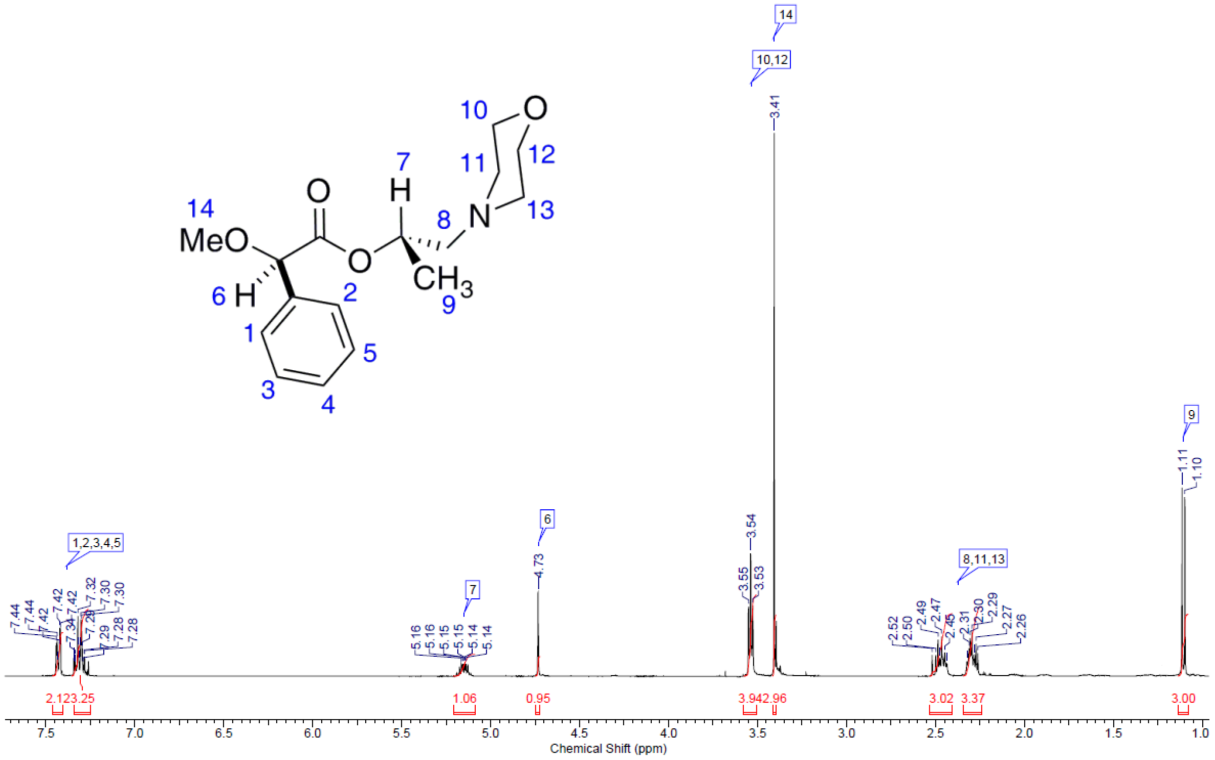

^{13}C NMR spectrum of (*S*)-(+)-**3**-(*R*)-**MPA** (100 MHz, $CDCl_3$)

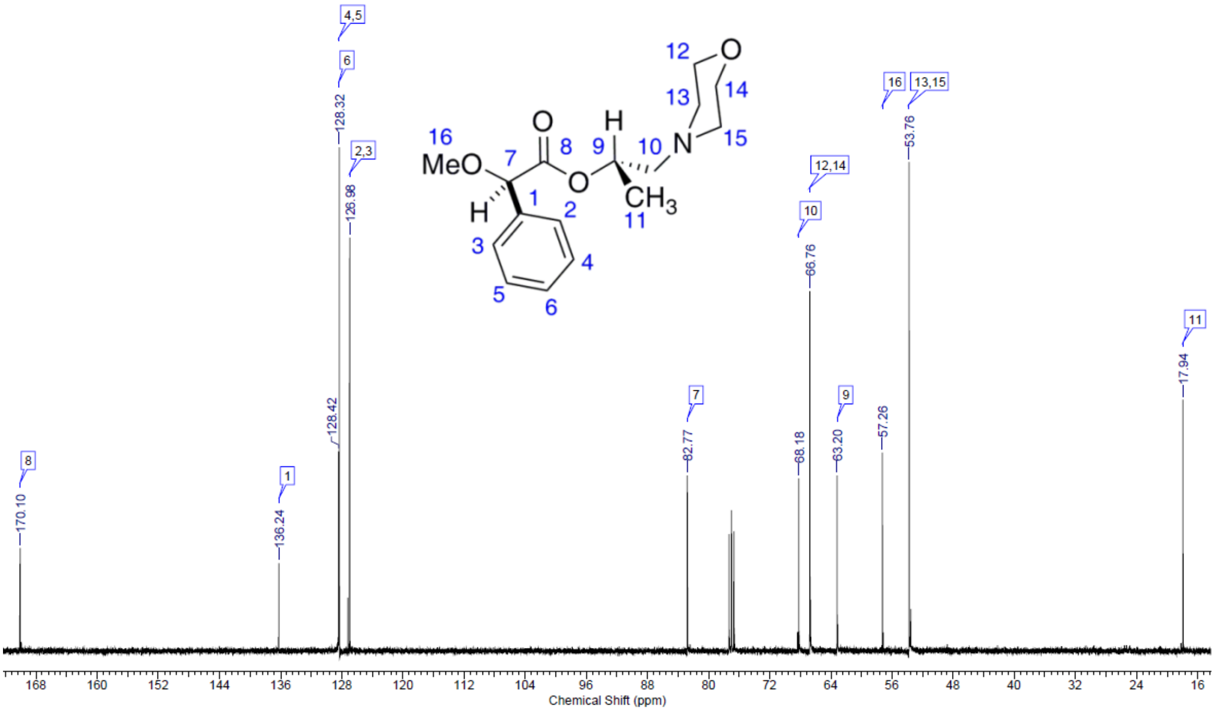

MS spectrum of (*S*)-(+)-**3**-(*R*)-**MPA** (ESI-TOF)

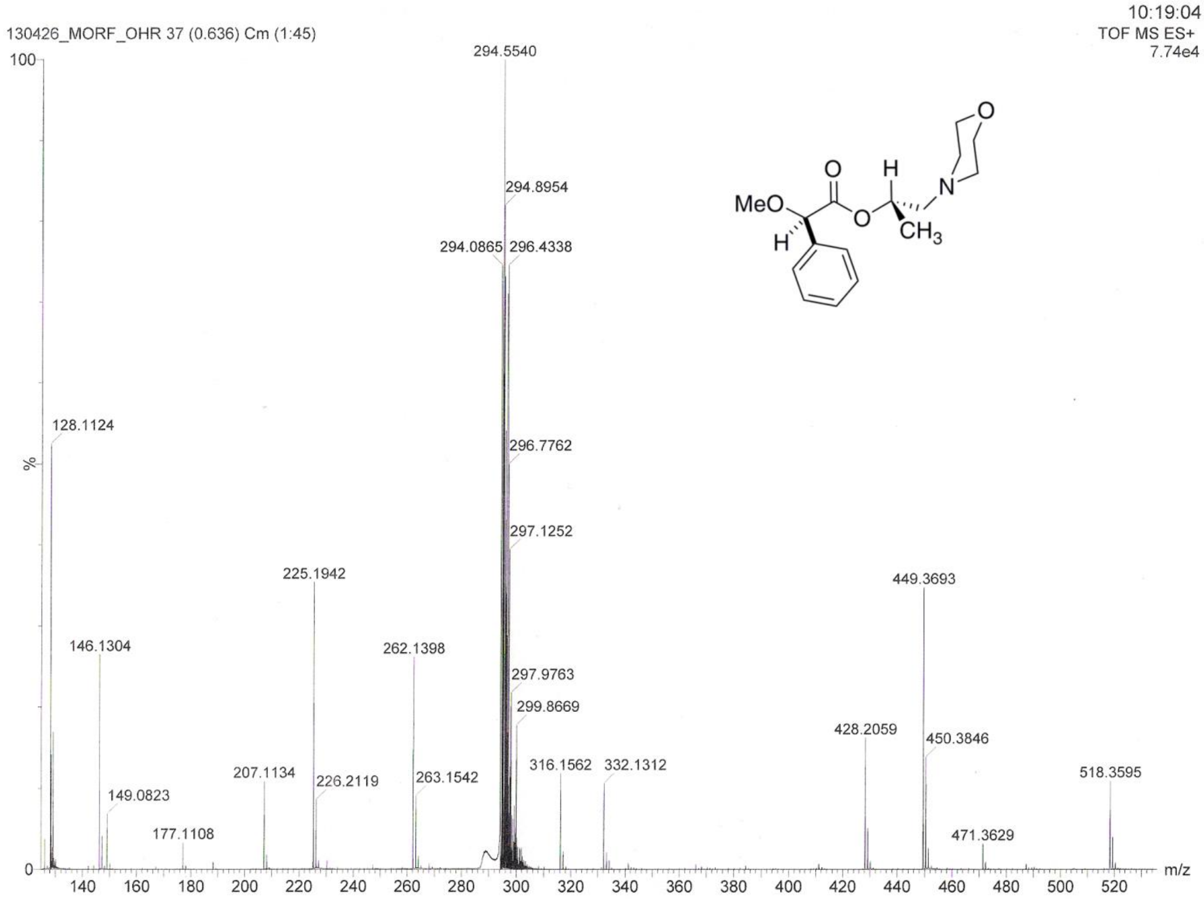

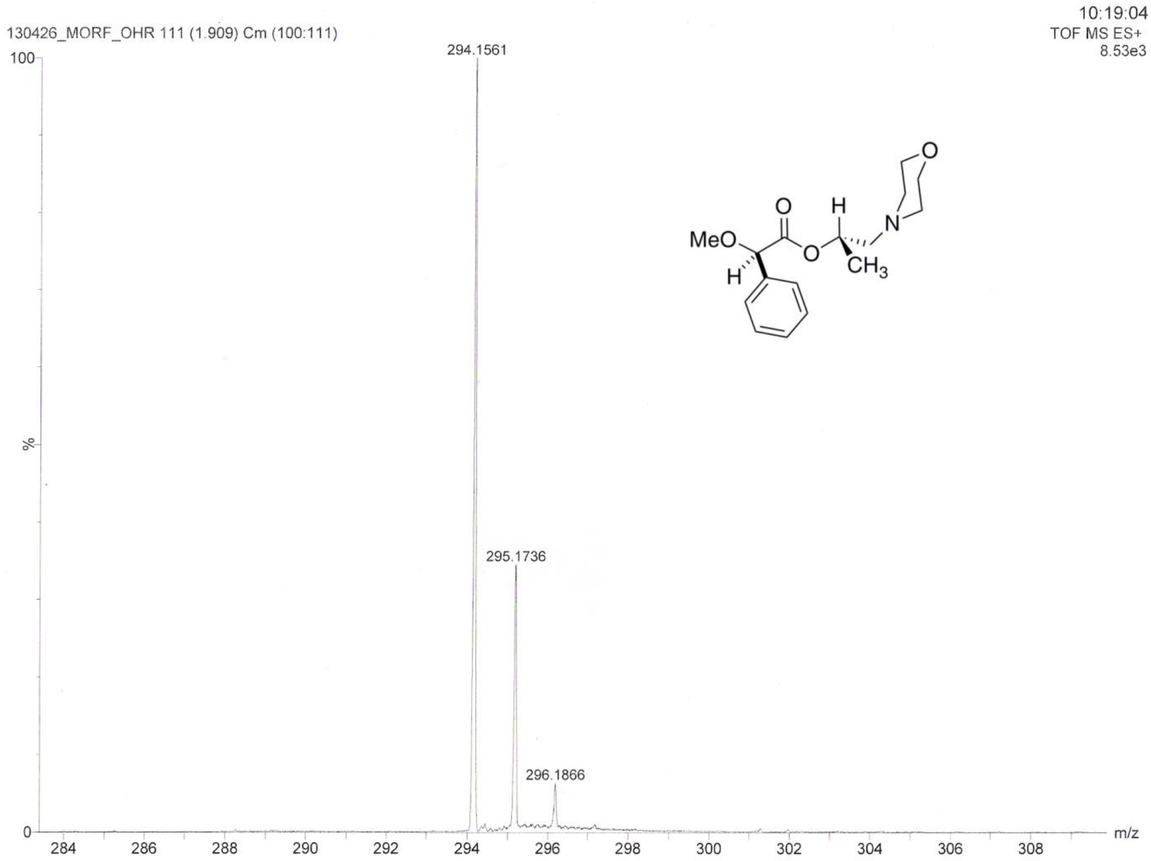

4-(2-Chloropropyl)morpholine (*rac*-**5**)

^{1}H NMR spectrum of *rac*-**5** (400 MHz, $CDCl_3$)

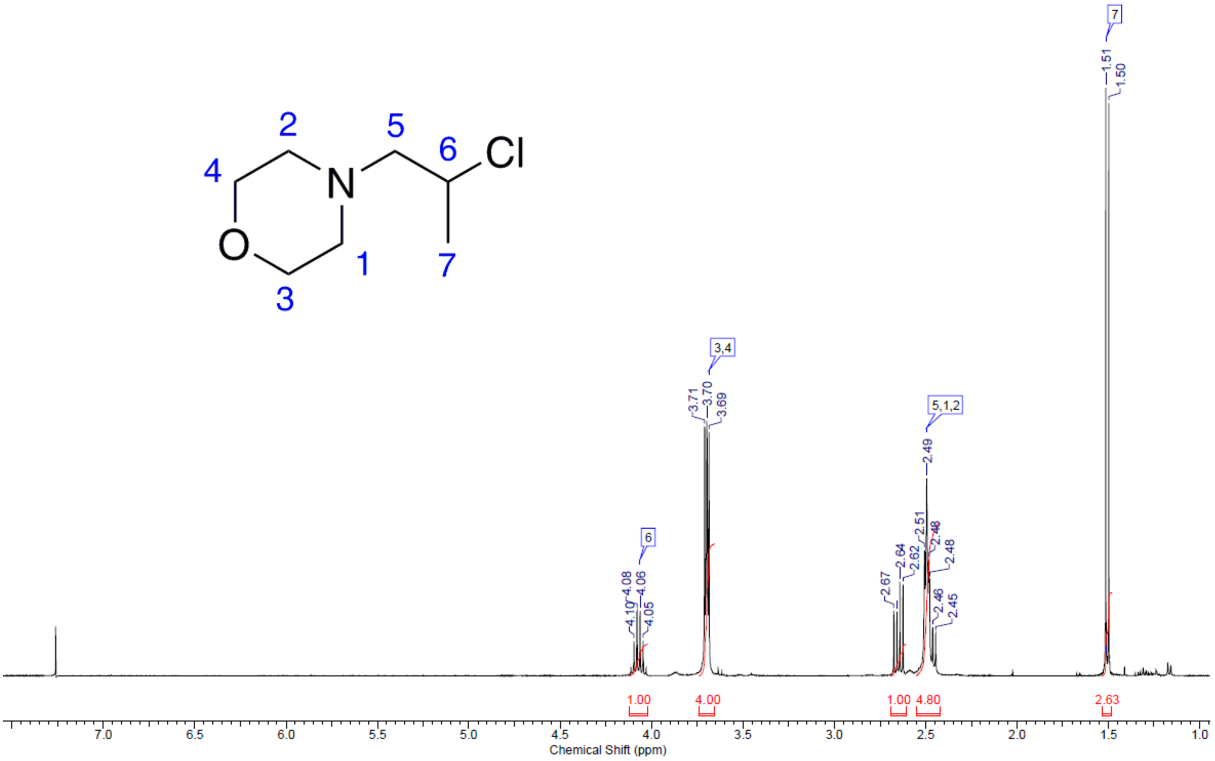

^{13}C NMR spectrum of *rac*-**5** (100 MHz, $CDCl_3$)

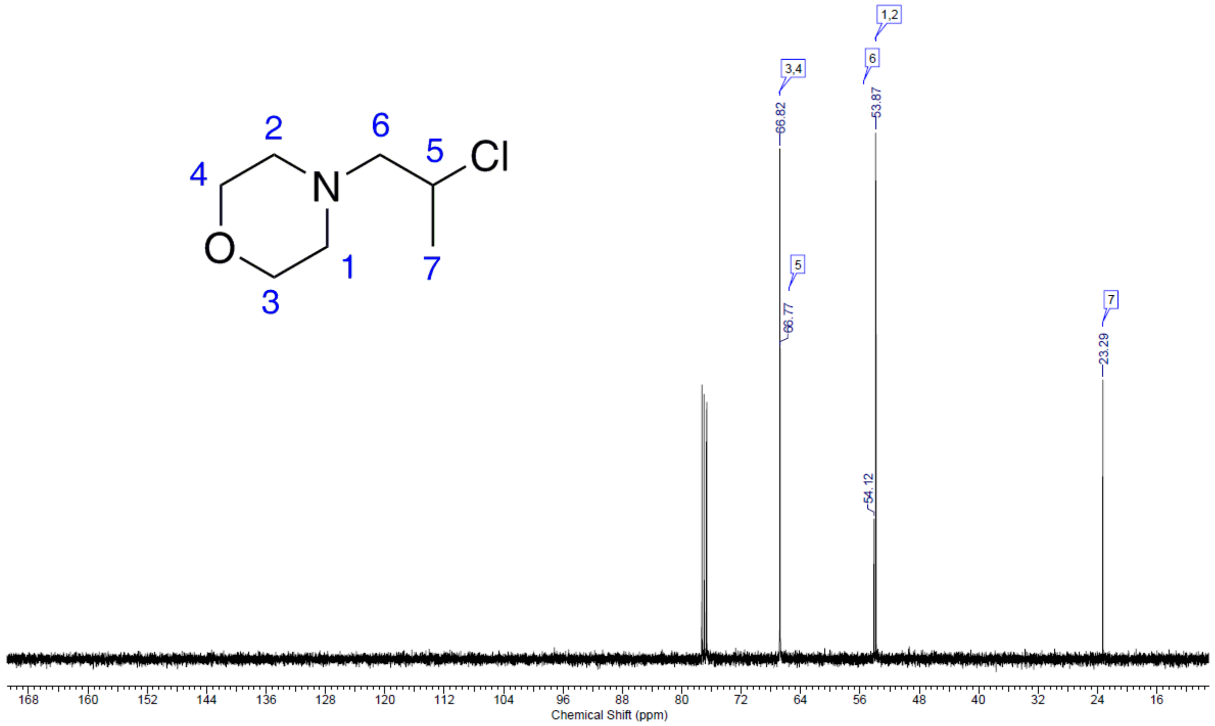

MS spectrum of *rac*-**5** (ESI-TOF)

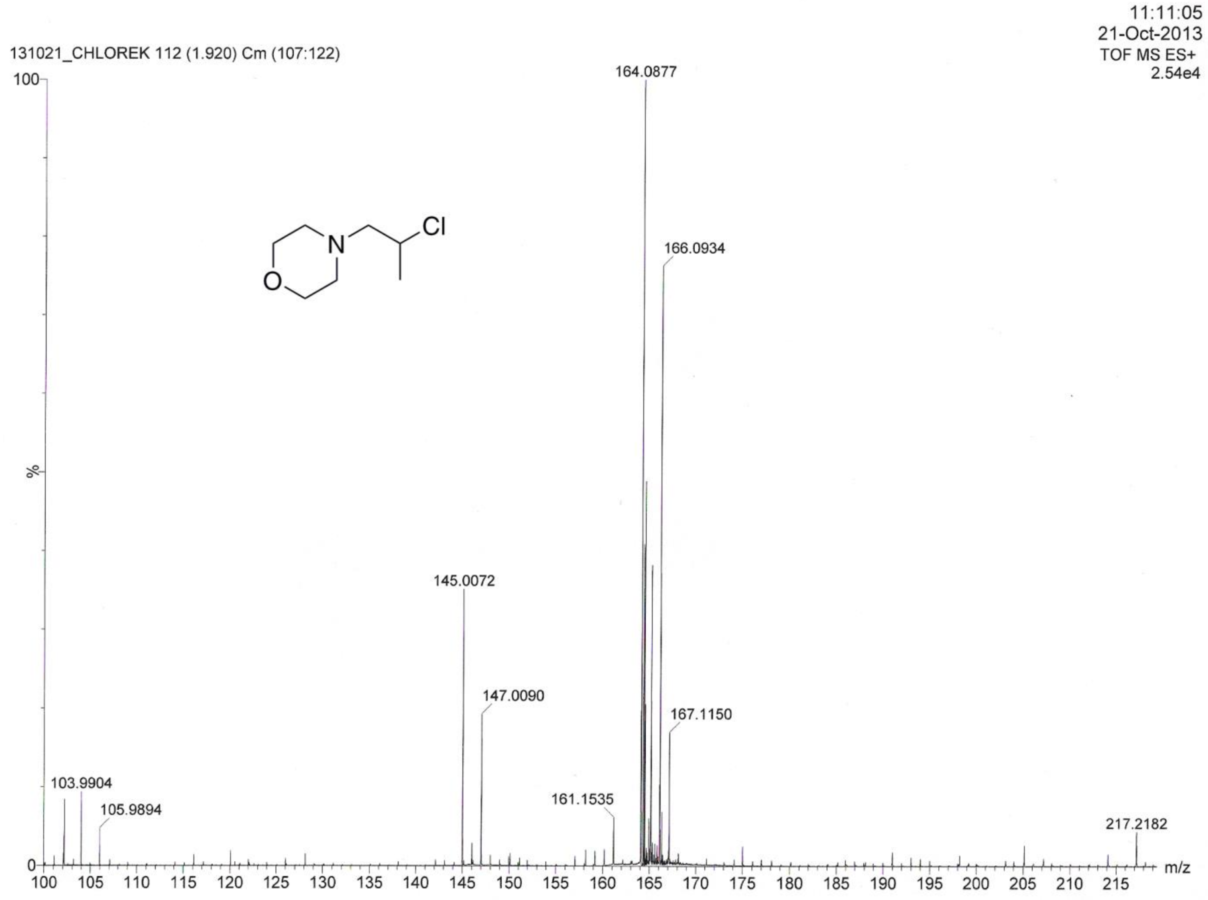

FT-IR spectrum of *rac*-**5** (neat)

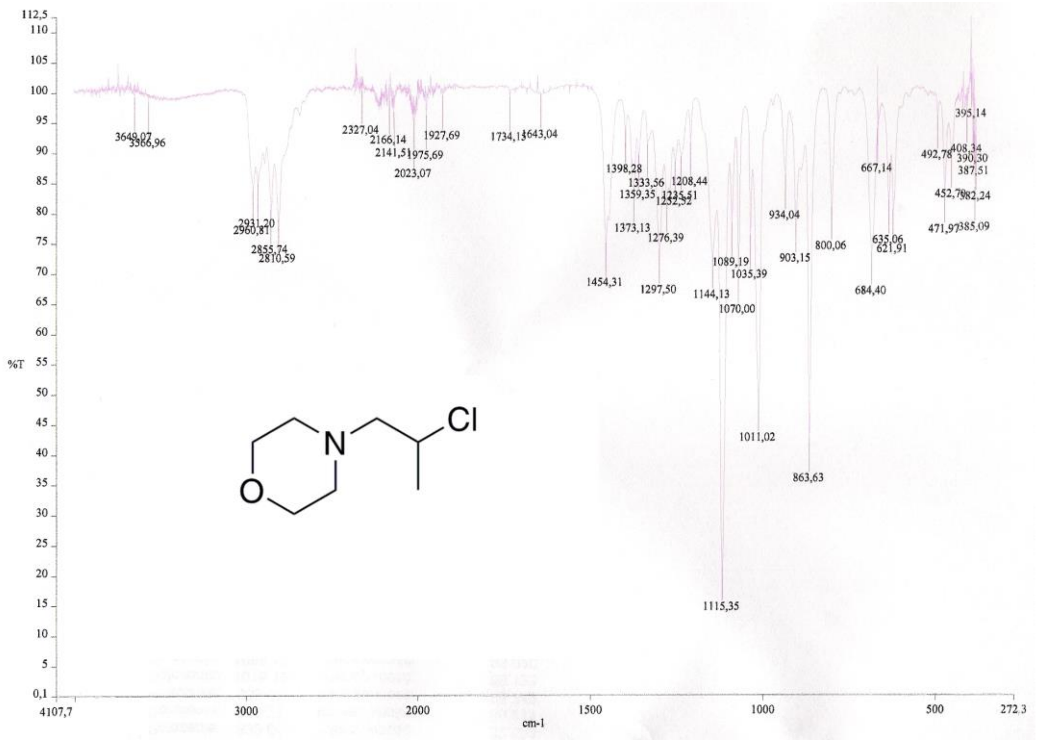

4-[2-(Phenylsulfanyl)propyl]morpholine (*rac*-**5**–**SPh**)

^{1}H NMR spectrum of *rac*-**5**–**SPh** (400 MHz, $CDCl_3$)

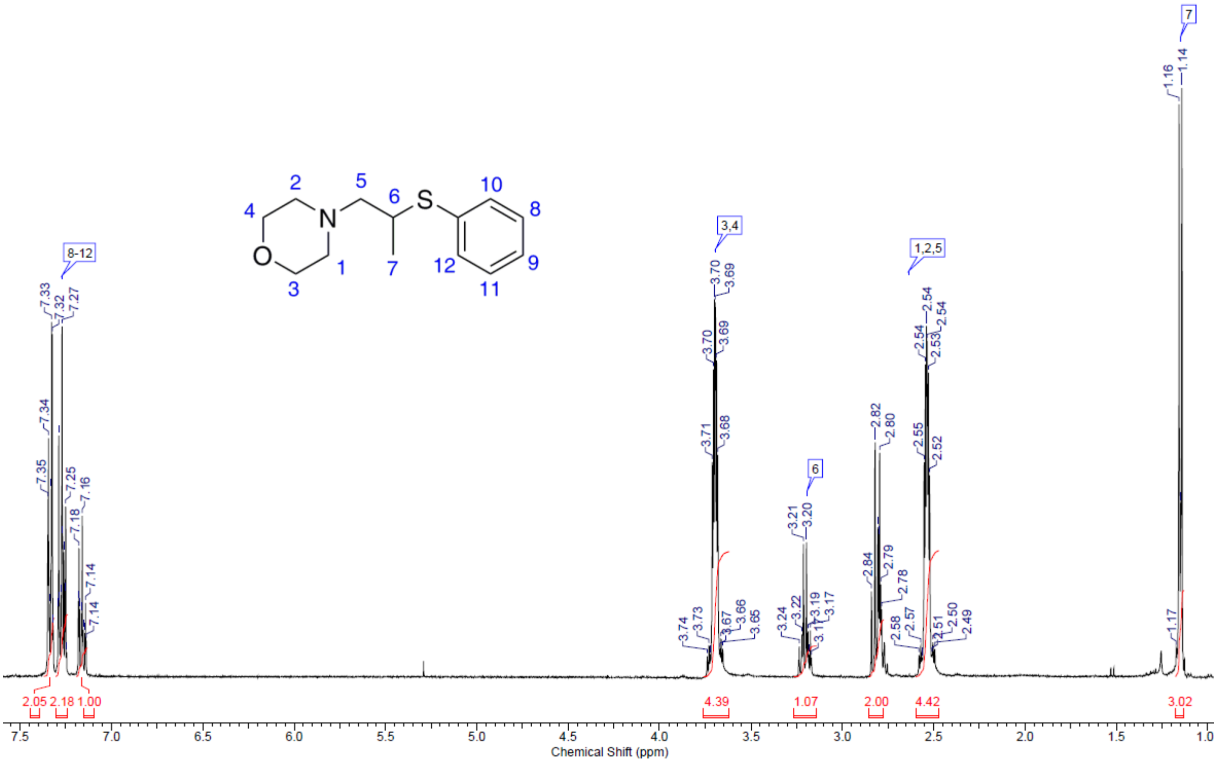

^{13}C NMR spectrum of *rac*-**5**–**SPh** (100 MHz, $CDCl_3$)

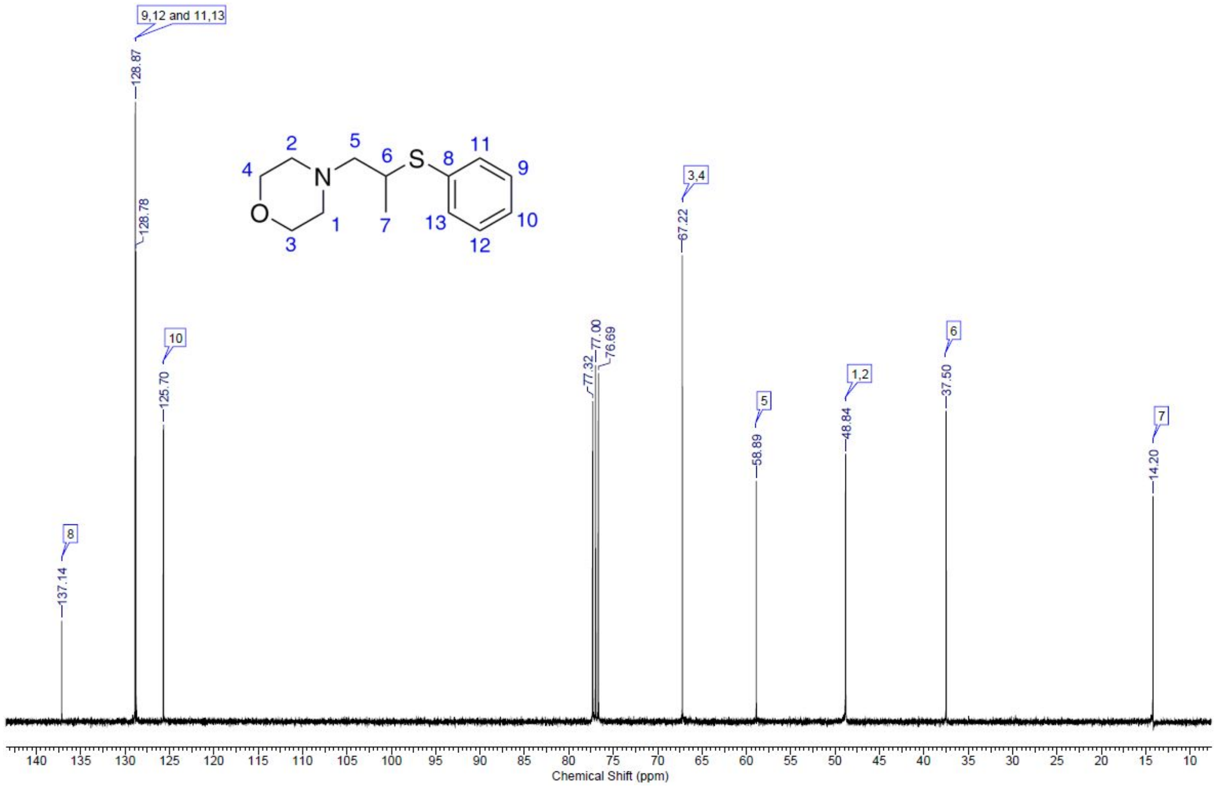

MS spectrum of *rac*-**5**–**SPh** (ESI-TOF)

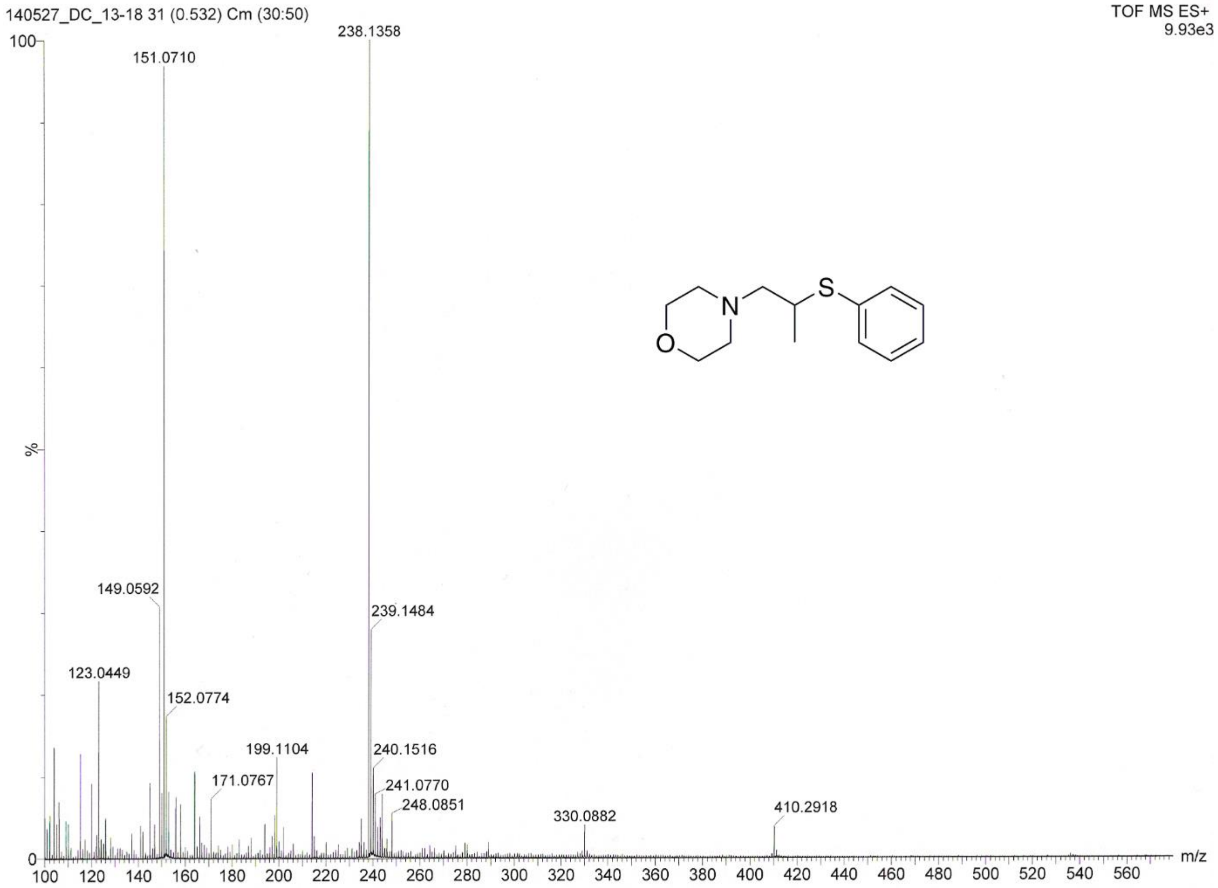

Diphenylacetic acid chloride (8)

^{1}H NMR spectrum of **8** (400 MHz, $CDCl_3$)

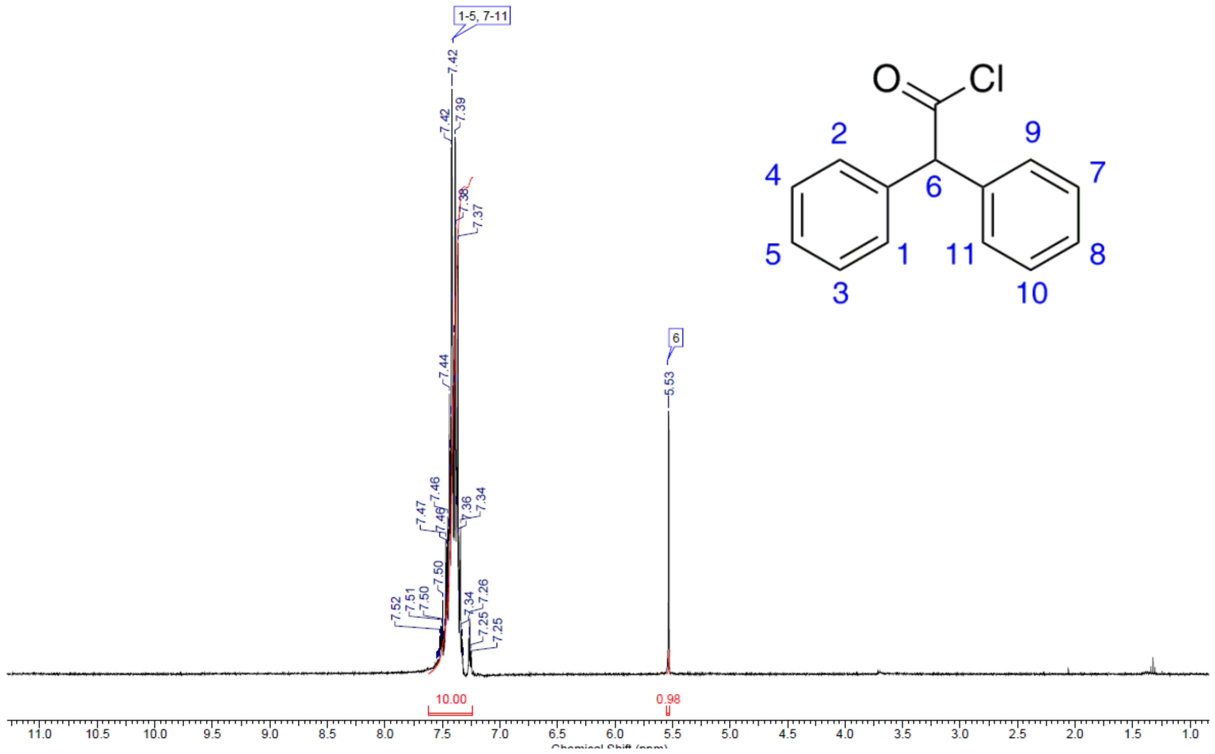

^{13}C NMR spectrum of **8** (100 MHz, $CDCl_3$)

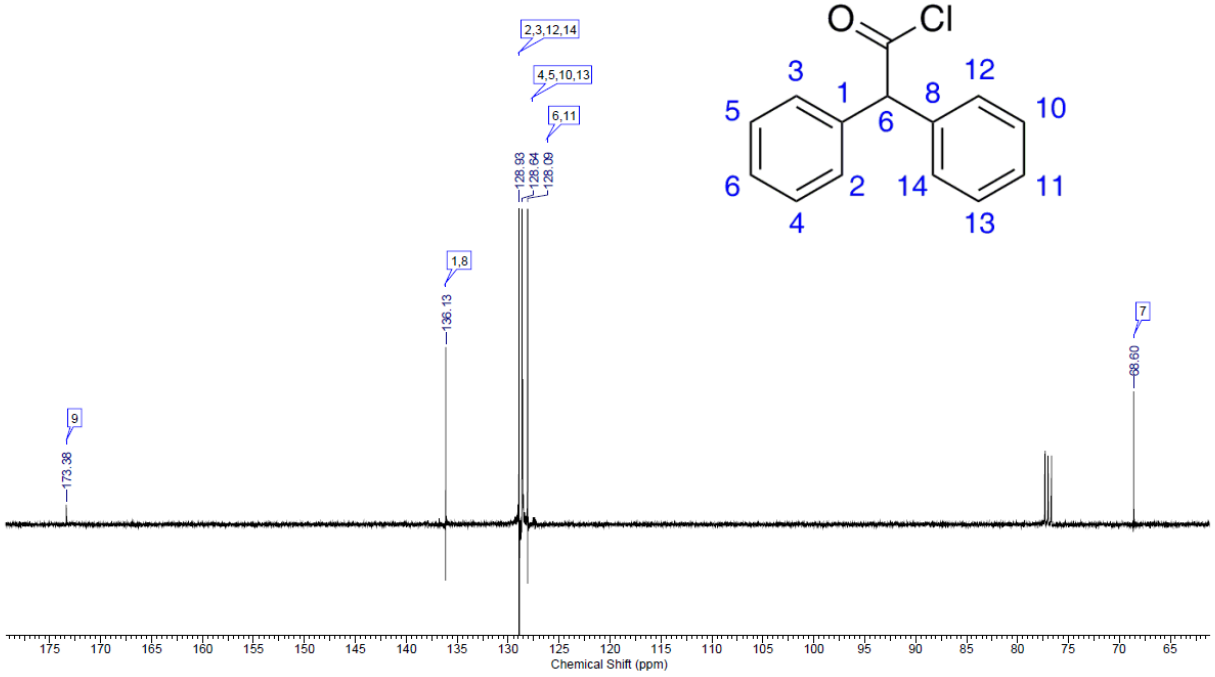

N-diphenylacetyl-1-pyrrolidine (9)

^{1}H NMR spectrum of **9** (400 MHz, $CDCl_3$)

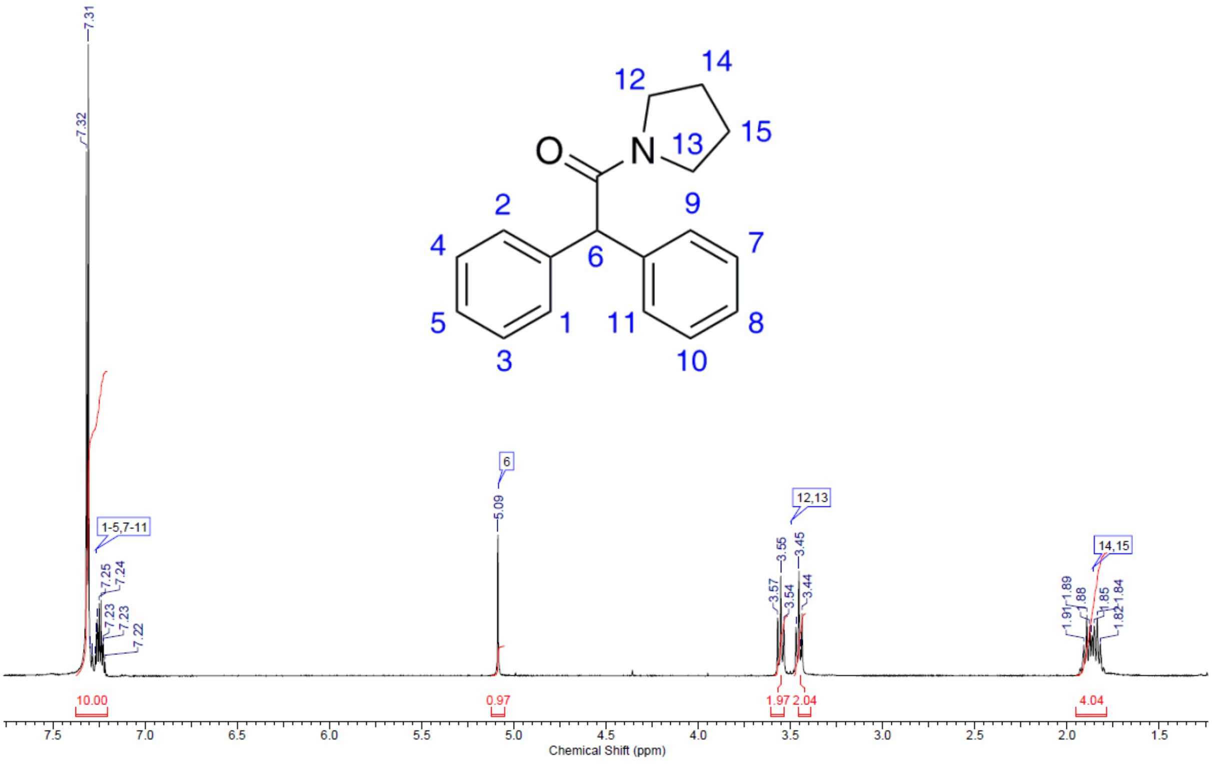

^{13}C NMR spectrum of **9** (100 MHz, $CDCl_3$)

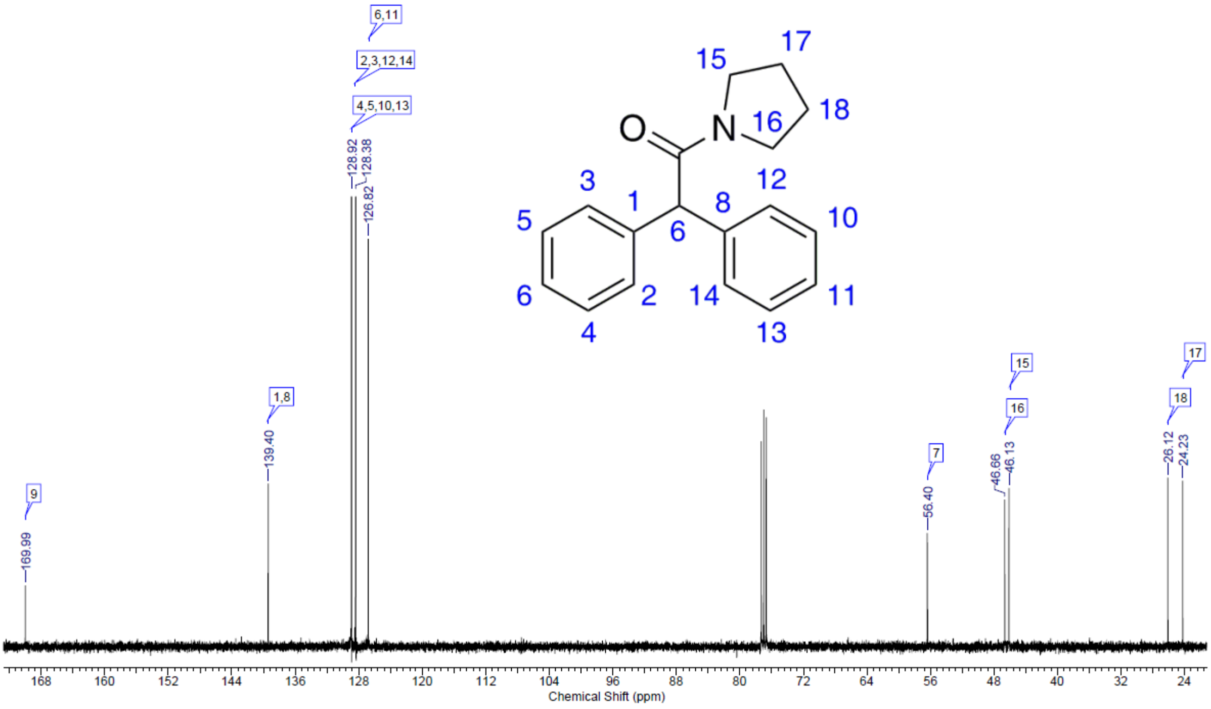

MS spectrum of **9** (ESI-TOF)

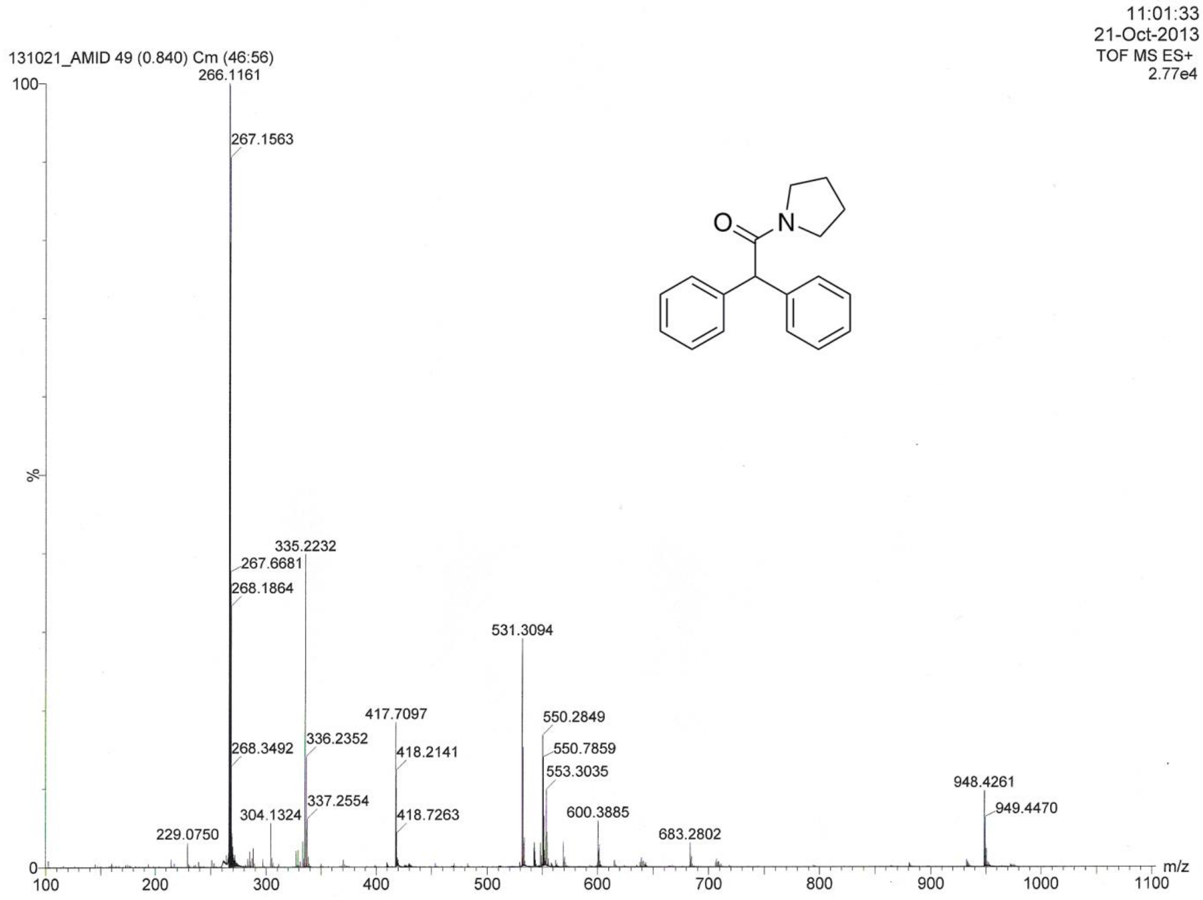

FT-IR spectrum of **9** (neat)

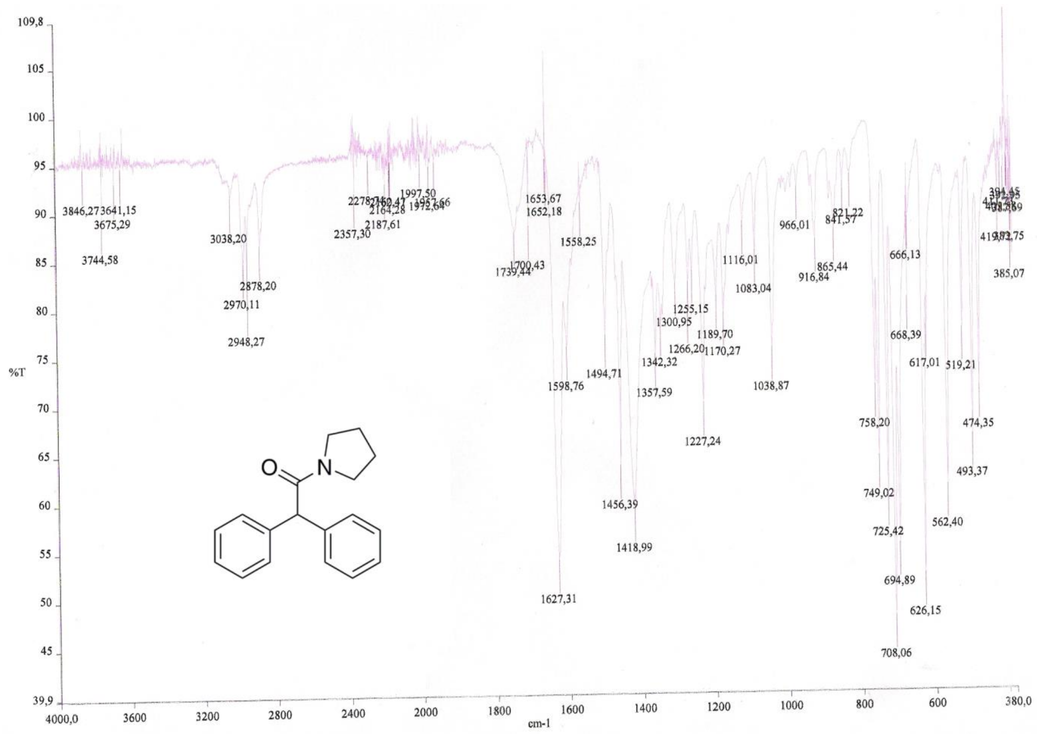

2-[2-(Morpholin-4-yl)propoxy]-2,2-diphenyl-1-(pyrrolidin-1-yl)ethan-1-one (*rac*-**10**)

^{1}H NMR spectrum of *rac*-**10** (500 MHz, CD_3CN)

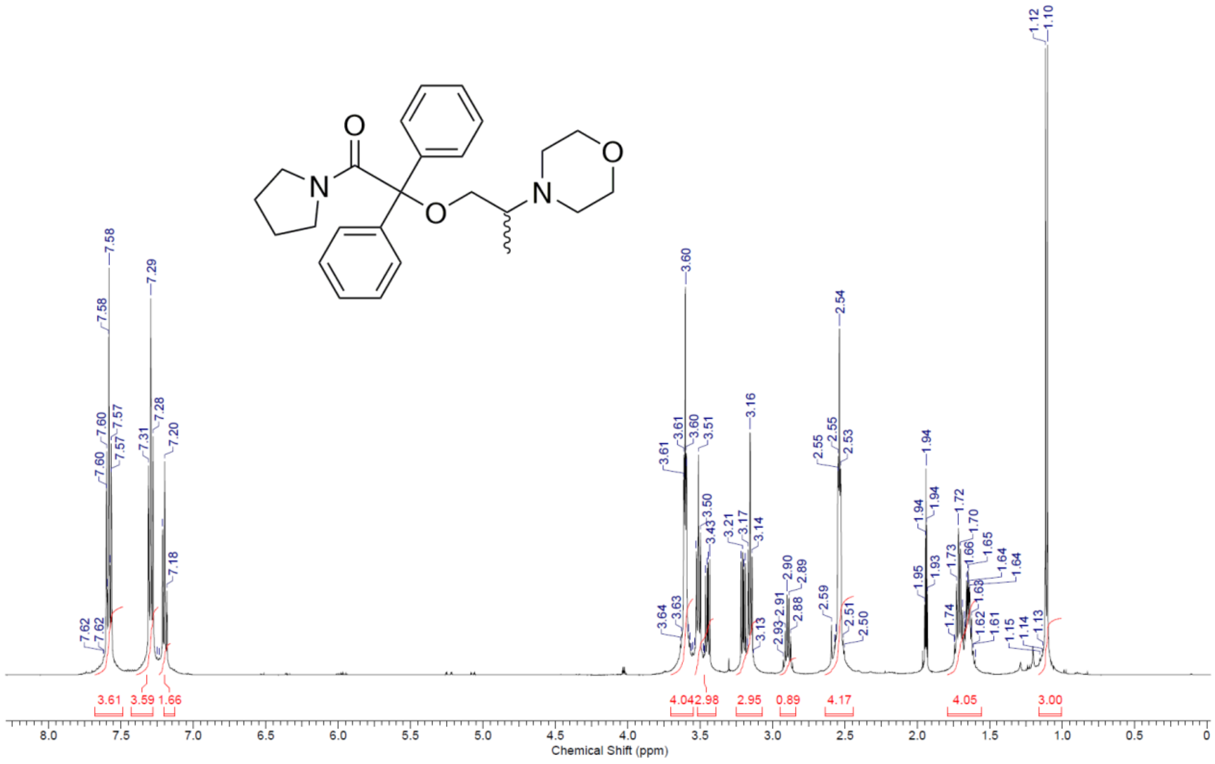

^{13}C NMR spectrum of *rac*-**10** (126 MHz, CD_3CN)

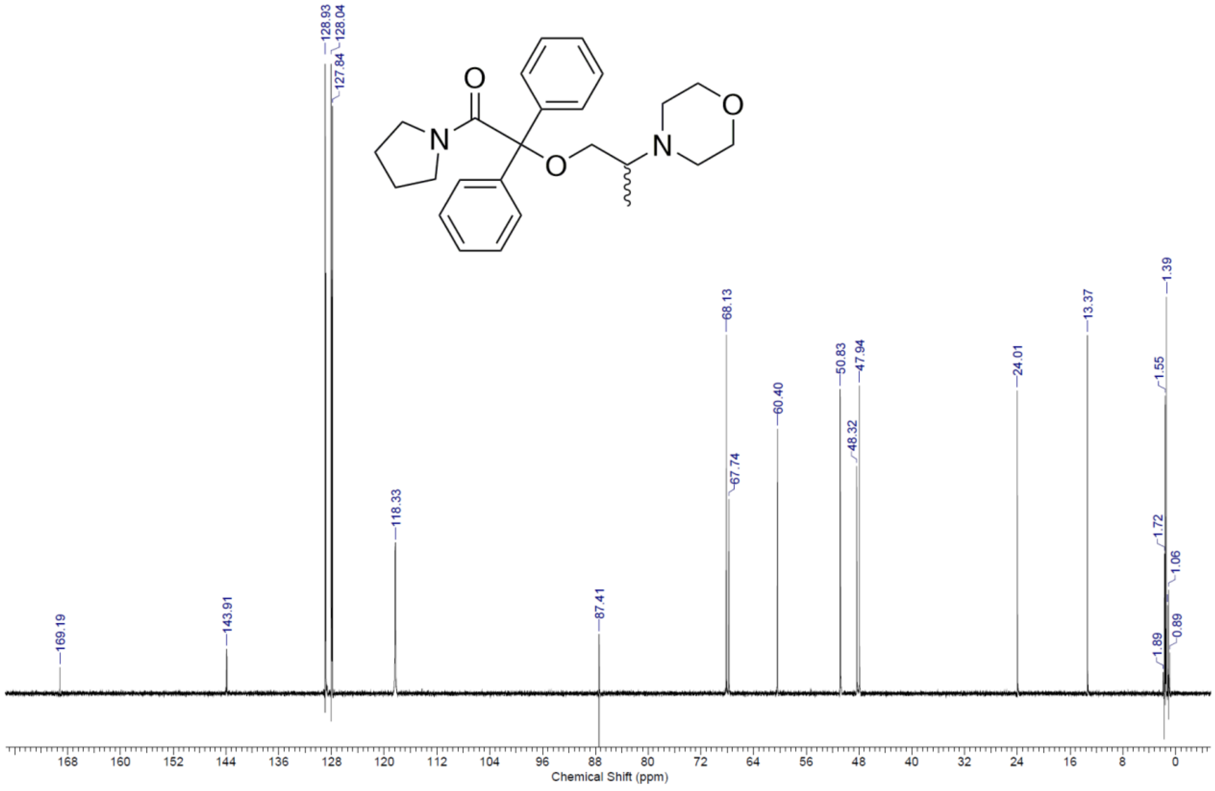

^{13}C DEPT-135 NMR spectrum of *rac*-**10** (126 MHz, CD_3CN)

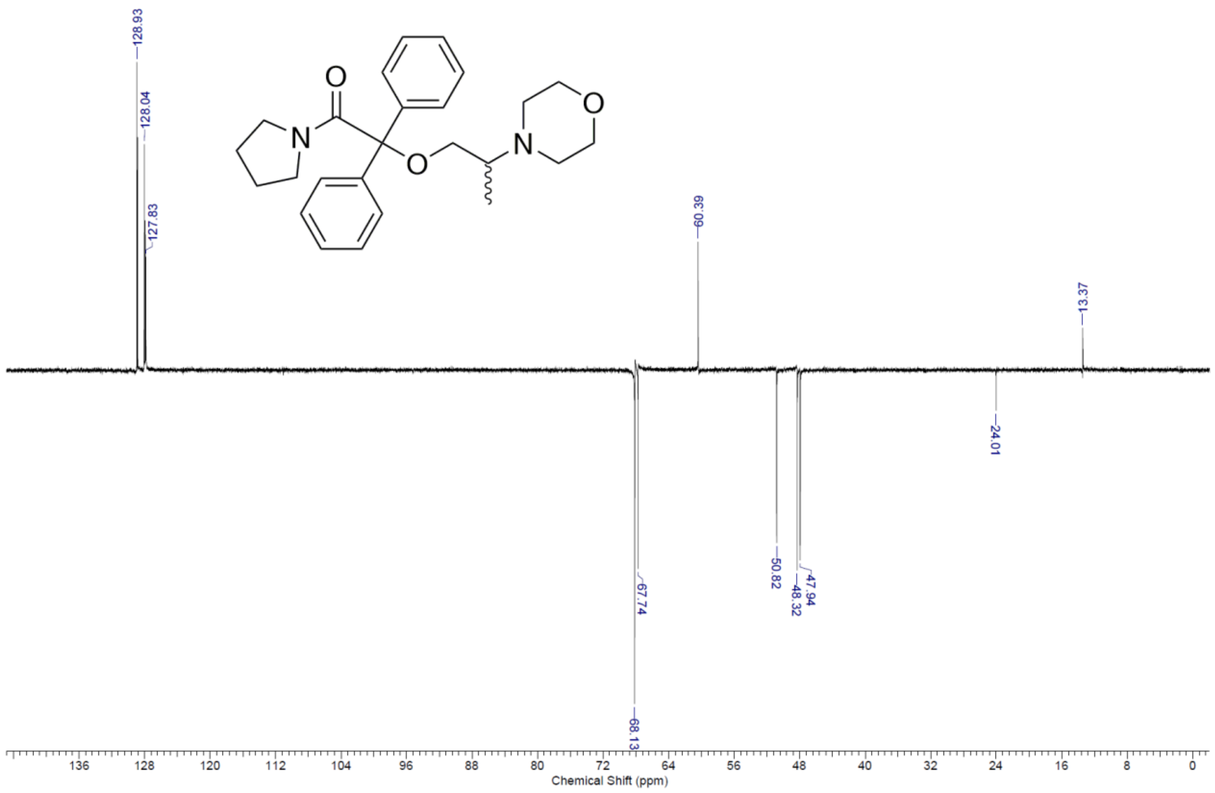

2D NMR (HSQC) spectrum of *rac*-**10** (126 MHz, CD_3CN)

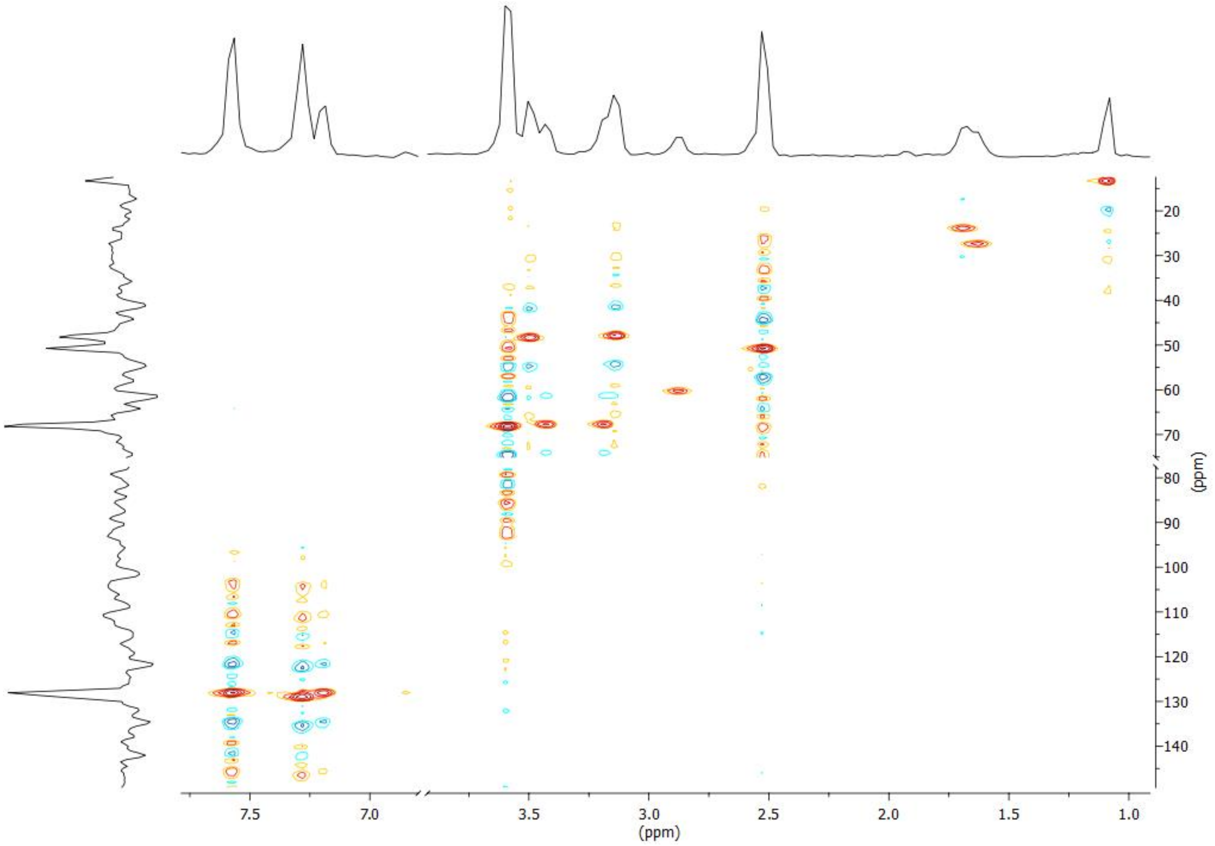

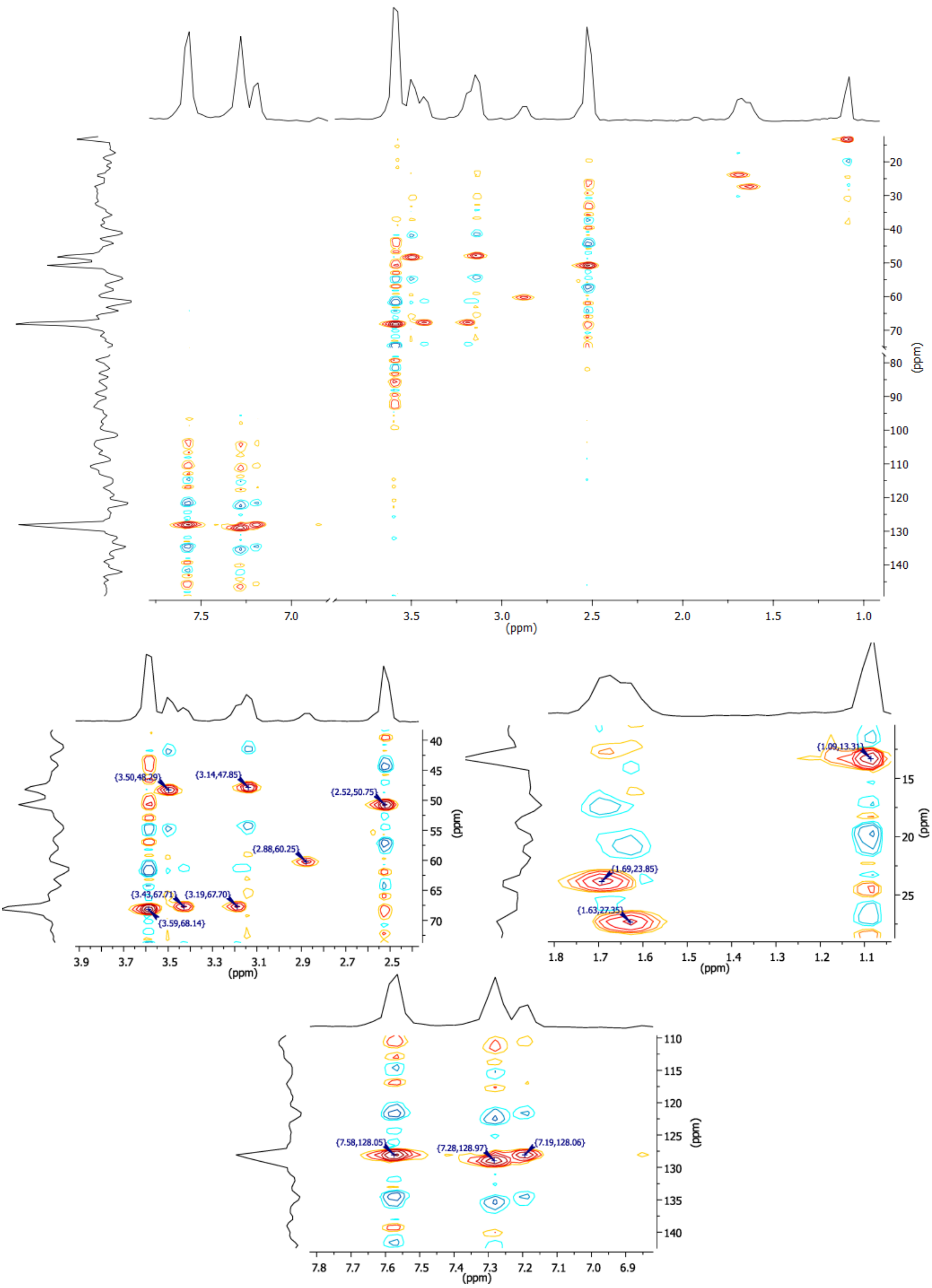
(ppm)
{3.50,48.29}
{3.14,47.85}
{2.52,50.75}
{2.88,60.25}
{3.43,67.71}
{3.19,67.70}
{3.59,68.14}
{1.09,13.31}
{1.69,23.85}
{1.63,27.35}
{7.58,128.05}
{7.28,128.97}
{7.19,128.06}

MS spectrum of *rac*-**10** (ESI-TOF)

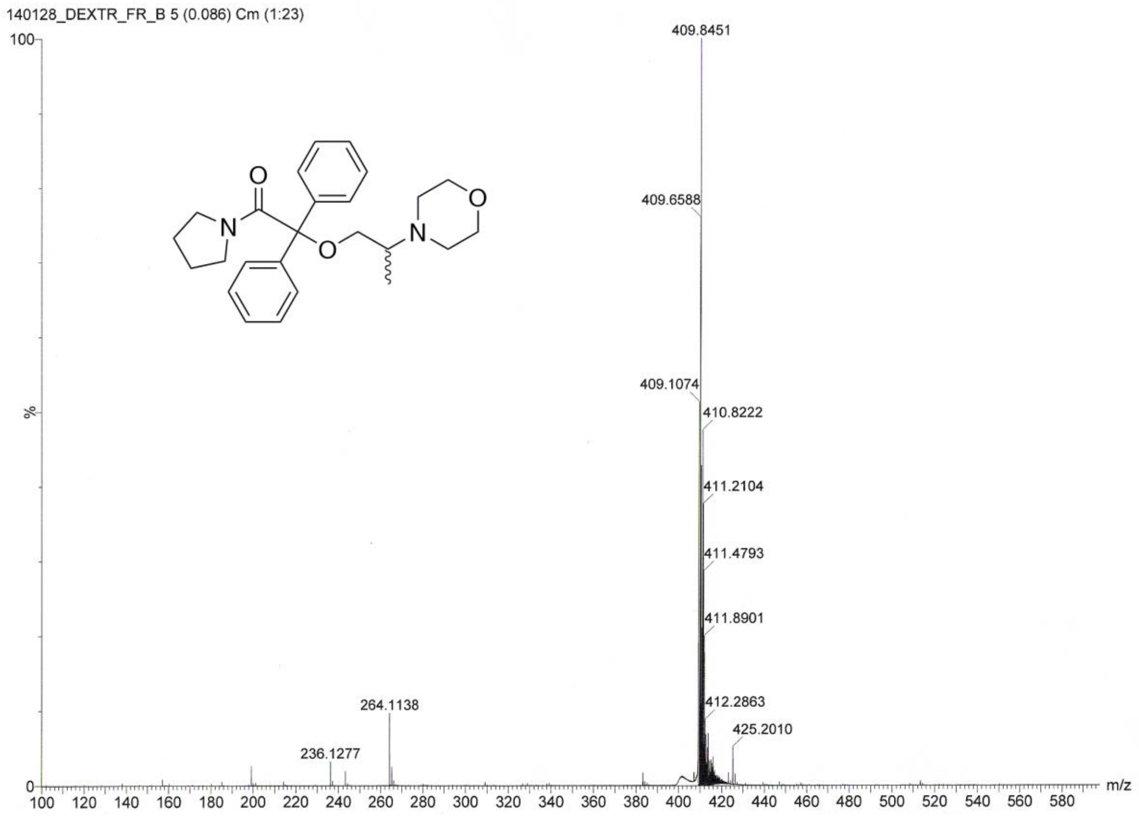

GC-MS spectrum of *rac*-**10** (EI)

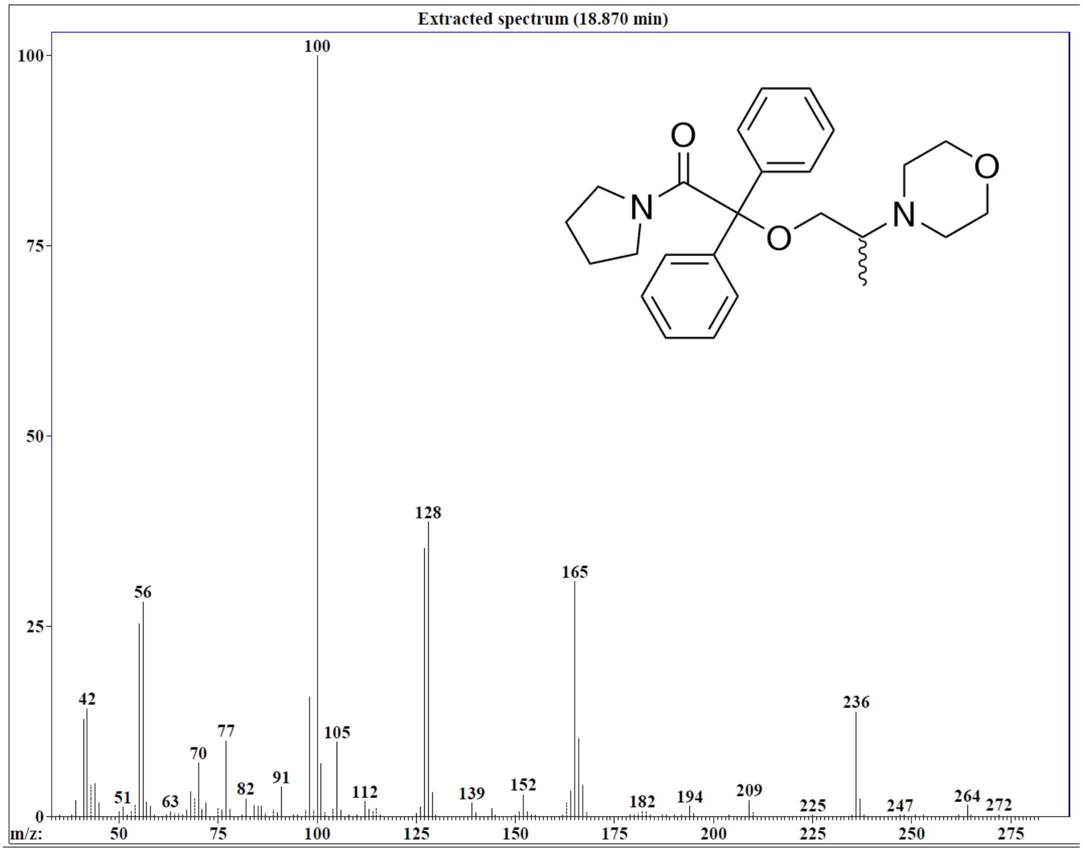

FT-IR spectrum of *rac*-**10** (neat)

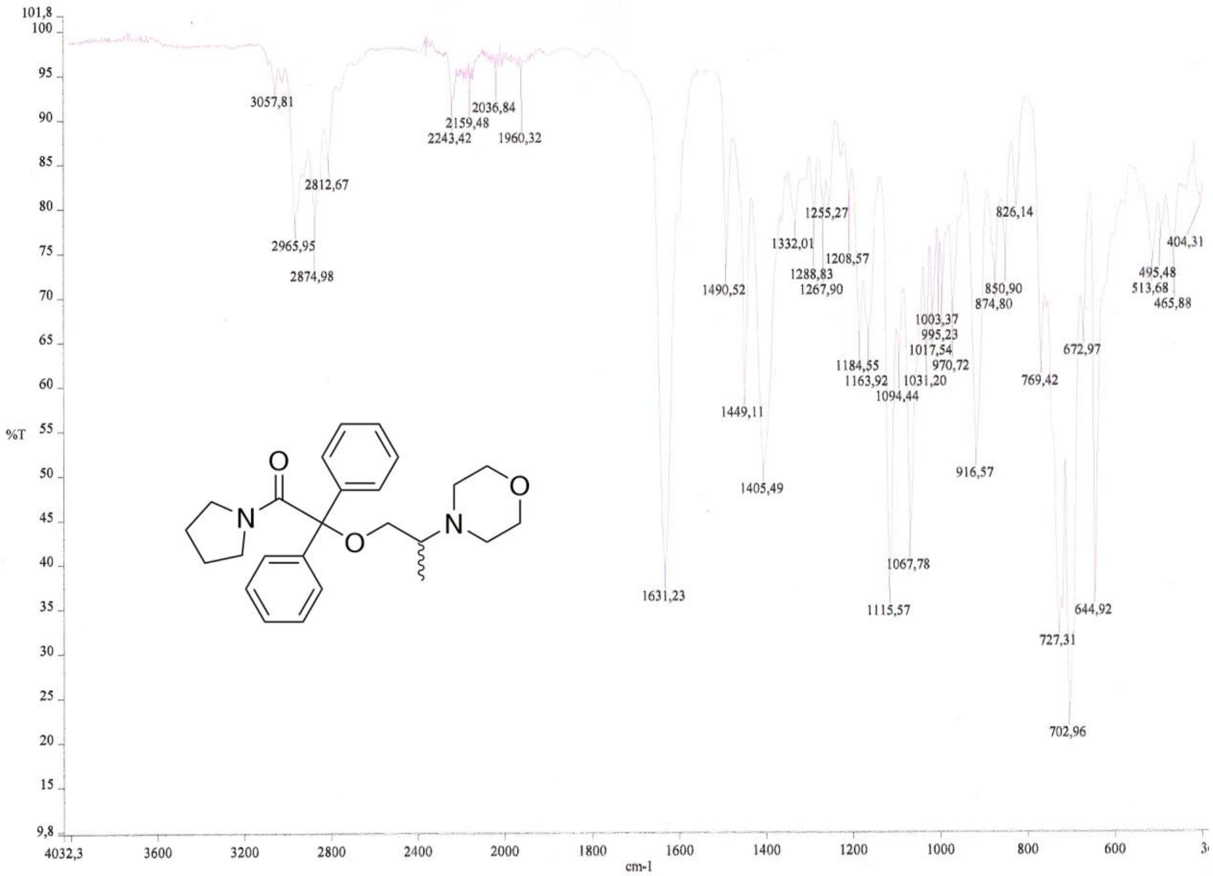

Supplement: Supplementary file 1 [file ijms-23-11803-s001.zip › ijms-1928071-supplementary.pdf]
